# Immune Gene Networks from Lung Cancer Patients Treated with Immune Checkpoint Inhibitors

**DOI:** 10.3390/biomedicines12030628

**Published:** 2024-03-12

**Authors:** Kyung Soo Kim, Taewon Kang, Dong Wook Jekarl

**Affiliations:** 1 Department of Thoracic and Cardiovascular Surgery, Seoul St. Mary’s Hospital, College of Medicine, The Catholic University of Korea, Seoul 06591, Republic of Korea; cskks@catholic.ac.kr; 2 Department of Laboratory Medicine, Seoul St. Mary’s Hospital, College of Medicine, The Catholic University of Korea, Seoul 06591, Republic of Korea; laurus@catholic.ac.kr

**Keywords:** immune gene expression, RNA-seq, network, lung squamous cell carcinoma, lung adenocarcinoma, immune checkpoint inhibitor, clustering coefficient

## Abstract

The association between immune checkpoint inhibitors (ICIs) and immune gene networks in squamous lung cancer (LUSC) and lung adenocarcinoma (LUAD) was studied. Immune gene networks were constructed using RNA-seq data from the gene expression omnibus (GEO) database. Datasets with more than 10 samples of normal control and tumor tissues were selected; of these, GSE87340, GSE120622, and GSE111907 were suitable for analysis. Gene set enrichment for pathway analysis was performed. For immune gene network construction, 998 unique immune genes were selected from 21 pathways in the Kyoto Encyclopedia of Genes and Genomes (KEGG). Gene function annotation was performed based on the KEGG, Gene Ontology, and Reactome databases. Tumor tissues showed decreased coagulation, hematopoiesis, and innate immune pathways, whereas complement- and coagulation-related genes were prominent in the tumor immune gene network. The average numbers of neighbors, clustering coefficients, network diameters, path lengths, densities, and heterogeneities were highest for normal tissue, followed by LUAD and LUSC. Decreased coagulation genes, which were prominent in tumor immune networks, imply functional attenuation. LUAD was deviated from normal tissue, based on network parameters. Tumor tissues showed decreased immune function, and the deviation of LUSC from normal tissue might explain LUSC’s better therapeutic response to ICI treatment.

## 1. Introduction

Lung cancer is the leading cause of death among malignancies and is second in incidence and fourth in prevalence worldwide [1]. In 2020, the global mortality and incidence of lung cancer were 18.0 and 22.4 per 100,000, respectively [1]. In the USA, lung and bronchus cancer were the second most common types among new cancer cases and had the highest mortality of 21% [2].

Immune checkpoint inhibitors (ICIs) disrupt the signaling of programmed death 1 (PD-1) receptors expressed on T cells and the PD, PD-L1, and PD-L2 ligands expressed on tumor cells [3,4,5]. These molecules are expressed in non-small-cell lung cancer (NSCLC) and result in inhibition of CD8^+^T-cell activation and immune escape [6,7]. Compared with conventional chemotherapy, ICI treatment shows better overall survival (OS) and progression-free survival (PFS) for squamous NSCLC (LUSC) and adenocarcinoma NSCLC (LUAD) [8,9,10,11,12]. Among ICIs, nivolumab is an IgG4 monoclonal antibody studied for lung carcinoma [8,9]. Median OS was 9.2 and 6.0 months for LUSC patients who received nivolumab and docetaxel, respectively, and it was 12.2 and 9.4 months among non-LUSC patients. One-year PFS (%) for nivolumab and docetaxel was 21% and 6% among LUSC patients and 19% and 12% for non-LUSC patients, respectively. The duration of response to nivolumab and docetaxel was 63% and 33% for LUSC, respectively, and 52% and 14% for non-LUSC (Supplementary Table S1). As that study was performed independently, the comparison of non-LUSC and LUSC did not exclude bias or deviation, but LUSC seemed to respond better to ICIs.

A recent meta-analysis of lung ICI monotherapy found that the hazard ratio (HR) for OS and the 95% confidence interval (CI) for non-LUSC and LUSC were 0.80, 95% CI 0.73–0.87 and 0.71, 95% CI 0.65–0.77, respectively. In addition, the HR (95% CI) for PFS was 0.90 (0.76–1.07) for non-LUSC and 0.65 (0.56–0.77) for LUSC [13]. These combined results for ICI monotherapy showed better OS and PFS for LUSC. ICIs have enhanced the overall survival and quality of life of patients with NSCLC. The efficacy of ICIs for NSCLC has been studied, but the underlying difference concerning histology has rarely been reported.

Network analysis can be applied to study complex systems with multiple variables [14,15]. Constructing mathematical models and calculating topological parameters can reveal biological systems with functional relationships [16,17]. For the immune system, the co-expression of immune gene networks could better elucidate the functional aspects of a complex system that cannot be interpreted via a single or several genes [18,19,20].

In this study, to identify the difference in ICI response between LUAD and LUSC, gene set enrichment for pathway analysis was followed by the construction of immune gene networks using RNA-seq from public data.

## 2. Materials and Methods

This was a retrospective study using datasets from the public Gene Expression Omnibus (GEO) database. This study was approved by the Institutional Review Board of Seoul St. Mary’s Hospital. The search for adequate datasets was performed from January 2023 to February 2023 with the keywords lung cancer, *Homo sapiens*, expression profiling by high throughput sequencing, and RNA sequencing, which resulted in 493 datasets. Among them, studies using human primary lung tissue with more than 10 control or normal lung cancer tissues were identified.

**Figure 1:**
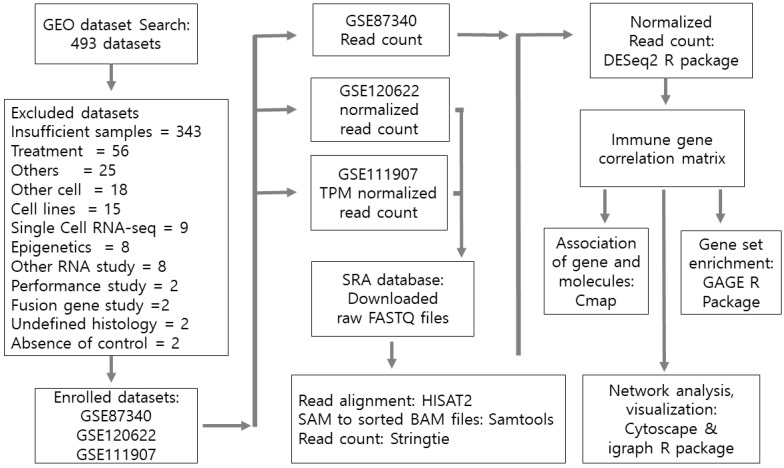
Flow chart of this study. The search was performed using the following terminology: The search resulted in 493 datasets; among them, 3 were recruited for study and the others were excluded.

GSE87340, GSE120622, and GSE111907 were selected for analysis [21,22,23] (Figure 1). As the purpose of this study was to compare normal tissue and lung cancer networks for immune gene expression, normal control data were required. Datasets studying cell lines, therapeutics, or RNA other than coding genes were excluded. Raw count data could be downloaded for GSE87340, but the GEO dataset provided normalized count data for GSE120622 and transcript per million (TPM) values for GSE111907, which were not suitable for analysis. Therefore, the raw Fastq dataset was downloaded from the sequence read archive (SRA) database for GSE120622 (PRJNA463790, SRP162843) (Supplementary Table S2) and GSE111907 (PRJNA438518, SRP255477) (Supplementary Table S3). Sratoolkit, provided by the SRA database, was utilized to download RNA-seq fastq files. HISAT2 was utilized to align raw Fastq files with GRCH38 genomic sequences [24]. Samtools was used to convert SAM files to BAM files and to sort the BAM files. Stringtie was used to calculate the read count data [25]. The versions of the software used are listed in Supplementary Table S4.

### 2.1. Gene Set Enrichment for Pathway Analysis

After selecting normal controls and an equal number of tumor tissues, gene set enrichment for pathway analysis was performed using the GAGE R package [26], which utilizes the Kyoto Encyclopedia of Genes and Genomes (KEGG) pathways [18,27]. Significant pathways showing a global *p*-value less than 0.05 were plotted as heatmaps and immune-related pathways were plotted.

### 2.2. Network Construction

For network analysis, data from diseased tissues were selected according to the corresponding normal data. From 21 pathways in KEGG, 998 unique immune genes were selected as previously reported [25]. Gene function annotations based on KEGG, Gene Ontology, and the Reactome database were categorized as adaptive immunity, antigen presentation, cytokines–chemokines, complement, hematopoiesis, innate immunity, leukocyte migration, NK cell activity, platelet activity, and signaling [28,29]. If the annotated frequency was comparable between groups, the chemokine–cytokine and signaling or hematopoiesis pathways were selected, followed by other pathways [18].

Cases for network analysis were selected to match the available number of normal controls with an equal number for LUAD or LUSC. LUAD and LUSC cases were selected in sequence for network analysis.

Networks were constructed based on a Pearson’s correlation coefficient greater than or equal to 0.95 and a *p*-value less than 0.05 for each immune gene. Each immune gene was regarded as a node, and the correlation pairs between genes were regarded as links. Links were undirected and created based on correlation coefficients with statistical significance. If the carcinoma patients were outnumbered by the controls, carcinoma samples were selected based on random selection without replacement to match the number of control samples. These immune gene correlation networks were constructed using Hmisc and ggplot2, an R package [30,31], and plotting of the constructed networks and calculation of the topological parameters were performed using the Cytoscape Network Analyzer [32,33]. Among the network topological parameters, assortativity and modularity were calculated via igraph [34,35]. All statistical analyses were performed using R version 4.2 and related packages or Cytoscape version 3.9.1 [36,37].

### 2.3. Network Topological Analysis

The network topological parameters were calculated [14,15,16,17] and provided global quantitative aspects as follows [38,39]: a node (*N*) indicates an immune gene; a link (*L*) indicates a paired correlation between the nodes plotted in a line. The hub node has the maximum number of links within the network. The degree (*k*) is the number of links connected to the node, and the average degree (<*k*>) is the average number of links connected to a node in a network. Connected components are the number of clusters of nodes connected to each other.

The diameter is the maximum length of the shortest path between two nodes. The characteristic path length is the average of the shortest path length. The average number of neighbors is the number of identical nodes connected to the node of interest, and the normalized average number of neighbors is the network density. The clustering coefficient is the number of links by which neighbors of a given node are connected to each other and can be considered friends of a friend. The local clustering coefficient (*C*) for a given node *i* with *k_i_* degree was calculated as follows: *C_i_* = 2*Li*/*k_i_* (*k_i_* − 1), where *C_i_* denotes the local clustering coefficient, *Li* denotes the number of links between the neighbors of *k_i_*, and *k_i_* denotes the number of degrees *k_i_* for a given node *i*. The average clustering coefficient (<*C*>) represents the average of *C_i_*, which is calculated as follows: <*C*> = 1/*N* $\sum_{i=1}^{N}C_{i}$. If *C_i_* = 0, there are no friend’s friends, or the neighbors of a given node are not linked. Density (*d*) is the number of links within a network divided by the total possible links between nodes, which was calculated as *d* = *L*/*L_max_*, where *L* denotes the number of links and *L_max_* denotes the possible number of links for a given node. Heterogeneity is the diversity of the number of links in a node or the coefficient of variation of the number of edges in a node, which was calculated as follows: heterogeneity = $\sqrt{variance(k)}$/mean (*k*). Centralization (degree) was defined as the importance of a node based on the number of links, which was calculated as *C* = $\sum_{j=1}a$*_ij_*/*n* − 1, where *a_ij_* is the adjacency matrix and *n* is the total number of nodes. The degree of distribution is the number of nodes with degree *k.* Modularity denotes the tendency of nodes to cluster and form groups that could divide the network into communities and was calculated as follows: *Q* = 1/2*L* $\sum_{ij}($*A_ij_* − $\frac{k_{i}k_{j}}{2L}$)*δ*(*C_i_, C_j_*), where *L* is the number of links, *A_ij_* is the adjacency matrix, *k_i_* or *k_j_* is the degree of *i* or *j,* and *C_i_* and *C_j_* are the components of *i* and *j*, respectively. The assortativity coefficient denotes the association of the nodes that tend to be related to similar nodes, which is basically a Pearson’s correlation coefficient of the degree, and is calculated as follows: *r* = 1/$\sigma_{q}^{2}$ $\sum_{jk}$*jk* (*e_jk_ − q_j_q_k_*), where *e_jk_* is the fraction of links that connect node *i* to one of type *j*, *q_j_* and *q_k_* are the fraction of each type of end of an edge, and $\sigma_{q}$ is the standard deviation of a distribution *q_k_*. For the calculation of network similarity using topological network parameters, the distance between normal and LUAD or LUSC data points was calculated utilizing Euclidean, Manhattan, and cosine distance.

### 2.4. Connectivity Map Analysis

The gene signatures selected for network analysis were reviewed using connectivity map analysis (https://clue.io) accessed 19 February 2023 on to search for associations between genes, small molecules, treatment therapeutics, and disease states [40]. The connectivity map was based on databases including drug-specific gene expression associated with disease-specific gene signatures. The connectivity score was calculated by dividing the connection strength by the maximum connection strength of a given gene and the reference gene profiles, ranging from −1 to 1. A value of 1 indicates maximum positive connection, while −1 indicates negative connection [40,41]. Among the network gene expression signatures, we selected the top 100 genes for analysis and entered them onto the CMAP website.

## 3. Results

The baseline characteristics of datasets that include studies of lung cancer using RNA sequencing with normal controls (GSE87340, GSE120622, and GSE111907) are listed in Table 1. GSE87340 only included LUAD patients along with normal tissue, while GSE120622 included LUAD as well as LUSC. GSE111907 was distinct in that the authors sorted the cells by flow cytometry using CD10^+^EPCAM^−^CD45^−^CD31^−^ (fibroblasts), CD31^+^CD45^−^EPCAM^−^ (endothelial cells), CD45^+^EPCAM^−^ (immune cells), and EPCAM^+^CD45^−^CD31^−^ (malignant cells).

Gene set enrichment for pathway analysis showed increased T-cell receptor- and Fc gamma receptor-mediated phagocytosis but decreased coagulation and hematopoiesis pathways in most of the tumors (Supplementary Table S5 and Supplementary Figures S1–S5).

**Figure 2:**
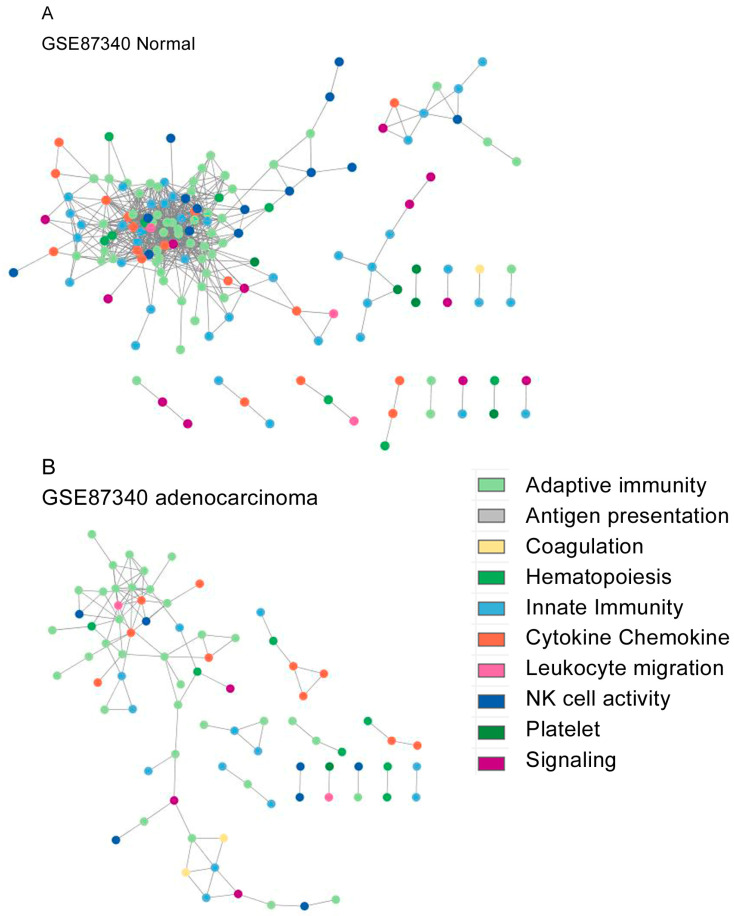
Network topological structure of GSE87340 from (**A**) normal tissue and (**B**) LUAD tissue.

**Figure 3:**
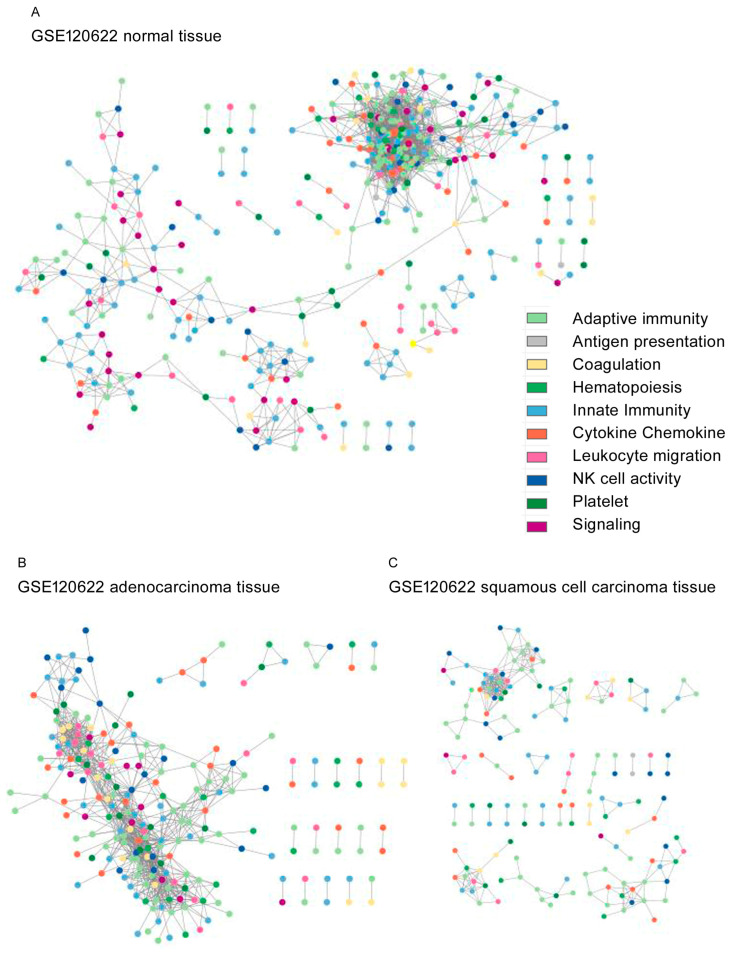
Topological network structure of GSE120622 from (**A**) normal tissue, (**B**) LUAD tissue, and (**C**) LUSC tissue.

**Figure 4:**
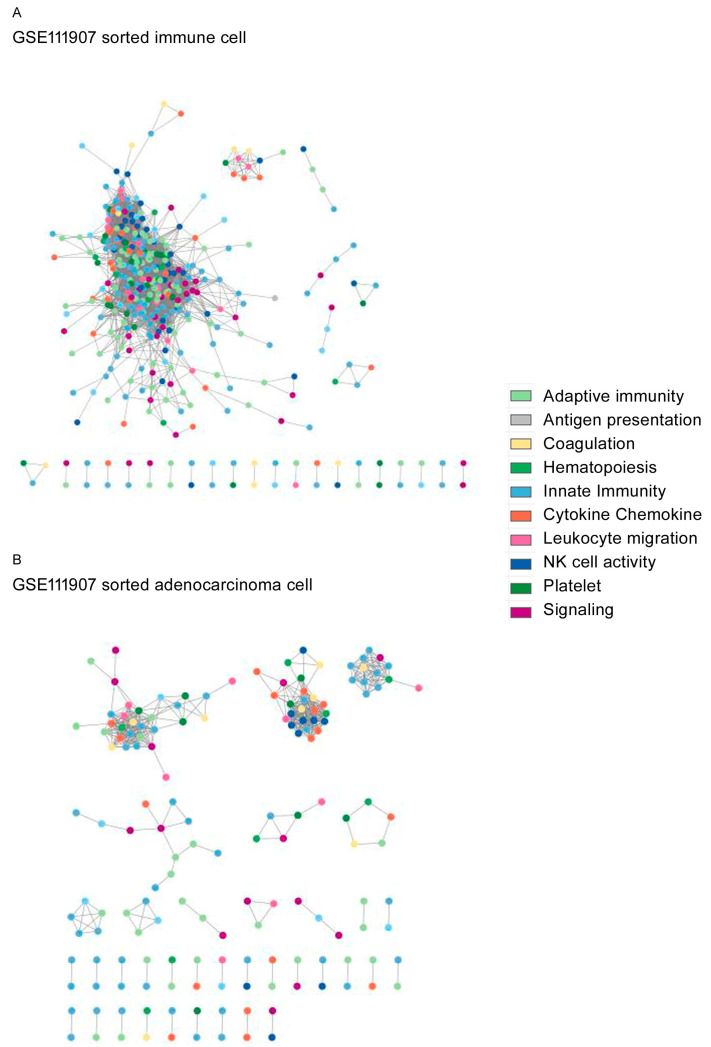
Topological network structure of the (**A**) sorted immune cells and (**B**) sorted LUAD for GSE111907-1.

**Figure 5:**
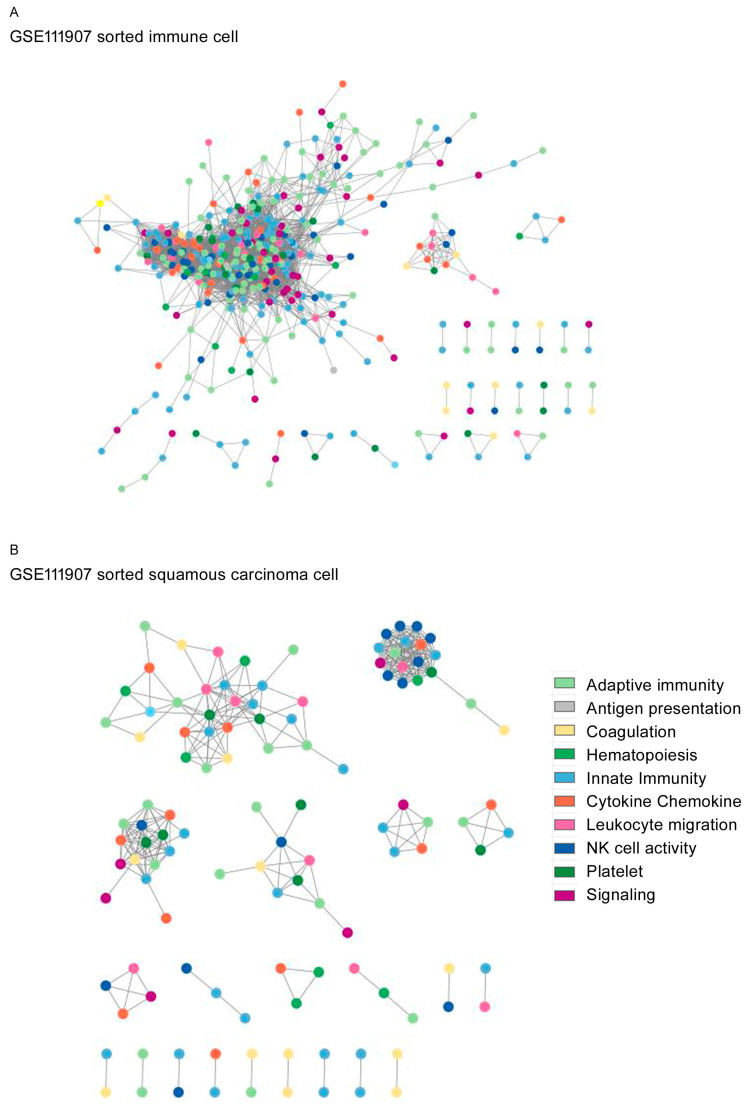
Topological network structure of the (**A**) sorted immune cells and (**B**) sorted LUSC for GSE111907-2.

The topological network structures are shown for GSE87340 (Figure 2), GSE120622 (Figure 3), and GSE111907-1 with LUAD (Figure 4) and for GSE111907-2 with LUSC (Figure 5). The common topological structure revealed larger networks with more diverse genes in the normal lung tissue compared with LUAD or LUSC tissue. For GSE111907, sorted immune cells were plotted; fibroblast and endothelial cells along with tumor cells are plotted in Supplementary Figure S6 for LUAD and in Supplementary Figure S7 for LUSC.

The genes in the constructed networks showed prominent ratios of coagulation genes, but innate-immunity-related genes were less prominent in the tumor tissue and sorted tumor cells (Supplementary Table S6).

The range of coagulation-related genes included in networks from normal tissue was 1% to 4%, while that in tumor tissue or carcinoma cells was 3% to 11% (Supplementary Figures S8 and S9). The range of innate-immunity genes included in networks from normal tissue was 25% to 30%, while that in tumor tissue or carcinoma cells was 16% to 31%. The range of signaling genes included in networks from normal tissue was 8% to 11%, compared with 2% to 9% in tumor tissue or carcinoma cells (Supplementary Figures S8 and S9).

The topological network parameters showed a higher average number of neighbors, characteristic path length, network density, network heterogeneity, and centrality (Table 2) for normal tissue and sorted immune cells, followed by LUAD and LUSC. Except for assortativity and modularity, most of the parameters were lowest in LUSC and highest in normal tissue.

The clustering coefficient was reported to be higher in normal tissues compared with malignancies [37]; in the present study, the clustering coefficient was higher for normal tissue and for sorted immune cells in general. These results imply that the network parameters can represent the immunological status of tissues. LUSC tissue and sorted tumor cells are thought to have relatively enhanced immune activity compared with AS-NSCLC.

The hub genes (*k* degree, annotated function) for each constructed network were as follows: GSE87340 normal tissue, *JAK3* (*k* = 43, adaptive immunity); GSE87340 LUAD, *CD3E* (*k* = 10, adaptive immunity); GSE120622 normal tissue, *VAV1* (*k* = 74, chemokine); GSE120622 LUAD, *CD4* (*k* = 33, adaptive immunity); GSE120622 LUSC *ITGAM* (*k* = 18, leukocyte migration); GSE111907-1 sorted immune cells, *CD5* (*k* = 87, hematopoiesis); LUAD, *PTGIR* (*k* = 20, platelet); GSE111907-2 sorted immune cells, *CD5* (*k* = 85, hematopoiesis); and LUSC, *F2RL3* (*k* = 16, platelet).

The coagulation pathway was decreased in tumor tissue compared with normal tissue, and complement–coagulation genes were prominent in the network topology from tumor tissues. These results imply that decreased expression of coagulation genes plays an important role in carcinogenesis.

Topological network analysis showed that normal tissue had the highest network parameters, followed by LUSC and LUAD tissue. These results suggest that immune status and immune activity are higher for LUAD compared with LUSC. Altogether, the study results may explain LUSC’s favorable response to ICI treatment compared with that of LUAD.

The connectivity map revealed various small molecules associated with gene expression network signatures (Supplementary Figure S10). We selected A549 and HCC515, which is a LUAD origin cancer cell line. *IKZF1*, an IKAROS family zinc finger protein, was selected for the GSE120622 LUAD dataset. The molecules suggested by the connectivity map included various targets and associated small molecules for datasets (Supplementary Tables S7–S11).

The Euclidean, Manhattan, and cosine distances from normal to LUAD and LUSC were as follows: Euclidean, 1642.6, 2396.1; Manhattan, 1831.8, 2656.9; and cosine, 0.0024, 0.0513, respectively. These analysis results indicate that LUAD tissues are closer to normal tissues compared with LUSC, and LUSC tissues deviated more from the normal samples.

## 4. Discussion

The application of cancer immunotherapy has achieved clinical advances and proven antitumor effects for melanoma, NSCLC renal cell carcinoma, head and neck squamous cell carcinoma, urothelial cell carcinoma, colorectal cancer, and others [13,42,43]. The effect of ICIs on histological types of NSCLC has been reported, and a meta-analysis revealed that both non-LUSC and LUSC responded to ICIs, with LUSC showing a greater response [13]. However, another meta-analysis of smaller size reported that non-LUSC showed a greater response to ICI treatment in previously untreated patients [43]. These differences in meta-analysis results may have been caused by selection bias, analytical methods, and the criteria for response. The LUSC group seemed to show a greater magnitude of response compared with the non-LUSC group. However, the underlying mechanism of ICI response depending on histologic type has rarely been reported.

Network analysis could provide comprehensive quantitative results for complex systems, including RNA-seq or metabolite analysis [44]. Finding a single causative variable might explain the pathophysiology in a more intuitive manner. Unlike these reductive methods, network analysis considers given variables and analyzes the relationship between them. By calculating the topological network parameters, global quantitative values could represent these systems.

The network construction for normal tissue showed that genes with various functions comprised the immune network, and the network diameter was larger than for tumor tissue or sorted tumor cells. Complement- and coagulation-related genes were the most prominent, and innate-immune genes were less prominent within tumor tissue. In addition, pathway analysis showed that coagulation pathways were decreased in most of the studied tumor tissues. These results suggest that coagulation-related genes or pathways are significantly associated with other genes whose expression was decreased in most of the tumor cells. Decreased innate-immune genes along with prominent complement- and coagulation-related genes in the immune network may be related to carcinogenesis. The down-regulation of the hematopoiesis pathway in most of the cancer tissues was noted, which is thought to be the effect of the proliferative nature of tumor cells and the suppression of immune cell proliferation or differentiation. These results show that immune functions are thought to be decreased in tumor tissue, along with proliferation and differentiation of immune cells.

These immune networks might reflect the tumor microenvironment or features of tumor cells and ICI responses [41]. One proposed mechanism for immune responses is that CD8^+^ T cells secrete interferon-γ to kill tumor cells and also induce PDL1 on tumor cells, which binds PD1 on CD8^+^ T cells to mitigate the tumor-eliminating response. The tumor microenvironment might be associated with increased cytokines from regulatory T cells that secrete immunosuppressive cytokine–chemokines, such as TGF-beta and IL10, which suppress the activation, proliferation, and functions of CD8^+^ T cells. Tumor cells that secrete VEGF upregulate immune-suppressive molecules such as CTLA4, LAG3, and TIM3.

The network parameters revealed that the average neighbor, network diameter, radius, path length, clustering coefficient, density, heterogeneity, and centrality of normal tissue and LUAD were more similar compared with those of normal tissue and LUSC. Indeed, the similarity and distance between normal tissue and LUAD were more similar compared with normal tissue and LUSC. From these data, we suppose that these similarities might be an explanation for the better ICI responses in LUSC. The deviation of LUSC from normal topological parameters might be related to better immune response after ICI treatment [36,37].

The hub nodes were inconsistent between normal tissue and cells and tumor tissues and tumor cells. It is not clear why hub genes are heterogeneous, but it could reflect a variation in immune function at the point of sample collection. However, the hub nodes for normal tissue and cells had a higher degree compared with those of tumors. This might be related to the active immune function in normal tissue and cells. In addition, the average number of neighbors was increased in normal tissue and cells, which may be related to increased immune function.

The connectivity map revealed that the *IKZF1* (IKAROS zinc fingers) gene showed a significantly high connectivity score in the LUAD cell line. About 6.4%, 1.0%, and 7.5% of LUAD patients showed *IKZF1* gene mutation, deletion, or gene amplification, whereas 6.8%, 2.5%, and 5.0% of LUSC patients in previous studies showed gene mutation, deletion, or gene amplification [3,45]. *IKZF1* is a transcription factor that is the master regulator of lymphocyte development. *IKZF1* gene mutation/deletion and gene amplification are reported to be related to the mitigated and enhanced efficacy of ICIs, respectively. Genomic alteration of *IKZF1* predicts poor prognosis and low infiltration by immune cells in tissue, which is incongruent with the results of this study [46,47]. This might be derived from the fact that this study considered not merely one gene but the whole network. Further studies are required for verification of the role of the *IKZF1* gene in ICI response.

One limitation of this study was that it was performed using public datasets, which might have been established in different contexts and with different patient characteristics. As this study was focused on the construction of immune gene networks, these mathematical models require verification based on biological experiments to understand the exact mechanism of ICI response and histological differences in non-small-cell lung carcinoma.

## 5. Conclusions

In conclusion, tumor tissues showed a decrease in coagulation, innate-immune genes, and hematopoiesis pathways compared with normal tissue or cells. Complement- and coagulation-related genes were prominent in the immune network, which implies that tumor tissue and cells exhibit a decrease in coagulation genes, thereby affecting other immune genes in the tumor tissue. The topological parameters showed that LUSC deviated from normal tissue compared with LUAD. This implies that LUSC tissues are biologically deviated, which might provoke enhanced immune and immune checkpoint inhibitor responses.

## Figures and Tables

**Table 1:** Baseline characteristics of downloaded GEO datasets.

	GSE87340	GSE120622		GSE111907
	LUAD	LUAD	LUSC	LUAD/LUSC
Cases (n)	27	43	37	24/12
sex (M/F), n	4/23	24/19	35/2	7/29
Age, yr (sd)	66.1 (12.6)	81.5 (12.1)	89.1 (8.1)	70 (32–87)
Stage (n) ^a^	IA (10)/IB (17)	IA (2), IB (16), IIA (4)	IA (3), IB (16), IIA (4)	I (14), II (12)
		IIB (4), IIIa (12), IIIb (4)	IIB (7), IIIA (4), IIIB (0)	III (8)
		IV(1)	IV(3)	IV (1)
Differentiation (n)	Well (20)	NA	NA	Well (7), Well/Mod (1)
	Moderate (7)	NA	NA	Mod (15), Mod/poor (5)
		NA	NA	poor (9)
Sequential selection	26	19	19	11/10
of control cases (n)				
Sequential selection	26	19	19	11/10
of cancer cases (n)				
Platform	GPL111154	GPL20301		GPL17553
	Illumina	Illumina		Illumina
	HiSeq 2000	HiSeq 4 000		HiSeq 2000
Reference	[21]	[22]		[23]

^a^ Stage of one case is unidentified.

**Table 2:** Network parameters, including density, heterogeneity, centralization, distribution degree, and modularity.

	GSE87340		GSE120622			GSE111907-1				GSE111907-2			
	N	LUAD	N	LUAD	LUSC	CD45^+^	CD10^+^	CD31^+^	LUAD	CD45^+^	CD10^+^	CD31^+^	LUSC
Nodes (N)	155	79	385	228	156	385	150	118	158	400	159	151	116
Edge (N)	805	109	2704	1069	319	4839	670	1579	472	4820	1149	517	327
Average neighbor	14.07	3.49	19.81	11.544	10	30.369	12	39.128	10.8	29.11	22.813	18.857	6.069
Network diameter	9	14	22	10	5	11	6	9	5	12	8	6	5
Network radius	5	7	11	5	3	6	3	5	3	6	4	3	3
Path length	2.852	4.994	6.463	3.765	2.2	3.167	2.177	2.11	2.032	4.148	2.393	1.782	2.365
Clustering coefficient	0.552	0.358	0.544	0.545	0.762	0.554	0.627	0.801	0.614	0.523	0.761	0.89	0.513
Network density	0.13	0.07	0.078	0.064	0.325	0.097	0.444	0.508	0.372	0.090	0.362	0.555	0.217
Hetero- geneity	0.834	0.682	1.097	0.766	0.507	0.788	0.612	0.564	0.604	0.803	0.678	0.518	0.59
Network centrality	0.273	0.136	0.216	0.121	0.273	0.182	0.279	0.225	0.34	0.174	0.282	0.192	0.304
Connected components	15	11	32	22	30	28	25	12	36	25	21	39	21
Assortativity	0.351	0.512	0.605	0.375	0.858	0.422	0.894	0.873	0.791	0.529	0.781	0.931	0.833
Modularity	0.293	0.677	0.333	0.571	0.765	0.383	0.778	0.107	0.7246	0.502	0.559	0.549	0.751

Abbreviations: N, normal; LUAD, lung adenocarcinoma; LUSC, squamous cell lung carcinoma; CD45^+^, immune cell; CD10^+^, fibroblast; CD31^+^, endothelial cell.

## Data Availability

Data are contained within the article and Supplementary Materials.

## Supplementary Materials

The following supporting information can be downloaded at: https://www.mdpi.com/article/10.3390/biomedicines12030628/s1, Figure S1: GSE87340 dataset gene set enrichment for pathway analysis for normal tissue and LUAD; Figure S2: GSE120622 dataset gene set enrichment for pathway analysis for normal tissue compared with LUAD; Figure S3: GSE120622 dataset gene set enrichment for pathway analysis for normal tissue compared with LUSC; Figure S4: GSE111907-1 dataset gene set enrichment for pathway analysis for normal tissue compared with LUAD; Figure S5: GSE111907-2 dataset gene set enrichment for pathway analysis for normal tissue compared with LUSC; Figure S6: Constructed network of GSE1119707-1 sorted fibroblast and endothelial cell from LUAD; Figure S7: Constructed network of GSE1119707-2 sorted fibroblast and endothelial cell from LUSC; Figure S8: Proportion of annotated gene function within the network. GSE87340 and GSE1290622. Normal (blue), LUAD (red), and LUSC (green) are plotted; Figure S9: Proportion of annotated gene function within the network. GSE111907-1 and GSE111907-2. Normal (blue), LUAD (red), and LUSC (green) are plotted; Figure S10: Connectivity map analysis for network gene expression signatures; Table S1: Summary of clinical study of immune checkpoint inhibitor for non-squamous non-small-cell lung cancer and advanced squamous cell lung cancer (LUSC); Table S2: Downloaded SRA raw data for GSE120622; Table S3: Downloaded SRA raw data for GSE111907; Table S4: Software along with version and reference genome for alignment of RNA reads; Table S5: Gene set enrichment for pathway analysis based on KEGG database; Table S6: Number of genes with annotated gene function in the constructed networks; Table S7: Connectivity analysis for GSE87340 LUAD; Table S8. Connectivity map analysis for GSE120622 LUAD; Table S9: Connectivity map analysis for GSE120622 LUSC; Table S10: Connectivity map analysis of GSE111907 LUAD; Table S11: Connectivity map analysis for GSE111907 LUSC.
