# Supplementary material for: Immune Gene Networks from Lung Cancer Patients Treated with Immune Checkpoint Inhibitors
Source: Biomedicines. 2024 Mar 12;12(3):628. doi: 10.3390/biomedicines12030628 (PMC10968332; doi:10.3390/biomedicines12030628)

Supplementary Table S1. Summary of clinical study of immune checkpoint inhibitor for non squamous, non small cell lung cancer and advanced squamous cell lung cancer (LUSC).

	Nonsquamous Non-small-cell Lung Cancer	LUSC
Reference	[8]	[9]
Median OS (month)		
Nivolumab	12.2	9.2
Docetaxel	9.4	6
HR	0.73	0.59
1yr OS (%)		
Nivolumab	0.51	0.42
Docetaxel	0.39	0.24
1yr PFS (%)		
Nivolumab	0.19	0.21
Docetaxel	0.12	0.06
1yr PFS (month)		
Nivolumab	19	NA
Docetaxel	8	NA
HR	0.92	NA
Median PFS		
Nivolumab	2.3	3.5
Docetaxel	4.2	2.8
HR	**0.92**	0.62
Duration of Response		
Nivolumab	0.52	0.63
Docetaxel	0.14	0.33

OS, overall survival; yr, year; HR, hazard ratio; PFS, progression free survival; NA, not available

Supplementary Table S2. Downloaded SRA raw data for GSE120622.

Run	GEO_Accession	source_name	Histology
SRR7939779	GSM3405969	lung cancer	LUAD
SRR7939780	GSM3405970	lung cancer	LUAD
SRR7939781	GSM3405971	lung cancer	LUAD
SRR7939783	GSM3405973	lung cancer	LUAD
SRR7939784	GSM3405974	lung cancer	LUAD
SRR7939785	GSM3405975	lung cancer	LUAD
SRR7939786	GSM3405976	lung cancer	LUAD
SRR7939787	GSM3405977	lung cancer	LUAD
SRR7939788	GSM3405978	lung cancer	LUAD
SRR7939789	GSM3405979	lung cancer	LUAD
SRR7939790	GSM3405980	lung cancer	LUAD
SRR7939791	GSM3405981	lung cancer	LUAD
SRR7939792	GSM3405982	lung cancer	LUAD
SRR7939793	GSM3405983	lung cancer	LUAD
SRR7939794	GSM3405984	lung cancer	LUAD
SRR7939795	GSM3405985	lung cancer	LUAD
SRR7939796	GSM3405986	lung cancer	LUAD
SRR7939797	GSM3405987	lung cancer	LUAD
SRR7939798	GSM3405988	lung cancer	LUAD
SRR7939823	GSM3406013	lung cancer	LUSC
SRR7939824	GSM3406014	lung cancer	LUSC
SRR7939825	GSM3406015	lung cancer	LUSC
SRR7939830	GSM3406020	lung cancer	LUSC
SRR7939831	GSM3406021	lung cancer	LUSC
SRR7939834	GSM3406024	lung cancer	LUSC
SRR7939835	GSM3406025	lung cancer	LUSC
SRR7939838	GSM3406028	lung cancer	LUSC
SRR7939839	GSM3406029	lung cancer	LUSC
SRR7939840	GSM3406030	lung cancer	LUSC
SRR7939841	GSM3406031	lung cancer	LUSC
SRR7939842	GSM3406032	lung cancer	LUSC
SRR7939843	GSM3406033	lung cancer	LUSC
SRR7939844	GSM3406034	lung cancer	LUSC
SRR7939845	GSM3406035	lung cancer	LUSC
SRR7939846	GSM3406036	lung cancer	LUSC
SRR7939847	GSM3406037	lung cancer	LUSC
SRR7939848	GSM3406038	lung cancer	LUSC
SRR7939849	GSM3406039	lung cancer	LUSC
SRR7939860	GSM3406050	adjacent lung	normal
SRR7939861	GSM3406051	adjacent lung	normal
SRR7939862	GSM3406052	adjacent lung	normal
SRR7939863	GSM3406053	adjacent lung	normal
SRR7939864	GSM3406054	adjacent lung	normal
SRR7939865	GSM3406055	adjacent lung	normal
SRR7939866	GSM3406056	adjacent lung	normal
SRR7939867	GSM3406057	adjacent lung	normal
SRR7939868	GSM3406058	adjacent lung	normal
SRR7939869	GSM3406059	adjacent lung	normal
SRR7939870	GSM3406060	adjacent lung	normal

SRR7939871	GSM3406061	adjacent lung	normal
SRR7939872	GSM3406062	adjacent lung	normal
SRR7939873	GSM3406063	adjacent lung	normal
SRR7939874	GSM3406064	adjacent lung	normal
SRR7939875	GSM3406065	adjacent lung	normal
SRR7939876	GSM3406066	adjacent lung	normal
SRR7939877	GSM3406067	adjacent lung	normal
SRR7939878	GSM3406068	adjacent lung	normal

Supplementary Table S3. Downloaded SRA raw data for GSE111907.

Run	Sample	BioSample	SampleName	PT.num	histology	PT.cell
SRR11491791	SRS6436479	SAMN08719584	GSM3044677	T1	adeno	Malignant re
SRR11491792	SRS6436480	SAMN08719583	GSM3044678	T1	adeno	Fibroblast
SRR11491793	SRS6436481	SAMN08719582	GSM3044679	T1	adeno	Endothelial
SRR11491794	SRS6436482	SAMN08719581	GSM3044680	T1	adeno	Pan immune
SRR11491800	SRS6436488	SAMN08719575	GSM3044686	T3	sq	Malignant
SRR11491801	SRS6436489	SAMN08719574	GSM3044687	T3	sq	Fibroblast
SRR11491802	SRS6436490	SAMN08719573	GSM3044688	T3	sq	Endothelial
SRR11491803	SRS6436491	SAMN08719572	GSM3044689	T3	sq	Pan immune
SRR11491805	SRS6436493	SAMN08719570	GSM3044691	T4	sq	Malignant
SRR11491806	SRS6436494	SAMN08719569	GSM3044692	T4	sq	Fibroblast
SRR11491807	SRS6436495	SAMN08719568	GSM3044693	T4	sq	Endothelial
SRR11491808	SRS6436496	SAMN08719567	GSM3044694	T4	sq	Pan immune
SRR11491810	SRS6436498	SAMN08719565	GSM3044696	T5	adeno	Malignant
SRR11491811	SRS6436499	SAMN08719564	GSM3044697	T5	adeno	Fibroblast
SRR11491812	SRS6436500	SAMN08719563	GSM3044698	T5	adeno	Endothelial
SRR11491813	SRS6436501	SAMN08719677	GSM3044699	T5	adeno	Pan immune
SRR11491815	SRS6436503	SAMN08719675	GSM3044701	T6	adeno	Malignant
SRR11491816	SRS6436504	SAMN08719674	GSM3044702	T6	adeno	Fibroblast
SRR11491817	SRS6436505	SAMN08719673	GSM3044703	T6	adeno	Endothelial
SRR11491818	SRS6436506	SAMN08719672	GSM3044704	T6	adeno	Pan immune
SRR11491820	SRS6436508	SAMN08719670	GSM3044706	T7	adeno	Malignant
SRR11491821	SRS6436509	SAMN08719669	GSM3044707	T7	adeno	Fibroblast
SRR11491822	SRS6436510	SAMN08719668	GSM3044708	T7	adeno	Endothelial
SRR11491823	SRS6436511	SAMN08719667	GSM3044709	T7	adeno	Pan immune
SRR11491825	SRS6436513	SAMN08719665	GSM3044711	T8	adeno	Malignant
SRR11491826	SRS6436514	SAMN08719664	GSM3044712	T8	adeno	Fibroblast
SRR11491827	SRS6436515	SAMN08719663	GSM3044713	T8	adeno	Endothelial
SRR11491829	SRS6436517	SAMN08719729	GSM3044715	T8	adeno	Pan immune
SRR11491836	SRS6436524	SAMN08719722	GSM3044722	T10	adeno	Malignant re
SRR11491838	SRS6436526	SAMN08719720	GSM3044724	T10	adeno	Fibroblast
SRR11491839	SRS6436527	SAMN08719719	GSM3044725	T10	adeno	Endothelial
SRR11491840	SRS6436528	SAMN08719718	GSM3044726	T10	adeno	Pan immune
SRR11491843	SRS6436531	SAMN08719710	GSM3044729	T11	adeno	Malignant
SRR11491844	SRS6436532	SAMN08719717	GSM3044730	T11	adeno	Fibroblast

SRR11491845	SRS6436533	SAMN08719716	GSM3044731	T11	adeno	Endothelial
SRR11491846	SRS6436534	SAMN08719715	GSM3044732	T11	adeno	Pan immune
SRR11491848	SRS6436536	SAMN08719713	GSM3044734	T12	adeno	Malignant
SRR11491849	SRS6436537	SAMN08719709	GSM3044735	T12	adeno	Fibroblast
SRR11491850	SRS6436538	SAMN08719708	GSM3044736	T12	adeno	Endothelial
SRR11491851	SRS6436539	SAMN08719707	GSM3044737	T12	adeno	Pan immune
SRR11491853	SRS6436541	SAMN08719705	GSM3044739	T14	sq	Malignant
SRR11491855	SRS6436543	SAMN08719703	GSM3044741	T14	sq	Endothelial
SRR11491857	SRS6436545	SAMN08719701	GSM3044743	T14	sq	Panimmune
SRR11491859	SRS6436546	SAMN08719699	GSM3044745	T15	sq	Malignant
SRR11491860	SRS6436548	SAMN08719698	GSM3044746	T15	sq	Fibroblast
SRR11491861	SRS6436549	SAMN08719697	GSM3044747	T15	sq	Endothelial
SRR11491862	SRS6436550	SAMN08719740	GSM3044748	T15	sq	Pan immune
SRR11491864	SRS6436552	SAMN08719738	GSM3044750	T16	adeno	Malignant
SRR11491865	SRS6436553	SAMN08719737	GSM3044751	T16	adeno	Fibroblast
SRR11491866	SRS6436554	SAMN08719736	GSM3044752	T16	adeno	Endothelial
SRR11491867	SRS6436555	SAMN08719735	GSM3044753	T16	adeno	Pan immune
SRR11491868	SRS6436557	SAMN08719734	GSM3044754	T17	sq	Malignant
SRR11491869	SRS6436556	SAMN08719733	GSM3044755	T17	sq	Fibroblast
SRR11491870	SRS6436558	SAMN08719730	GSM3044756	T17	sq	Endothelial
SRR11491871	SRS6436559	SAMN08719638	GSM3044757	T17	sq	Pan immune
SRR11491874	SRS6436562	SAMN08719732	GSM3044760	T18	sq	Malignant
SRR11491875	SRS6436563	SAMN08719731	GSM3044761	T18	sq	Fibroblast
SRR11491876	SRS6436564	SAMN08719635	GSM3044762	T18	sq	Endothelial
SRR11491877	SRS6436565	SAMN08719634	GSM3044763	T18	sq	Pan immune
SRR11491879	SRS6436567	SAMN08719632	GSM3044765	T20	sq	Malignant
SRR11491880	SRS6436568	SAMN08719631	GSM3044766	T20	sq	Fibroblast
SRR11491881	SRS6436569	SAMN08719630	GSM3044767	T20	sq	Endothelial
SRR11491882	SRS6436570	SAMN08719629	GSM3044768	T20	sq	Pan immune
SRR11491884	SRS6436572	SAMN08719696	GSM3044770	T22	adeno	Malignant
SRR11491885	SRS6436573	SAMN08719695	GSM3044771	T22	adeno	Fibroblast
SRR11491886	SRS6436574	SAMN08719694	GSM3044772	T22	adeno	Endothelial
SRR11491887	SRS6436576	SAMN08719693	GSM3044773	T22	adeno	Pan immune
SRR11491892	SRS6436396	SAMN08719688	GSM3044778	T25	sq	Malignant
SRR11491893	SRS6436397	SAMN08719687	GSM3044779	T25	sq	Fibroblast
SRR11491894	SRS6436398	SAMN08719686	GSM3044780	T25	sq	Endothelial
SRR11491896	SRS6436400	SAMN08719684	GSM3044782	T25	sq	Pan immune

SRR11491897	SRS6436401	SAMN08719683	GSM3044783	T26	adeno	Malignant
SRR11491898	SRS6436402	SAMN08719682	GSM3044784	T26	adeno	Fibroblast
SRR11491899	SRS6436403	SAMN08719681	GSM3044785	T26	adeno	Endothelial
SRR11491900	SRS6436404	SAMN08719680	GSM3044786	T26	adeno	Pan immune
SRR11491902	SRS6436406	SAMN08719678	GSM3044788	T27	adeno	Malignant
SRR11491903	SRS6436407	SAMN08719598	GSM3044789	T27	adeno	Fibroblast
SRR11491904	SRS6436408	SAMN08719597	GSM3044790	T27	adeno	Endothelial
SRR11491905	SRS6436409	SAMN08719596	GSM3044791	T27	adeno	Pan immune
SRR11491928	SRS6436432	SAMN08719643	GSM3044814	T32	sq	Malignant
SRR11491929	SRS6436433	SAMN08719642	GSM3044815	T32	sq	Endothelial
SRR11491930	SRS6436434	SAMN08719639	GSM3044816	T32	sq	Pan immune
SRR11491935	SRS6436439	SAMN08719640	GSM3044821	T34	sq	Fibroblast
SRR11491936	SRS6436440	SAMN08719559	GSM3044822	T34	sq	Endothelial
SRR11491937	SRS6436441	SAMN08719558	GSM3044823	T34	sq	Pan immune
SRR11491938	SRS6436442	SAMN08719557	GSM3044824	T34	sq	Malignant
SRR11491782	SRS6436464	SAMN08719607	GSM3044846	T39	sq	Fibroblast
SRR11491783	SRS6436465	SAMN08719606	GSM3044847	T39	sq	Endothelial
SRR11491784	SRS6436466	SAMN08719605	GSM3044848	T39	sq	Pan immune
SRR11491785	SRS6436467	SAMN08719604	GSM3044849	T39	sq	Malignant
SRR11491787	SRS6436469	SAMN08719602	GSM3044851	T40	sq	Fibroblast
SRR11491788	SRS6436470	SAMN08719601	GSM3044852	T40	sq	Endothelial
SRR11491789	SRS6436471	SAMN08719600	GSM3044853	T40	sq	Pan immune
SRR11491790	SRS6436472	SAMN08719599	GSM3044854	T40	sq	Malignant

PT, patient; adeno, adenocarcinoma; sq, squamous cell lung carcinoma

Supplementary Table S4. Softwares along with version and reference genome for alignment of RNA reads

Softwares and versions / reference genome
Ubuntu 22.04 LTS
Sratoolkit-3.0.10
HISAT2-2.2.1
Samtools-1.19
Stringtie-2.2.1
Reference genome: GRCH38
R version 4.3
DESeq2-1.42.0 (R package)
Hmisc-5.1-1 (R package)
ggplot2 3.5.0 (R package)
Cytoscpate network analyzer 4.5.0
igraph 2.0.2 (R package)

Supplementary Table S5. Gene set enrichment for pathway analysis based on KEGG database

	GSE87340	GSE120622		GSE111907-1	GSE111907-2
	LUAD	LUAD	LUSC	LUAD	LUSC
Control	27	19	19	10	11
Tumor	26	19	19	10	11
Up regulation	23	20	31	32	32
Down regulation	25	3	6	6	7
Up regulated immune pathway	NOD	Fc, BCR, Toll,RIG,TCR	Fc, BCR, TCR,Toll,RIG	Fc, NOD,TCR, Toll, RIG	Fc, TCR,NOD, RIG,K
Down regulated immune pathway	B, C, K, H, LM, NK, Fc, T	H	H,C,IgA,LM	H,C,IgA	H,C,IgA

LUAD, adenocarcinoma non-small-cell lung cancer; LUSC, squamous non-small-cell lung carcinoma; APC, antigen processing and presentation; BCR, B cell receptor signaling pathway; C, complement and coagulation pathway; Fc, Fc gamma receptor mediated phagocytosis; H, hematopoietic cell lineage; IgA, intestinal immune network for IgA production; K, cytokine-chemokine signaling pathway; LM, leukocyte migration; NK, NK cell mediated cytotoxicity; NOD-like receptor signaling pathway, RIG, RIG-I like receptor signaling; Toll, Toll-like receptor signaling pathway; TCR, T cell receptor signaling pathway,

Supplementary Table S6. Number of gene with Annotated gene function in the constructed networks

Dataset	sample	Gene Function										
		A	APC	CP	H	I	K	LM	NK	P	S	Total
GSE87340	Normal	54	0	1	9	39	17	3	15	5	12	155
	Rate	0.35	0.00	0.01	0.06	0.25	0.11	0.02	0.10	0.03	0.08	1
	LUAD	34	0	2	7	13	10	2	7	1	3	79
	Rate	0.43	0.00	0.03	0.09	0.16	0.13	0.03	0.09	0.01	0.04	1
GSE120622	Normal	101	2	16	16	103	36	26	26	23	36	385
	Rate	0.26	0.01	0.04	0.04	0.27	0.09	0.07	0.07	0.06	0.09	1.00
	LUAD	63	0	18	19	43	26	16	19	14	10	228
	Rate	0.28	0.00	0.08	0.08	0.19	0.11	0.07	0.08	0.06	0.04	1.00
	LUSC	50	2	9	12	30	15	12	15	8	3	156
	Rate	0.32	0.01	0.06	0.08	0.19	0.10	0.08	0.10	0.05	0.02	1.00
GSE111907-1	Normal	98	2	10	12	114	30	17	39	22	41	385
	Rate	0.25	0.01	0.03	0.03	0.30	0.08	0.04	0.10	0.06	0.11	1.00
	LUAD	32	0	9	8	49	18	9	10	8	15	158
	Rate	0.20	0.00	0.06	0.05	0.31	0.11	0.06	0.06	0.05	0.09	1.00
GSE111907-2	Normal	103	2	11	12	116	31	20	41	23	41	400
	Rate	0.26	0.01	0.03	0.03	0.29	0.08	0.05	0.10	0.06	0.10	1.00
	LUSC	21	0	13	7	26	12	10	13	8	6	116
	Rate	0.18	0.00	0.11	0.06	0.22	0.10	0.09	0.11	0.07	0.05	1.00

LUAD, adenocarcinoma non-small-cell lung cancer; LUSC, squamous non-small-cell lung carcinoma; A, daptive immunity; APC, antigen presentation; CP, coagulation; H, hematopoiesis; I, innate immunity; K, cytokine and chemokine; LM, leukocyte migration; NK, NK cell; P, platelet; S, signaling

Supplementary Table S7. Connectivity analysis for GSE87340 LUAD

Name	Type	Perturbagen Id	Target	MOA
CGP-57380	trt_cp	BRD-K42500029	AURKB, LCK, MKNK1, MKNK2, SGK1	MAP kinase inhibitor
chloroquine	trt_cp	BRD-A91699651	CYP2C8, GSTA2, MRGPRX1, TLR9, TNF	Antimalarial
dorsomorphin	trt_cp	BRD-K54233340	ACVR1, BMPR1A, BMPR1B, EPHA2, FKBP1A, FLT1, FLT3, KDR, LCK, MKNK1, PRKAA1, RPS6KA1, SRC	AMPK inhibitor
indoprofen	trt_cp	BRD-A44090213	PTGS1, PTGS2, CXCR1, CXCR2	Cyclooxygenase inhibitor, Prostanoid receptor antagonist
indirubin	trt_cp	BRD-K53959060	CDK1, CDK5, CCNE1, CDK2, CDK4, CDK5R1, CDK9, GSK3A, GSK3B, LCK, LRRK1, LRRK2	CDK inhibitor, Glycogen synthase kinase inhibitor, Leucine rich repeat kinase inhibitor, SRC inhibitor, Serum/glucocorticoid regulated kinase inhibitor
indirubin	trt_cp	BRD-K19136521	CDK1, CDK5, CCNE1, CDK2, CDK4, CDK5R1, CDK9, GSK3A, GSK3B, LCK, LRRK1, LRRK2	CDK inhibitor
LY-294002	trt_cp	BRD-K27305650	MTOR, PIK3CD, PIK3CG, PIK3CA, PIK3CB, PLK1, PRKDC, AKT1, CHEK1, GSK3B, LCK, MAPK1, MAPK11, MAPK12, MAPK14, MAPK8, PDE2A, PRKCA, ROCK1, RPS6KB1, SGK1	MTOR inhibitor, PI3K inhibitor, DNA dependent protein kinase inhibitor, Phosphodiesterase inhibitor, PLK inhibitor
bisindolylmaleimide-ix	trt_cp	BRD-K06543683	SIRT1, AKT1, GSK3B, LCK, LRRK2, MAPK1, MAPK11, MAPK12, MAPK14, MAPK8, PRKCA, ROCK1, RPS6KB1, SIRT2	CDK inhibitor, PKC inhibitor
SRC-kinase-inhibitor-I	trt_cp	BRD-K50495309	CSK, LCK, RIPK2	SRC inhibitor
TCS-359	trt_cp	BRD-K81376179	FLT3	FLT3 inhibitor
wortmannin	trt_cp	BRD-A11678676	PIK3CA, PIK3CG, PLK1, ATM, ATR, MTOR, PI4KA, PI4KB, PIK3CD, PIK3R1, PLK3, PRKDC	PI3K inhibitor

ZK-756326	trt_cp	BRD-K56403959	CCR8	CC chemokine receptor ligand
ZM-39923	trt_cp	BRD-K40624912	JAK1, JAK3	JAK inhibitor
AG-490	trt_cp	BRD-K12357156	JAK2, JAK3, EGFR, STAT3	EGFR inhibitor, ErbB2 inhibitor, JAK inhibitor
tyrphostin-AG-82	trt_cp	BRD-K03670461	EGFR, LCK	EGFR inhibitor
tyrphostin-AG-1296	trt_cp	BRD-K76064317	FLT3	FLT3 inhibitor
aminogenistein	trt_cp	BRD-K04976539	LCK	SRC inhibitor
GTP-14564	trt_cp	BRD-K16664969	FLT3, CSF1R, KIT, PDGFRB	FLT3 inhibitor, Tyrosine kinase inhibitor
ivermectin	trt_cp	BRD-A48570745	CHRNA7, GABRB3, GLRA3, P2RX7	GABA receptor agonist
JAK3-inhibitor-I	trt_cp	BRD-K72541103	JAK3	JAK inhibitor
JAK3-Inhibitor-II	trt_cp	BRD-K52850071	EGFR, ALK, JAK1, JAK2, JAK3	JAK inhibitor
JAK3-inhibitor-V	trt_cp	BRD-K95676198	JAK3	JAK inhibitor
JAK3-inhibitor-VI	trt_cp	BRD-K04546108	JAK3	JAK inhibitor
ketoprofen	trt_cp	BRD-A97739905	PTGS2, PTGS1, CXCR1, SLC5A8	Cyclooxygenase inhibitor
KN-62	trt_cp	BRD-A81177136	AKT1, CAMK2A, CHEK1, LCK, MAPK1, MAPK11, MAPK12, MAPK14, MAPK8, P2RX7, PRKCA, ROCK1, RPS6KB1, SGK1	Calcium-calmodulin dependent protein kinase inhibitor, Purinergic receptor antagonist
kenpaullone	trt_cp	BRD-K37312348	GSK3B, CDK1, CDK5, CCNB1, CDK2, LCK	CDK inhibitor, Glycogen synthase kinase inhibitor
lavendustin-a	trt_cp	BRD-K23583188	EGFR, LCK	EGFR inhibitor
LFM-A13	trt_cp	BRD-A30655177	BTK	BTK inhibitor
leflunomide	trt_cp	BRD-K78692225	DHODH, AHR, CYP2C19, JAK3, PTK2B, STAT6	Dihydroorotate dehydrogenase inhibitor, PDGFR receptor inhibitor
PD-98059	trt_cp	BRD-K62810658	MAP2K1, AKT1, CHEK1, GSK3B, LCK, MAP2K2, MAPK1, MAPK11, MAPK12, MAPK14, MAPK3, MAPK8, PRKCA, RAF1, ROCK1, RPS6KB1, SGK1	MEK inhibitor, MAP kinase inhibitor
D-64406	trt_cp	BRD-K27665173	FLT3, PDGFRA, PDGFRB	PDGFR receptor inhibitor
midostaurin	trt_cp	BRD-K13646352	FLT3, KIT, CCNB1, FLT1, KDR, PDGFRB, PRKCA, PRKCG, VEGFA	FLT3 inhibitor, KIT inhibitor, PKC inhibitor
PP-2	trt_cp	BRD-K95785537	SRC, LCK, ABL1, LYN, RIPK2	SRC inhibitor
quercetin	trt_cp	BRD-K97399794	PIK3CG, AKR1B1, ATP5A1, ATP5B, ATP5C1, CYP2C8, EGFR, GAA, HCK, HIBCH, MAOA, PIM1, PTPN1, SCN5A, SIRT1, STK17B, UGT3A1, XDH	Polar auxin transport inhibitor

SB-202190	trt_cp	BRD-K54330070	MAPK14, AKT1, ALOX5, CHEK1, GSK3B, LCK, MAPK1, MAPK11, MAPK12, MAPK8, PRKCA, ROCK1, RPS6KB1, SGK1	p38 MAPK inhibitor
SB-225002	trt_cp	BRD-K61323504	CXCR2	CC chemokine receptor antagonist
semaxanib	trt_cp	BRD-K63504947	KDR, FLT1, KIT, PDGFRB, FGFR1, FLT3, MET, PDGFRA, RET	VEGFR inhibitor
SU-6656	trt_cp	BRD-K97354755	LYN, AURKB, AURKC, BRSK2, CALM1, CAMK2D, LCK, RET, SRC, YES1	SRC inhibitor
4,5,6,7-tetrabromo benzotriazole	trt_cp	BRD-K97118047	CSNK2A1, AKT1, CHEK1, CSNK2A2, CSNK2B, GSK3B, LCK, MAP2K1, MAPK1, MAPK11, MAPK12, MAPK14, MAPK8, PRKCA, ROCK1, RPS6KB1, SGK1	Casein kinase inhibitor
U-0126	trt_cp	BRD-K18787491	AKT1, CHEK1, GSK3B, JAK2, LCK, MAP2K1, MAP2K2, MAP2K7, MAPK1, MAPK11, MAPK12, MAPK14, MAPK8, PRKCA, RAF1, ROCK1, RPS6KB1, SGK1	MEK inhibitor
dactolisib	trt_cp	BRD-K12184916	MTOR, PIK3CA, PIK3CG, PIK3CD, ATR, PIK3CB	MTOR inhibitor, PI3K inhibitor, Protein kinase inhibitor
AS-605240	trt_cp	BRD-K41895714	MAOB, PIK3CA, PIK3CB, PIK3CD, PIK3CG	PI3K inhibitor
BMS-536924	trt_cp	BRD-K34581968	IGF1R, AKT1, CCNE1, CDK2, CYP3A4, ERBB2, INSR, KDR, LCK, MAPK1, MET, PDGFRA, PDGFRB	IGF-1 inhibitor
KU-0060648	trt_cp	BRD-K09499853	PIK3CA, PIK3CB, PIK3CD, PIK3CG, PRKDC	DNA dependent protein kinase inhibitor, PI3K inhibitor
PI-103	trt_cp	BRD-K67868012	PIK3CA, PIK3CG, MTOR, PIK3CB, PIK3CD, PRKDC	MTOR inhibitor, PI3K inhibitor
fostamatinib	trt_cp	BRD-K20285085	SYK, FLT3, RET	SYK inhibitor
TG-101348	trt_cp	BRD-K12502280	JAK2, FLT3, BRD4, JAK1, JAK3, RET, TYK2	FLT3 inhibitor, JAK inhibitor
myricetin	trt_cp	BRD-K43149758	PIK3CG, AR, CYP3A4	Androgen receptor agonist, Cytochrome P450 inhibitor
HG-6-64-01	trt_cp	BRD-U37049823	ABL1, BRAF, CSF1R, EGFR, FGFR1, FLT3, KIT, MAPK11, PDGFRB, RET	RAF inhibitor
JW-7-24-1	trt_cp	BRD-U68942961	LCK	LCK Inhibitor
NM-PP1	trt_cp	BRD-K78278890	CAMK2A, LCK, MAPK8, PRKACA, RIPK2, SRC	Mutant kinase inhibitor
tozasertib	trt_cp	BRD-K59369769	AURKA, AURKB, ABL1, AURKC, BCR, FLT3, JAK2, DDR2, LCK	Aurora kinase inhibitor, BCR-ABL kinase

				inhibitor, FLT3 inhibitor, JAK inhibitor
dasatinib	trt_cp	BRD-K49328571	ABL1, FYN, LCK, SRC, KIT, YES1, BCR, EPHA2, LYN, PDGFRB, ABL2, BTK, DDR1, DDR2, PDGFRA, STAT5B	BCR-ABL kinase inhibitor, Ephrin inhibitor, KIT inhibitor, PDGFR receptor inhibitor, SRC inhibitor, Tyrosine kinase inhibitor
sorafenib	trt_cp	BRD-K49810818	RET, BRAF, FLT3, KDR, RAF1, FLT1, FLT4, KIT, DDR2, FGFR1, PDGFRB, CYP2B6, CYP2C8, CYP3A5, PDGFB, SLCO1B3	FLT3 inhibitor, KIT inhibitor, PDGFR receptor inhibitor, RAF inhibitor, VEGFR inhibitor, RET tyrosine kinase inhibitor
saracatinib	trt_cp	BRD-K19540840	SRC, ABL1, LCK, YES1	SRC inhibitor
tandutinib	trt_cp	BRD-K89162000	FLT3, KIT, PDGFRA, PDGFRB, CSF1R, PDGFD	FLT3 inhibitor, KIT inhibitor, PDGFR receptor inhibitor
ZSTK-474	trt_cp	BRD-K63068307	PIK3CG, PIK3CA, PIK3CB, PIK3CD	PI3K inhibitor
TG100-115	trt_cp	BRD-K64785675	PIK3CG, PIK3CA, PIK3CB, PIK3CD	-666
tofacitinib	trt_cp	BRD-K31283835	JAK3, JAK1, JAK2, CYP2C19, TYK2	JAK inhibitor
GDC-0941	trt_cp	BRD-K52911425	PIK3CG, PIK3CA, PIK3CB, PIK3CD	PI3K inhibitor
QL-X-138	trt_cp	BRD-U33728988	BTK, JAK3, MKNK2, MTOR, PRKDC	MTOR inhibitor
WH-4023	trt_cp	BRD-U44618005	ABL1, LCK, SRC	SRC inhibitor
QL-XII-47	trt_cp	BRD-U86922168	BMX, BTK	BTK inhibitor, Cytoplasmic tyrosine protein kinase BMX inhibitor
AS-604850	trt_cp	BRD-K63915849	PIK3CG, PIK3CA	PI3K inhibitor
tyrphostin-AG-1295	trt_cp	BRD-K57926513	FLT3, KDR, PDGFRA, PDGFRB	PDGFR receptor inhibitor
ruxolitinib	trt_cp	BRD-K53972329	JAK1, JAK2, TYK2, JAK3	JAK inhibitor
dexketoprofen	trt_cp	BRD-K43764301	CXCR1, PTGS1, PTGS2, SLC5A8	Cyclooxygenase inhibitor
SB-203580	trt_cp	BRD-K99291625	MAPK14, MAPK1, MAPK11, AKT1, ALOX5, CHEK1, CYP2D6, CYP3A4, GAK, GSK3B, LCK, MAPK10, MAPK12, MAPK8, MAPK9, PRKCA, RAF1, RIPK2, ROCK1, RPS6KB1, SGK1, SRC, TNF	p38 MAPK inhibitor
terreic-acid	trt_cp	BRD-A64228451	BTK	BTK inhibitor
AZD-6482	trt_cp	BRD-K58772419	PIK3CD, PIK3CG, PIK3CA, PIK3CB	PI3K inhibitor
KIN001-055	trt_cp	BRD-K68407802	EGFR, JAK3	EGFR inhibitor, JAK inhibitor, Leukotriene inhibitor, Mediator release inhibitor

quizartinib	trt_cp	BRD-K93918653	FLT3, CSF1R, KIT, RET, PDGFRA, PDGFRB	FLT3 inhibitor
lestaurtinib	trt_cp	BRD-K23192422	FLT3, NTRK1, JAK2, NTRK2, NTRK3	FLT3 inhibitor, Growth factor receptor inhibitor, JAK inhibitor
linifanib	trt_cp	BRD-K99749624	CSF1R, KDR, PDGFRB, FLT1, FLT3, FLT4, CSF1, KIT, PDGFRA, RET, TEK	PDGFR receptor inhibitor, VEGFR inhibitor
cediranib	trt_cp	BRD-K86930074	KDR, FLT1, FLT4, KIT, PDGFRB, CSF1R, FLT3, PDGFRA	KIT inhibitor, VEGFR inhibitor
dovitinib	trt_cp	BRD-K85402309	EGFR, FGFR3, PDGFRB, CSF1R, FGFR1, FGFR2, FLT1, FLT3, FLT4, INSR, KDR, KIT, PDGFRA	EGFR inhibitor, FLT3 inhibitor, FGFR inhibitor, PDGFR receptor inhibitor, VEGFR inhibitor
sunitinib	trt_cp	BRD-M64432851	FLT3, KDR, KIT, FLT4, FLT1, PDGFRA, PDGFRB, RET, CSF1R, FGFR1	FLT3 inhibitor, KIT inhibitor, PDGFR receptor inhibitor, RET tyrosine kinase inhibitor, VEGFR inhibitor
XL-147	trt_cp	BRD-K95901403	PIK3CA, PIK3CD, PIK3CG	PI3K inhibitor
PIK-75	trt_cp	BRD-M16762496	PIK3CA, PIK3CB, PIK3CD, PIK3CG, PRKDC	DNA protein kinase inhibitor, PI3K inhibitor
sunitinib	trt_cp	BRD-K70511574	FLT3, KDR, KIT, FLT4, FLT1, PDGFRA, PDGFRB, RET, CSF1R, FGFR1	PLK inhibitor
GSK-1059615	trt_cp	BRD-K06750613	PIK3CA, PIK3CG	PI3K inhibitor
staurosporine	trt_cp	BRD-K17953061	CDK2, GSK3B, CAMK2B, CDK1, CDK5, CHEK1, CHRM1, CHRM2, CHRM4, CSK, DAPK1, GPR35, IKBKB, ITK, LCK, LRRK2, MAP2K4, MAP2K6, MAPKAPK2, PAK2, PDPK1, PHKG2, PIK3CG, PIM1, PKN1, PRKACB, PRKCI, PRKCQ, RPS6KA1, STK3, SYK, TNIK, ZAP70	PKC inhibitor
CD19	trt_oe	ccsbBroad304_00250		
CXCR2	trt_oe	ccsbBroad304_00856		
IL21R	trt_oe	ccsbBroad304_03148		
LCK	trt_oe	ccsbBroad304_13891		
PIK3CG	trt_oe	ccsbBroad304_06727		
BTK	trt_oe	ccsbBroad304_00180		
CSF3	trt_oe	ccsbBroad304_10489		
CTLA4	trt_oe	ccsbBroad304_00392		
CSF3	trt_sh.cgs	CGS001-1440		
CTLA4	trt_sh.cgs	CGS001-1493		
FLT3	trt_sh.cgs	CGS001-2322		
CXCR2	trt_sh.cgs	CGS001-3579		

JAK3	trt_sh.cgs	CGS001-3718		
LCK	trt_sh.cgs	CGS001-3932		
PIK3CG	trt_sh.cgs	CGS001-5294		
TLR9	trt_sh.cgs	CGS001-54106		
NOD2	trt_sh.cgs	CGS001-64127		
BTK	trt_sh.cgs	CGS001-695		
CD19	trt_sh.cgs	CGS001-930		
AIM2	trt_sh.cgs	CGS001-9447		
AT-9283	trt_cp	BRD-K24576554	AURKA, AURKB, ABL1, BCR, FLT3, JAK2, JAK3, RPS6KA6, STK17A	JAK inhibitor, Aurora kinase inhibitor, ABL inhibitor, BCR-ABL kinase inhibitor, FLT3 inhibitor, Mitotic inhibitor, Protein kinase inhibitor
ENMD-2076	trt_cp	BRD-K68488863	AURKA, FLT3, KDR, PDGFRA, SRC, CSF1R, EPHA1, FGFR1, FGFR2, FGFR3, FLT4, KIT, PTK2	FLT3 inhibitor, VEGFR inhibitor, Aurora kinase inhibitor

Supplementary Table S8. Connectivity map analysis for GSE120622 LUAD

Name	Type	Perturbagen Id	Target	MOA
BW-B70C	trt_cp	BRD-A55946879	ALOX5	Lipoxygenase inhibitor
colforsin	trt_cp	BRD-A55416093	ADCY2, ADCY5, GNAS	Adenylyl cyclase activator, Adenylate cyclase stimulant
diclofenac	trt_cp	BRD-K08252256	PTGS1, PTGS2, AKR1C3, ALOX5, ASIC1, ASIC3, CYP2C8, KCNQ2, KCNQ3, PLA2G2A, PPARG, SCN4A	Cyclooxygenase inhibitor
diethylcarbamazine	trt_cp	BRD-K45542189	ALOX5, PTGS1	Lipoxygenase inhibitor
indoprofen	trt_cp	BRD-A44090213	PTGS1, PTGS2, CXCR1, CXCR2	Cyclooxygenase inhibitor, Prostanoid receptor antagonist
menadione	trt_cp	BRD-K78126613	AOX1, BGLAP, F10, F2, F7, F9, GGCX, NQO1, NQO2, PKM, PROC, PROS1, PROZ, VKORC1, VKORC1L1	Mitochondrial DNA polymerase inhibitor, Phosphatase inhibitor
rhamnetin	trt_cp	BRD-K37206356	ALOX5, MAPK8	HDAC inhibitor
triciribine	trt_cp	BRD-K80431395	AKT1, AKT2, AKT3	AKT inhibitor
rutin	trt_cp	BRD-K20482099	AKR1C3, AKR1B1, F10	Antioxidant, Capillary stabilizing agent, Nitric oxide scavenger
CDC	trt_cp	BRD-K10870738	ALOX12, ALOX5	Lipoxygenase inhibitor
caffeic-acid	trt_cp	BRD-K84709232	ALOX5, MIF, RELA, TNF	Lipoxygenase inhibitor, HIV integrase inhibitor, NFkB pathway inhibitor, Nitric oxide production inhibitor, PPAR receptor modulator, TNF production inhibitor, Tumor necrosis factor production inhibitor

dextromethorphan	trt_cp	BRD-K33211335	SIGMAR1, CHRNA2, CHRNA3, CHRNA4, CHRNA7, CHRNB2, CHRNB4, CYBA, CYBB, CYP3A5, GRIN1, GRIN3A, NCF1, NCF2, NCF4, OPRD1, OPRK1, OPRM1, PGRMC1, RAC1, RAC2, SLC6A2, SLC6A4	Glutamate receptor antagonist, Sigma receptor agonist
GTP-14564	trt_cp	BRD-K16664969	FLT3, CSF1R, KIT, PDGFRB	FLT3 inhibitor, Tyrosine kinase inhibitor
LFM-A13	trt_cp	BRD-A30655177	BTK	BTK inhibitor
meclofenamic-acid	trt_cp	BRD-K50398167	PTGS1, PTGS2, ALOX5, CNR1, KCNQ2, KCNQ3	Cyclooxygenase inhibitor, Prostanoid receptor antagonist
PD-169316	trt_cp	BRD-K77133231	ALOX5	p38 MAPK inhibitor
REV-5901	trt_cp	BRD-A68281735	ALOX5	Leukotriene receptor antagonist, Lipoxygenase inhibitor
SB-202190	trt_cp	BRD-K54330070	MAPK14, AKT1, ALOX5, CHEK1, GSK3B, LCK, MAPK1, MAPK11, MAPK12, MAPK8, PRKCA, ROCK1, RPS6KB1, SGK1	p38 MAPK inhibitor
SB-225002	trt_cp	BRD-K61323504	CXCR2	CC chemokine receptor antagonist
SKF-86002	trt_cp	BRD-K96809896	ALOX5, MAPK14	p38 MAPK inhibitor
tenidap	trt_cp	BRD-A87479750	ALOX5, KCNJ4, PTGS1	Cyclooxygenase inhibitor
zileuton	trt_cp	BRD-A56359832	ALOX5	Leukotriene inhibitor, Lipoxygenase inhibitor
AKT-inhibitor-1-2	trt_cp	BRD-K04887706	AKT1, AKT2, AKT3	AKT inhibitor
maraviroc	trt_cp	BRD-A04352665	CCR5, CYP3A5	CC chemokine receptor antagonist
MK-2206	trt_cp	BRD-K68065987	AKT1, AKT2, AKT3	AKT inhibitor
quinine	trt_cp	BRD-U94846492	KCNN4, ABCB1, CYP2D6, GP9, KCNB2, SLC29A4	Hemozoin biocrystallization inhibitor
forskolin	trt_cp	BRD-A70449690	ADCY2, ADCY5, GNAS	Adenylyl cyclase activator
phorbol-12-myristate-13-acetate	trt_cp	BRD-A15079084	CD4, KCNT2, PRKCA, TRPV4	PKC activator

HG-6-64-01	trt_cp	BRD-U37049823	ABL1, BRAF, CSF1R, EGFR, FGFR1, FLT3, KIT, MAPK11, PDGFRB, RET	RAF inhibitor
mesalazine	trt_cp	BRD-K28849549	PTGS1, PPARG, PTGS2, ALOX5, CHUK, CTNNB1, IKBKB, MPO, NAT1	Cyclooxygenase inhibitor, Lipoxygenase inhibitor, Prostanoid receptor antagonist
sirolimus	trt_cp	BRD-K89626439	MTOR, FKBP1A, CCR5, FGF2	MTOR inhibitor
dasatinib	trt_cp	BRD-K49328571	ABL1, FYN, LCK, SRC, KIT, YES1, BCR, EPHA2, LYN, PDGFRB, ABL2, BTK, DDR1, DDR2, PDGFRA, STAT5B	BCR-ABL kinase inhibitor, Ephrin inhibitor, KIT inhibitor, PDGFR receptor inhibitor, SRC inhibitor, Tyrosine kinase inhibitor
imatinib	trt_cp	BRD-K92723993	ABL1, KIT, PDGFRA, BCR, CSF1R, PDGFRB, ABCG2, CYP2C19, CYP2C8, CYP3A5, DDR1, NTRK1, RET	BCR-ABL kinase inhibitor, KIT inhibitor, PDGFR receptor inhibitor
axitinib	trt_cp	BRD-K29905972	KDR, FLT1, FLT4, CSF1, CYP2C19, CYP3A5, KIT, PDGFRB, PLK4	PDGFR receptor inhibitor, VEGFR inhibitor
lenalidomide	trt_cp	BRD-K05926469	CRBN, TNF, CDH5, PTGS2, TNFSF11	Antineoplastic
tandutinib	trt_cp	BRD-K89162000	FLT3, KIT, PDGFRA, PDGFRB, CSF1R, PDGFD	FLT3 inhibitor, KIT inhibitor, PDGFR receptor inhibitor
balsalazide	trt_cp	BRD-K41410256	PTGS1, PTGS2, ALOX5, PPARG	Cyclooxygenase inhibitor
honokiol	trt_cp	BRD-K98493452	ALOX5, PTGS1, PTGS2	AKT inhibitor
sulfasalazine	trt_cp	BRD-K10670311	PTGS1, PTGS2, ACAT1, ALOX5, CHUK, IKBKB, PLA2G1B, PPARG, SLC46A1, SLC7A11, TBXAS1	Antirheumatic, NFkB pathway inhibitor
A-443644	trt_cp	BRD-K38615104	AKT1, AKT2, AKT3, CDC42BPB, GSK3B, PHKG1, PKIA, PRKACA	AKT inhibitor
WZ-4-145	trt_cp	BRD-U25771771	CSF1R, DDR1, EGFR, PDGFRA, TIE1	EGFR inhibitor
QL-X-138	trt_cp	BRD-U33728988	BTK, JAK3, MKNK2, MTOR, PRKDC	MTOR inhibitor

QL-XII-47	trt_cp	BRD-U86922168	BMX, BTK	BTK inhibitor, Cytoplasmic tyrosine protein kinase BMX inhibitor
GSK-3-inhibitor-IX	trt_cp	BRD-K04923131	ALOX5, GSK3A, GSK3B	Glycogen synthase kinase inhibitor, Lipoxygenase inhibitor
SB-203580	trt_cp	BRD-K99291625	MAPK14, MAPK1, MAPK11, AKT1, ALOX5, CHEK1, CYP2D6, CYP3A4, GAK, GSK3B, LCK, MAPK10, MAPK12, MAPK8, MAPK9, PRKCA, RAF1, RIPK2, ROCK1, RPS6KB1, SGK1, SRC, TNF	p38 MAPK inhibitor
terreic-acid	trt_cp	BRD-A64228451	BTK	BTK inhibitor
hyperforin	trt_cp	BRD-A80775386	NR1I2, ALOX5, PTGS1, TRPC6	Cyclooxygenase inhibitor, Dopamine uptake inhibitor, Interleukin receptor antagonist, Lipoxygenase inhibitor, Serotonin reuptake inhibitor
quizartinib	trt_cp	BRD-K93918653	FLT3, CSF1R, KIT, RET, PDGFRA, PDGFRB	FLT3 inhibitor
linifanib	trt_cp	BRD-K99749624	CSF1R, KDR, PDGFRB, FLT1, FLT3, FLT4, CSF1, KIT, PDGFRA, RET, TEK	PDGFR receptor inhibitor, VEGFR inhibitor
cediranib	trt_cp	BRD-K86930074	KDR, FLT1, FLT4, KIT, PDGFRB, CSF1R, FLT3, PDGFRA	KIT inhibitor, VEGFR inhibitor
dovitinib	trt_cp	BRD-K85402309	EGFR, FGFR3, PDGFRB, CSF1R, FGFR1, FGFR2, FLT1, FLT3, FLT4, INSR, KDR, KIT, PDGFRA	EGFR inhibitor, FLT3 inhibitor, FGFR inhibitor, PDGFR receptor inhibitor, VEGFR inhibitor
pazopanib	trt_cp	BRD-K74514084	KDR, KIT, FLT1, FLT4, PDGFRB, PDGFRA, BRAF, CSF1R, CYP2B6, CYP2C8, CYP2E1, DDR2, FGF1, FGFR1, FGFR3, ITK, SH2B3	KIT inhibitor, PDGFR receptor inhibitor, VEGFR inhibitor

sunitinib	trt_cp	BRD-M64432851	FLT3, KDR, KIT, FLT4, FLT1, PDGFRA, PDGFRB, RET, CSF1R, FGFR1	FLT3 inhibitor, KIT inhibitor, PDGFR receptor inhibitor, RET tyrosine kinase inhibitor, VEGFR inhibitor
sunitinib	trt_cp	BRD-K70511574	FLT3, KDR, KIT, FLT4, FLT1, PDGFRA, PDGFRB, RET, CSF1R, FGFR1	PLK inhibitor
vicriviroc	trt_cp	BRD-M72442222	CCR5	CC chemokine receptor antagonist
rivaroxaban	trt_cp	BRD-K37130656	F10, CYP2J2, CYP3A5	Coagulation inhibitor
montelukast	trt_cp	BRD-A31312900	CYSLTR1, ALOX5, CYP2C8	Leukotriene receptor antagonist
AKT3	trt_oe	ccsbBroad304_07529		
ARRB1	trt_oe	ccsbBroad304_00106		
CD14	trt_oe	ccsbBroad304_00249		
CXCR2	trt_oe	ccsbBroad304_00856		
F10	trt_oe	ccsbBroad304_06188		
F11	trt_oe	ccsbBroad304_00532		
FCGR2A	trt_oe	ccsbBroad304_06198		
GNAI2	trt_oe	ccsbBroad304_06292		
GNGT2	trt_oe	ccsbBroad304_00661		
HLA-DMB	trt_oe	ccsbBroad304_06370		
BTK	trt_oe	ccsbBroad304_00180		
FGR	trt_oe	ccsbBroad304_00564		
CTLA4	trt_oe	ccsbBroad304_00392		
AKT3	trt_sh.cgs	CGS001-10000		
CTLA4	trt_sh.cgs	CGS001-1493		
CFD	trt_sh.cgs	CGS001-1675		
EGR2	trt_sh.cgs	CGS001-1959		
F10	trt_sh.cgs	CGS001-2159		
F11	trt_sh.cgs	CGS001-2160		
FCGR2A	trt_sh.cgs	CGS001-2212		
GNAI2	trt_sh.cgs	CGS001-2771		
GNGT2	trt_sh.cgs	CGS001-2793		
GRK5	trt_sh.cgs	CGS001-2869		
HLA-DMB	trt_sh.cgs	CGS001-3109		
CXCR2	trt_sh.cgs	CGS001-3579		
ARRB1	trt_sh.cgs	CGS001-408		
CD99	trt_sh.cgs	CGS001-4267		
BTK	trt_sh.cgs	CGS001-695		
CD14	trt_sh.cgs	CGS001-929		

ENMD-2076	trt_cp	BRD-K68488863	AURKA, FLT3, KDR, PDGFRA, SRC, CSF1R, EPHA1, FGFR1, FGFR2, FGFR3, FLT4, KIT, PTK2	FLT3 inhibitor, VEGFR inhibitor, Aurora kinase inhibitor
Name	Type	Perturbagen Id	Target	MOA
BW-B70C	trt_cp	BRD-A55946879	ALOX5	Lipoxygenase inhibitor
CGP-57380	trt_cp	BRD-K42500029	AURKB, LCK, MKNK1, MKNK2, SGK1	MAP kinase inhibitor
diclofenac	trt_cp	BRD-K08252256	PTGS1, PTGS2, AKR1C3, ALOX5, ASIC1, ASIC3, CYP2C8, KCNQ2, KCNQ3, PLA2G2A, PPARG, SCN4A	Cyclooxygenase inhibitor
diethylcarbamazine	trt_cp	BRD-K45542189	ALOX5, PTGS1	Lipoxygenase inhibitor
dorsomorphin	trt_cp	BRD-K54233340	ACVR1, BMPR1A, BMPR1B, EPHA2, FKBP1A, FLT1, FLT3, KDR, LCK, MKNK1, PRKAA1, RPS6KA1, SRC	AMPK inhibitor
indirubin	trt_cp	BRD-K53959060	CDK1, CDK5, CCNE1, CDK2, CDK4, CDK5R1, CDK9, GSK3A, GSK3B, LCK, LRRK1, LRRK2	CDK inhibitor, Glycogen synthase kinase inhibitor, Leucine rich repeat kinase inhibitor, SRC inhibitor, Serum/glucocorticoid regulated kinase inhibitor
indirubin	trt_cp	BRD-K19136521	CDK1, CDK5, CCNE1, CDK2, CDK4, CDK5R1, CDK9, GSK3A, GSK3B, LCK, LRRK1, LRRK2	CDK inhibitor
LY-294002	trt_cp	BRD-K27305650	MTOR, PIK3CD, PIK3CG, PIK3CA, PIK3CB, PLK1, PRKDC, AKT1, CHEK1, GSK3B, LCK, MAPK1, MAPK11, MAPK12, MAPK14, MAPK8, PDE2A, PRKCA, ROCK1, RPS6KB1, SGK1	MTOR inhibitor, PI3K inhibitor, DNA dependent protein kinase inhibitor, Phosphodiesterase inhibitor, PLK inhibitor
menadione	trt_cp	BRD-K78126613	AOX1, BGLAP, F10, F2, F7, F9, GGCX, NQO1, NQO2, PKM, PROC, PROS1, PROZ, VKORC1, VKORC1L1	Mitochondrial DNA polymerase inhibitor, Phosphatase inhibitor
rhamnetin	trt_cp	BRD-K37206356	ALOX5, MAPK8	HDAC inhibitor

bisindolylmaleimide-ix	trt_cp	BRD-K06543683	SIRT1, AKT1, GSK3B, LCK, LRRK2, MAPK1, MAPK11, MAPK12, MAPK14, MAPK8, PRKCA, ROCK1, RPS6KB1, SIRT2	CDK inhibitor, PKC inhibitor
simvastatin	trt_cp	BRD-A81772229	HMGCR, CYP2C8, CYP3A4, CYP3A5, ITGB2	HMGCR inhibitor
SRC-kinase-inhibitor-I	trt_cp	BRD-K50495309	CSK, LCK, RIPK2	SRC inhibitor
triciribine	trt_cp	BRD-K80431395	AKT1, AKT2, AKT3	AKT inhibitor
rutin	trt_cp	BRD-K20482099	AKR1C3, AKR1B1, F10	Antioxidant, Capillary stabilizing agent, Nitric oxide scavenger
tyrphostin-AG-82	trt_cp	BRD-K03670461	EGFR, LCK	EGFR inhibitor
aminogenistein	trt_cp	BRD-K04976539	LCK	SRC inhibitor
CDC	trt_cp	BRD-K10870738	ALOX12, ALOX5	Lipoxygenase inhibitor
CP-55940	trt_cp	BRD-A03816571	CNR1, CNR2, CXCR4, GPR55	Cannabinoid receptor agonist
caffeic-acid	trt_cp	BRD-K84709232	ALOX5, MIF, RELA, TNF	Lipoxygenase inhibitor, HIV integrase inhibitor, NFkB pathway inhibitor, Nitric oxide production inhibitor, PPAR receptor modulator, TNF production inhibitor, Tumor necrosis factor production inhibitor
dextromethorphan	trt_cp	BRD-K33211335	SIGMAR1, CHRNA2, CHRNA3, CHRNA4, CHRNA7, CHRNB2, CHRNB4, CYBA, CYBB, CYP3A5, GRIN1, GRIN3A, NCF1, NCF2, NCF4, OPRD1, OPRK1, OPRM1, PGRMC1, RAC1, RAC2, SLC6A2, SLC6A4	Glutamate receptor antagonist, Sigma receptor agonist
GTP-14564	trt_cp	BRD-K16664969	FLT3, CSF1R, KIT, PDGFRB	FLT3 inhibitor, Tyrosine kinase inhibitor
KN-62	trt_cp	BRD-A81177136	AKT1, CAMK2A, CHEK1, LCK, MAPK1, MAPK11, MAPK12, MAPK14, MAPK8, P2RX7, PRKCA, ROCK1, RPS6KB1, SGK1	Calcium-calmodulin dependent protein kinase inhibitor, Purinergic receptor antagonist

kenpaullone	trt_cp	BRD-K37312348	GSK3B, CDK1, CDK5, CCNB1, CDK2, LCK	CDK inhibitor, Glycogen synthase kinase inhibitor
lavendustin-a	trt_cp	BRD-K23583188	EGFR, LCK	EGFR inhibitor
LFM-A13	trt_cp	BRD-A30655177	BTK	BTK inhibitor
lovastatin	trt_cp	BRD-K09416995	HMGCR, CYP3A5, HDAC2, ITGAL, NR1I2	HMGCR inhibitor
meclofenamic-acid	trt_cp	BRD-K50398167	PTGS1, PTGS2, ALOX5, CNR1, KCNQ2, KCNQ3	Cyclooxygenase inhibitor, Prostanoid receptor antagonist
PD-169316	trt_cp	BRD-K77133231	ALOX5	p38 MAPK inhibitor
PD-98059	trt_cp	BRD-K62810658	MAP2K1, AKT1, CHEK1, GSK3B, LCK, MAP2K2, MAPK1, MAPK11, MAPK12, MAPK14, MAPK3, MAPK8, PRKCA, RAF1, ROCK1, RPS6KB1, SGK1	MEK inhibitor, MAP kinase inhibitor
PP-2	trt_cp	BRD-K95785537	SRC, LCK, ABL1, LYN, RIPK2	SRC inhibitor
REV-5901	trt_cp	BRD-A68281735	ALOX5	Leukotriene receptor antagonist, Lipoxygenase inhibitor
SB-202190	trt_cp	BRD-K54330070	MAPK14, AKT1, ALOX5, CHEK1, GSK3B, LCK, MAPK1, MAPK11, MAPK12, MAPK8, PRKCA, ROCK1, RPS6KB1, SGK1	p38 MAPK inhibitor
SKF-86002	trt_cp	BRD-K96809896	ALOX5, MAPK14	p38 MAPK inhibitor
SU-6656	trt_cp	BRD-K97354755	LYN, AURKB, AURKC, BRSK2, CALM1, CAMK2D, LCK, RET, SRC, YES1	SRC inhibitor
4,5,6,7-tetrabromobenzotriazole	trt_cp	BRD-K97118047	CSNK2A1, AKT1, CHEK1, CSNK2A2, CSNK2B, GSK3B, LCK, MAP2K1, MAPK1, MAPK11, MAPK12, MAPK14, MAPK8, PRKCA, ROCK1, RPS6KB1, SGK1	Casein kinase inhibitor
tenidap	trt_cp	BRD-A87479750	ALOX5, KCNJ4, PTGS1	Cyclooxygenase inhibitor
tranilast	trt_cp	BRD-K19533706	HPGDS, HRH1, IDO1, IFNG, IL10, IL2, IL4, SLC22A12, TGFB1, TNF, TRPV2	Angiogenesis inhibitor

U-0126	trt_cp	BRD-K18787491	AKT1, CHEK1, GSK3B, JAK2, LCK, MAP2K1, MAP2K2, MAP2K7, MAPK1, MAPK11, MAPK12, MAPK14, MAPK8, PRKCA, RAF1, ROCK1, RPS6KB1, SGK1	MEK inhibitor
M-3M3FBS	trt_cp	BRD-K09635314	PLCB2, PLCB3, PLCD1, PLCG1, PLCG2	phospholipase activator
zileuton	trt_cp	BRD-A56359832	ALOX5	Leukotriene inhibitor, Lipoxygenase inhibitor
AKT-inhibitor-1-2	trt_cp	BRD-K04887706	AKT1, AKT2, AKT3	AKT inhibitor
BMS-536924	trt_cp	BRD-K34581968	IGF1R, AKT1, CCNE1, CDK2, CYP3A4, ERBB2, INSR, KDR, LCK, MAPK1, MET, PDGFRA, PDGFRB	IGF-1 inhibitor
maraviroc	trt_cp	BRD-A04352665	CCR5, CYP3A5	CC chemokine receptor antagonist
MK-2206	trt_cp	BRD-K68065987	AKT1, AKT2, AKT3	AKT inhibitor
NF-449	trt_cp	BRD-K36324071	P2RX1	Purinergic receptor antagonist
quinine	trt_cp	BRD-U94846492	KCNN4, ABCB1, CYP2D6, GP9, KCNB2, SLC29A4	Hemozoin biocrystallization inhibitor
phorbol-12-myristate-13-acetate	trt_cp	BRD-A15079084	CD4, KCNT2, PRKCA, TRPV4	PKC activator
givinostat	trt_cp	BRD-K13810148	HDAC2, HDAC1, HDAC3, HDAC4, HDAC5, HDAC6, HDAC7, HDAC8, HDAC9, IL1B, IL1R2, IL6R, TNF	HDAC inhibitor
HG-6-64-01	trt_cp	BRD-U37049823	ABL1, BRAF, CSF1R, EGFR, FGFR1, FLT3, KIT, MAPK11, PDGFRB, RET	RAF inhibitor
JW-7-24-1	trt_cp	BRD-U68942961	LCK	LCK Inhibitor
NM-PP1	trt_cp	BRD-K78278890	CAMK2A, LCK, MAPK8, PRKACA, RIPK2, SRC	Mutant kinase inhibitor
mesalazine	trt_cp	BRD-K28849549	PTGS1, PPARG, PTGS2, ALOX5, CHUK, CTNNB1, IKBKB, MPO, NAT1	Cyclooxygenase inhibitor, Lipoxygenase inhibitor, Prostanoid receptor antagonist
sirolimus	trt_cp	BRD-K89626439	MTOR, FKBP1A, CCR5, FGF2	MTOR inhibitor

tozasertib	trt_cp	BRD-K59369769	AURKA, AURKB, ABL1, AURKC, BCR, FLT3, JAK2, DDR2, LCK	Aurora kinase inhibitor, BCR-ABL kinase inhibitor, FLT3 inhibitor, JAK inhibitor
dasatinib	trt_cp	BRD-K49328571	ABL1, FYN, LCK, SRC, KIT, YES1, BCR, EPHA2, LYN, PDGFRB, ABL2, BTK, DDR1, DDR2, PDGFRA, STAT5B	BCR-ABL kinase inhibitor, Ephrin inhibitor, KIT inhibitor, PDGFR receptor inhibitor, SRC inhibitor, Tyrosine kinase inhibitor
imatinib	trt_cp	BRD-K92723993	ABL1, KIT, PDGFRA, BCR, CSF1R, PDGFRB, ABCG2, CYP2C19, CYP2C8, CYP3A5, DDR1, NTRK1, RET	BCR-ABL kinase inhibitor, KIT inhibitor, PDGFR receptor inhibitor
axitinib	trt_cp	BRD-K29905972	KDR, FLT1, FLT4, CSF1, CYP2C19, CYP3A5, KIT, PDGFRB, PLK4	PDGFR receptor inhibitor, VEGFR inhibitor
saracatinib	trt_cp	BRD-K19540840	SRC, ABL1, LCK, YES1	SRC inhibitor
lenalidomide	trt_cp	BRD-K05926469	CRBN, TNF, CDH5, PTGS2, TNFSF11	Antineoplastic
tandutinib	trt_cp	BRD-K89162000	FLT3, KIT, PDGFRA, PDGFRB, CSF1R, PDGFD	FLT3 inhibitor, KIT inhibitor, PDGFR receptor inhibitor
balsalazide	trt_cp	BRD-K41410256	PTGS1, PTGS2, ALOX5, PPARG	Cyclooxygenase inhibitor
honokiol	trt_cp	BRD-K98493452	ALOX5, PTGS1, PTGS2	AKT inhibitor
sulfasalazine	trt_cp	BRD-K10670311	PTGS1, PTGS2, ACAT1, ALOX5, CHUK, IKBKB, PLA2G1B, PPARG, SLC46A1, SLC7A11, TBXAS1	Antirheumatic, NFkB pathway inhibitor
A-443644	trt_cp	BRD-K38615104	AKT1, AKT2, AKT3, CDC42BPB, GSK3B, PHKG1, PKIA, PRKACA	AKT inhibitor
WZ-4-145	trt_cp	BRD-U25771771	CSF1R, DDR1, EGFR, PDGFRA, TIE1	EGFR inhibitor
QL-X-138	trt_cp	BRD-U33728988	BTK, JAK3, MKNK2, MTOR, PRKDC	MTOR inhibitor
WH-4023	trt_cp	BRD-U44618005	ABL1, LCK, SRC	SRC inhibitor
QL-XII-47	trt_cp	BRD-U86922168	BMX, BTK	BTK inhibitor, Cytoplasmic tyrosine protein kinase BMX inhibitor

GSK-3-inhibitor-IX	trt_cp	BRD-K04923131	ALOX5, GSK3A, GSK3B	Glycogen synthase kinase inhibitor, Lipoxygenase inhibitor
SB-203580	trt_cp	BRD-K99291625	MAPK14, MAPK1, MAPK11, AKT1, ALOX5, CHEK1, CYP2D6, CYP3A4, GAK, GSK3B, LCK, MAPK10, MAPK12, MAPK8, MAPK9, PRKCA, RAF1, RIPK2, ROCK1, RPS6KB1, SGK1, SRC, TNF	p38 MAPK inhibitor
terreic-acid	trt_cp	BRD-A64228451	BTK	BTK inhibitor
hyperforin	trt_cp	BRD-A80775386	NR1I2, ALOX5, PTGS1, TRPC6	Cyclooxygenase inhibitor, Dopamine uptake inhibitor, Interleukin receptor antagonist, Lipoxygenase inhibitor, Serotonin reuptake inhibitor
quizartinib	trt_cp	BRD-K93918653	FLT3, CSF1R, KIT, RET, PDGFRA, PDGFRB	FLT3 inhibitor
linifanib	trt_cp	BRD-K99749624	CSF1R, KDR, PDGFRB, FLT1, FLT3, FLT4, CSF1, KIT, PDGFRA, RET, TEK	PDGFR receptor inhibitor, VEGFR inhibitor
cediranib	trt_cp	BRD-K86930074	KDR, FLT1, FLT4, KIT, PDGFRB, CSF1R, FLT3, PDGFRA	KIT inhibitor, VEGFR inhibitor
dovitinib	trt_cp	BRD-K85402309	EGFR, FGFR3, PDGFRB, CSF1R, FGFR1, FGFR2, FLT1, FLT3, FLT4, INSR, KDR, KIT, PDGFRA	EGFR inhibitor, FLT3 inhibitor, FGFR inhibitor, PDGFR receptor inhibitor, VEGFR inhibitor
pazopanib	trt_cp	BRD-K74514084	KDR, KIT, FLT1, FLT4, PDGFRB, PDGFRA, BRAF, CSF1R, CYP2B6, CYP2C8, CYP2E1, DDR2, FGF1, FGFR1, FGFR3, ITK, SH2B3	KIT inhibitor, PDGFR receptor inhibitor, VEGFR inhibitor
sunitinib	trt_cp	BRD-M64432851	FLT3, KDR, KIT, FLT4, FLT1, PDGFRA, PDGFRB, RET, CSF1R, FGFR1	FLT3 inhibitor, KIT inhibitor, PDGFR receptor inhibitor, RET tyrosine kinase inhibitor, VEGFR inhibitor

sunitinib	trt_cp	BRD-K70511574	FLT3, KDR, KIT, FLT4, FLT1, PDGFRA, PDGFRB, RET, CSF1R, FGFR1	PLK inhibitor
vicriviroc	trt_cp	BRD-M72442222	CCR5	CC chemokine receptor antagonist
rivaroxaban	trt_cp	BRD-K37130656	F10, CYP2J2, CYP3A5	Coagulation inhibitor
montelukast	trt_cp	BRD-A31312900	CYSLTR1, ALOX5, CYP2C8	Leukotriene receptor antagonist
staurosporine	trt_cp	BRD-K17953061	CDK2, GSK3B, CAMK2B, CDK1, CDK5, CHEK1, CHRM1, CHRM2, CHRM4, CSK, DAPK1, GPR35, IKBKB, ITK, LCK, LRRK2, MAP2K4, MAP2K6, MAPKAPK2, PAK2, PDPK1, PHKG2, PIK3CG, PIM1, PKN1, PRKACB, PRKCI, PRKCQ, RPS6KA1, STK3, SYK, TNIK, ZAP70	PKC inhibitor
ataluren	trt_cp	BRD-K94830329	CFTR, DMD, F8, F9	CFTR channel agonist, Dystrophin stimulant
AKT3	trt_oe	ccsbBroad304_07529		
ARRB1	trt_oe	ccsbBroad304_00106		
CD14	trt_oe	ccsbBroad304_00249		
CXCR4	trt_oe	ccsbBroad304_01835		
F10	trt_oe	ccsbBroad304_06188		
F11	trt_oe	ccsbBroad304_00532		
FCGR2A	trt_oe	ccsbBroad304_06198		
GNAI2	trt_oe	ccsbBroad304_06292		
GNGT2	trt_oe	ccsbBroad304_00661		
GRK6	trt_oe	ccsbBroad304_00682		
HLA-DMB	trt_oe	ccsbBroad304_06370		
IL7R	trt_oe	ccsbBroad304_00853		
ITGB2	trt_oe	ccsbBroad304_06464		
LCK	trt_oe	ccsbBroad304_13891		
LSP1	trt_oe	ccsbBroad304_06538		
MAP3K8	trt_oe	ccsbBroad304_00348		
NCF2	trt_oe	ccsbBroad304_06619		
PLCG2	trt_oe	ccsbBroad304_06736		
BTK	trt_oe	ccsbBroad304_00180		
FGR	trt_oe	ccsbBroad304_00564		
EGR3	trt_oe	ccsbBroad304_00488		
IL4	trt_oe	ccsbBroad304_00849		

IL6R	trt_oe	ccsbBroad304_00851		
AKT3	trt_sh.cgs	CGS001-10000		
MAP3K8	trt_sh.cgs	CGS001-1326		
CFD	trt_sh.cgs	CGS001-1675		
EGR2	trt_sh.cgs	CGS001-1959		
EGR3	trt_sh.cgs	CGS001-1960		
F10	trt_sh.cgs	CGS001-2159		
F11	trt_sh.cgs	CGS001-2160		
FCGR2A	trt_sh.cgs	CGS001-2212		
GNAI2	trt_sh.cgs	CGS001-2771		
GNGT2	trt_sh.cgs	CGS001-2793		
GRK5	trt_sh.cgs	CGS001-2869		
GRK6	trt_sh.cgs	CGS001-2870		
HLA-DMA	trt_sh.cgs	CGS001-3108		
HLA-DMB	trt_sh.cgs	CGS001-3109		
IL4	trt_sh.cgs	CGS001-3565		
IL6R	trt_sh.cgs	CGS001-3570		
IL7R	trt_sh.cgs	CGS001-3575		
IL11	trt_sh.cgs	CGS001-3589		
ITGB2	trt_sh.cgs	CGS001-3689		
LCK	trt_sh.cgs	CGS001-3932		
LSP1	trt_sh.cgs	CGS001-4046		
ARRB1	trt_sh.cgs	CGS001-408		
CD99	trt_sh.cgs	CGS001-4267		
NCF2	trt_sh.cgs	CGS001-4688		
PLCG2	trt_sh.cgs	CGS001-5336		
NOD2	trt_sh.cgs	CGS001-64127		
BTK	trt_sh.cgs	CGS001-695		
CXCR4	trt_sh.cgs	CGS001-7852		
CD14	trt_sh.cgs	CGS001-929		
ENMD-2076	trt_cp	BRD-K68488863	AURKA, FLT3, KDR, PDGFRA, SRC, CSF1R, EPHA1, FGFR1, FGFR2, FGFR3, FLT4, KIT, PTK2	FLT3 inhibitor, VEGFR inhibitor, Aurora kinase inhibitor

Name	Type	Perturbagen Id	Target	MOA
BMY-45778	trt_cp	BRD-K84895041	PTGIR	IP1 prostacyclin receptor agonist
BW-B70C	trt_cp	BRD-A55946879	ALOX5	Lipoxygenase inhibitor
bisindolylmaleimide	trt_cp	BRD-K49448285	CCND1, CDK4, LRRK2, PDPK1, PIM1, PRKCA, PRKCB, PRKCI, PRKCZ	CDK inhibitor

diclofenac	trt_cp	BRD-K08252256	PTGS1, PTGS2, AKR1C3, ALOX5, ASIC1, ASIC3, CYP2C8, KCNQ2, KCNQ3, PLA2G2A, PPARG, SCN4A	Cyclooxygenase inhibitor
diethylcarbamazine	trt_cp	BRD-K45542189	ALOX5, PTGS1	Lipoxygenase inhibitor
dephostatin	trt_cp	BRD-K60274257	PTPN1, PTPN6	Tyrosine phosphatase inhibitor
dorsomorphin	trt_cp	BRD-K54233340	ACVR1, BMPR1A, BMPR1B, EPHA2, FKBP1A, FLT1, FLT3, KDR, LCK, MKNK1, PRKAA1, RPS6KA1, SRC	AMPK inhibitor
ibuprofen	trt_cp	BRD-A17655518	PTGS2, PTGS1, ASIC1, BCL2, CFTR, CYP2C8, FABP2, PLAT, PPARG, SLC5A8, THBD	Cyclooxygenase inhibitor, NFkB pathway inhibitor
nicotine	trt_cp	BRD-K05395900	CHRNA10, CHRNA9, AOX1, CHAT, CHRNA2, CHRNA3, CHRNA4, CHRNA5, CHRNA6, CHRNA7, CHRNB2, CHRNB3, CHRNB4, CYP19A1, CYP2B6, TBXAS1, TRPA1	Acetylcholine receptor agonist
paclitaxel	trt_cp	BRD-A28746609	TUBB, NR1I2, ABCB1, BCL2, CYP2C8, MAP2, MAP4, MAPT, TLR4, TUBB1	Tubulin inhibitor
rhamnetin	trt_cp	BRD-K37206356	ALOX5, MAPK8	HDAC inhibitor
SKF-96365	trt_cp	BRD-A72703248	CYP3A4, TRPC1, PKD2, TRPC3, TRPC4, TRPC5, TRPV2	Calcium channel blocker
simvastatin	trt_cp	BRD-A81772229	HMGCR, CYP2C8, CYP3A4, CYP3A5, ITGB2	HMGCR inhibitor
TCS-359	trt_cp	BRD-K81376179	FLT3	FLT3 inhibitor
tamoxifen	trt_cp	BRD-K04210847	ESR1, ESR2, CYP3A5, EBP, GPER1, PRKCA, PRKCB, PRKCD, PRKCE, PRKCG, PRKCI, PRKCQ, PRKCZ	Estrogen receptor antagonist, Selective estrogen receptor modulator (SERM)
trequinsin	trt_cp	BRD-K84663978	PDE4A, PDE4B, PDE4C, PDE4D, PDE5A, PTGIR	Phosphodiesterase inhibitor
Y-27632	trt_cp	BRD-K44084986	ROCK1, ROCK2, LRRK2, PKIA, PKN2, PRKACA, PRKCE	Rho associated kinase inhibitor
tyrphostin-AG-1296	trt_cp	BRD-K76064317	FLT3	FLT3 inhibitor

prostaglandin-e1	trt_cp	BRD-K52459643	PTGER1, PTGER2, CATSPER1, CATSPER2, CATSPER3, CATSPER4, PTGDR, PTGER4, PTGIR	Prostanoid receptor agonist
amiloride	trt_cp	BRD-K97181089	SCNN1A, ASIC1, ASIC2, SCNN1B, SCNN1G, AOC1, ASIC3, PKD2, PKD2L1, PLAU, SCNN1D, SLC9A1, TRPC7, TRPV2	Sodium channel blocker
carbacyclin	trt_cp	BRD-K27499107	PPARD, PTGDR, PTGER1, PTGER2, PTGER3, PTGER4, PTGFR, PTGIR, TBXA2R	IP receptor activator, PPAR receptor agonist
CDC	trt_cp	BRD-K10870738	ALOX12, ALOX5	Lipoxygenase inhibitor
CGP-53353	trt_cp	BRD-K32292990	EGFR, PRKCB	EGFR inhibitor, PKC inhibitor
caffeic-acid	trt_cp	BRD-K84709232	ALOX5, MIF, RELA, TNF	Lipoxygenase inhibitor, HIV integrase inhibitor, NFkB pathway inhibitor, Nitric oxide production inhibitor, PPAR receptor modulator, TNF production inhibitor, Tumor necrosis factor production inhibitor
dextromethorphan	trt_cp	BRD-K33211335	SIGMAR1, CHRNA2, CHRNA3, CHRNA4, CHRNA7, CHRNB2, CHRNB4, CYBA, CYBB, CYP3A5, GRIN1, GRIN3A, NCF1, NCF2, NCF4, OPRD1, OPRK1, OPRM1, PGRMC1, RAC1, RAC2, SLC6A2, SLC6A4	Glutamate receptor antagonist, Sigma receptor agonist
gamma-linolenic-acid	trt_cp	BRD-K18059238	TBXAS1	Cyclooxygenase inhibitor, Prostanoid receptor agonist
GTP-14564	trt_cp	BRD-K16664969	FLT3, CSF1R, KIT, PDGFRB	FLT3 inhibitor, Tyrosine kinase inhibitor
hispidin	trt_cp	BRD-K07325606	PREP, PRKCB, PTGS2, XDH	PKC inhibitor
ibudilast	trt_cp	BRD-K16444452	PDE4A, PDE4B, PDE4C, MIF, PDE10A, PDE3A, PDE4D, TLR4, CYSLTR1, IL1B, IL6, PDE11A, PDE5A	Leukotriene receptor antagonist, Phosphodiesterase inhibitor

LFM-A13	trt_cp	BRD-A30655177	BTK	BTK inhibitor
lovastatin	trt_cp	BRD-K09416995	HMGCR, CYP3A5, HDAC2, ITGAL, NR1I2	HMGCR inhibitor
meclofenamic-acid	trt_cp	BRD-K50398167	PTGS1, PTGS2, ALOX5, CNR1, KCNQ2, KCNQ3	Cyclooxygenase inhibitor, Prostanoid receptor antagonist
naloxone	trt_cp	BRD-A41833852	OPRM1, OPRK1, OPRD1, CREB1, ESR1, TLR4	Opioid receptor antagonist
ozagrel	trt_cp	BRD-K19525698	TBXAS1	Thromboxane synthase inhibitor
PD-169316	trt_cp	BRD-K77133231	ALOX5	p38 MAPK inhibitor
D-64406	trt_cp	BRD-K27665173	FLT3, PDGFRA, PDGFRB	PDGFR receptor inhibitor
midostaurin	trt_cp	BRD-K13646352	FLT3, KIT, CCNB1, FLT1, KDR, PDGFRB, PRKCA, PRKCG, VEGFA	FLT3 inhibitor, KIT inhibitor, PKC inhibitor
probenecid	trt_cp	BRD-K95237249	SLC22A6, ABCC1, PANX1, SLC22A11, SLC22A8, SLCO1C1, TRPV2	Uricosuric blocker
picotamide	trt_cp	BRD-K67277431	TBXA2R, TBXAS1	Thromboxane receptor antagonist, Thromboxane synthase inhibitor
REV-5901	trt_cp	BRD-A68281735	ALOX5	Leukotriene receptor antagonist, Lipoxygenase inhibitor
ibuprofen	trt_cp	BRD-K14965640	PTGS2, PTGS1, ASIC1, BCL2, CFTR, CYP2C8, FABP2, PLAT, PPARG, SLC5A8, THBD	Cyclooxygenase inhibitor
SB-202190	trt_cp	BRD-K54330070	MAPK14, AKT1, ALOX5, CHEK1, GSK3B, LCK, MAPK1, MAPK11, MAPK12, MAPK8, PRKCA, ROCK1, RPS6KB1, SGK1	p38 MAPK inhibitor
SKF-86002	trt_cp	BRD-K96809896	ALOX5, MAPK14	p38 MAPK inhibitor
semaxanib	trt_cp	BRD-K63504947	KDR, FLT1, KIT, PDGFRB, FGFR1, FLT3, MET, PDGFRA, RET	VEGFR inhibitor
tenidap	trt_cp	BRD-A87479750	ALOX5, KCNJ4, PTGS1	Cyclooxygenase inhibitor
tranilast	trt_cp	BRD-K19533706	HPGDS, HRH1, IDO1, IFNG, IL10, IL2, IL4, SLC22A12, TGFB1, TNF, TRPV2	Angiogenesis inhibitor

U-46619	trt_cp	BRD-K18757346	PTGDR, PTGDR2, PTGER1, PTGER2, PTGER3, PTGER4, PTGFR, PTGIR, TBXA2R	Thromboxane receptor agonist
M-3M3FBS	trt_cp	BRD-K09635314	PLCB2, PLCB3, PLCD1, PLCG1, PLCG2	phospholipase activator
zileuton	trt_cp	BRD-A56359832	ALOX5	Leukotriene inhibitor, Lipoxygenase inhibitor
imiquimod	trt_cp	BRD-K26657438	TLR7, TLR8	TLR agonist, Interferon inducer
ingenol	trt_cp	BRD-A52650764	PRKCD, PRKCE	PKC activator
PKCbeta-inhibitor	trt_cp	BRD-K89687904	PRKCB	PKC inhibitor
quinine	trt_cp	BRD-U94846492	KCNN4, ABCB1, CYP2D6, GP9, KCNB2, SLC29A4	Hemozoin biocrystallization inhibitor
fostamatinib	trt_cp	BRD-K20285085	SYK, FLT3, RET	SYK inhibitor
TG-101348	trt_cp	BRD-K12502280	JAK2, FLT3, BRD4, JAK1, JAK3, RET, TYK2	FLT3 inhibitor, JAK inhibitor
phorbol-12-myristate-13-acetate	trt_cp	BRD-A15079084	CD4, KCNT2, PRKCA, TRPV4	PKC activator
HG-6-64-01	trt_cp	BRD-U37049823	ABL1, BRAF, CSF1R, EGFR, FGFR1, FLT3, KIT, MAPK11, PDGFRB, RET	RAF inhibitor
treprostinil	trt_cp	BRD-A67438293	PTGIR, CYP2C8, P2RY12, PPARD, PTGDR, PTGER1, PTGER2, PTGER3, PTGER4	Prostacyclin analog
iloprost	trt_cp	BRD-A45664787	PTGIR, PTGER1, PTGER2, PDE4A, PDE4B, PDE4C, PDE4D, PLAT, PTGDR, PTGER3, PTGER4, PTGFR, TBXA2R	Platelet aggregation inhibitor, Prostanoid receptor agonist
mesalazine	trt_cp	BRD-K28849549	PTGS1, PPARG, PTGS2, ALOX5, CHUK, CTNNB1, IKBKB, MPO, NAT1	Cyclooxygenase inhibitor, Lipoxygenase inhibitor, Prostanoid receptor antagonist
tozasertib	trt_cp	BRD-K59369769	AURKA, AURKB, ABL1, AURKC, BCR, FLT3, JAK2, DDR2, LCK	Aurora kinase inhibitor, BCR-ABL kinase inhibitor, FLT3 inhibitor, JAK inhibitor

dasatinib	trt_cp	BRD-K49328571	ABL1, FYN, LCK, SRC, KIT, YES1, BCR, EPHA2, LYN, PDGFRB, ABL2, BTK, DDR1, DDR2, PDGFRA, STAT5B	BCR-ABL kinase inhibitor, Ephrin inhibitor, KIT inhibitor, PDGFR receptor inhibitor, SRC inhibitor, Tyrosine kinase inhibitor
sorafenib	trt_cp	BRD-K49810818	RET, BRAF, FLT3, KDR, RAF1, FLT1, FLT4, KIT, DDR2, FGFR1, PDGFRB, CYP2B6, CYP2C8, CYP3A5, PDGFB, SLCO1B3	FLT3 inhibitor, KIT inhibitor, PDGFR receptor inhibitor, RAF inhibitor, VEGFR inhibitor, RET tyrosine kinase inhibitor
lenalidomide	trt_cp	BRD-K05926469	CRBN, TNF, CDH5, PTGS2, TNFSF11	Antineoplastic
tandutinib	trt_cp	BRD-K89162000	FLT3, KIT, PDGFRA, PDGFRB, CSF1R, PDGFD	FLT3 inhibitor, KIT inhibitor, PDGFR receptor inhibitor
balsalazide	trt_cp	BRD-K41410256	PTGS1, PTGS2, ALOX5, PPARG	Cyclooxygenase inhibitor
honokiol	trt_cp	BRD-K98493452	ALOX5, PTGS1, PTGS2	AKT inhibitor
sulfasalazine	trt_cp	BRD-K10670311	PTGS1, PTGS2, ACAT1, ALOX5, CHUK, IKBKB, PLA2G1B, PPARG, SLC46A1, SLC7A11, TBXAS1	Antirheumatic, NFkB pathway inhibitor
QL-X-138	trt_cp	BRD-U33728988	BTK, JAK3, MKNK2, MTOR, PRKDC	MTOR inhibitor
QL-XII-47	trt_cp	BRD-U86922168	BMX, BTK	BTK inhibitor, Cytoplasmic tyrosine protein kinase BMX inhibitor
GSK-3-inhibitor-IX	trt_cp	BRD-K04923131	ALOX5, GSK3A, GSK3B	Glycogen synthase kinase inhibitor, Lipoxygenase inhibitor
tyrphostin-AG-1295	trt_cp	BRD-K57926513	FLT3, KDR, PDGFRA, PDGFRB	PDGFR receptor inhibitor
SB-203580	trt_cp	BRD-K99291625	MAPK14, MAPK1, MAPK11, AKT1, ALOX5, CHEK1, CYP2D6, CYP3A4, GAK, GSK3B, LCK, MAPK10, MAPK12, MAPK8, MAPK9, PRKCA, RAF1, RIPK2, ROCK1, RPS6KB1, SGK1, SRC, TNF	p38 MAPK inhibitor
terreic-acid	trt_cp	BRD-A64228451	BTK	BTK inhibitor

hyperforin	trt_cp	BRD-A80775386	NR1I2, ALOX5, PTGS1, TRPC6	Cyclooxygenase inhibitor, Dopamine uptake inhibitor, Interleukin receptor antagonist, Lipoxygenase inhibitor, Serotonin reuptake inhibitor
enzastaurin	trt_cp	BRD-K79404599	PRKCB, AKT1, GSK3B, PRKCA, PRKCD, PRKCG	PKC inhibitor
KIN001-127	trt_cp	BRD-A29901043	ITK	ITK inhibitor
quizartinib	trt_cp	BRD-K93918653	FLT3, CSF1R, KIT, RET, PDGFRA, PDGFRB	FLT3 inhibitor
lestaurtinib	trt_cp	BRD-K23192422	FLT3, NTRK1, JAK2, NTRK2, NTRK3	FLT3 inhibitor, Growth factor receptor inhibitor, JAK inhibitor
linifanib	trt_cp	BRD-K99749624	CSF1R, KDR, PDGFRB, FLT1, FLT3, FLT4, CSF1, KIT, PDGFRA, RET, TEK	PDGFR receptor inhibitor, VEGFR inhibitor
cediranib	trt_cp	BRD-K86930074	KDR, FLT1, FLT4, KIT, PDGFRB, CSF1R, FLT3, PDGFRA	KIT inhibitor, VEGFR inhibitor
dovitinib	trt_cp	BRD-K85402309	EGFR, FGFR3, PDGFRB, CSF1R, FGFR1, FGFR2, FLT1, FLT3, FLT4, INSR, KDR, KIT, PDGFRA	EGFR inhibitor, FLT3 inhibitor, FGFR inhibitor, PDGFR receptor inhibitor, VEGFR inhibitor
pazopanib	trt_cp	BRD-K74514084	KDR, KIT, FLT1, FLT4, PDGFRB, PDGFRA, BRAF, CSF1R, CYP2B6, CYP2C8, CYP2E1, DDR2, FGF1, FGFR1, FGFR3, ITK, SH2B3	KIT inhibitor, PDGFR receptor inhibitor, VEGFR inhibitor
sunitinib	trt_cp	BRD-M64432851	FLT3, KDR, KIT, FLT4, FLT1, PDGFRA, PDGFRB, RET, CSF1R, FGFR1	FLT3 inhibitor, KIT inhibitor, PDGFR receptor inhibitor, RET tyrosine kinase inhibitor, VEGFR inhibitor
sunitinib	trt_cp	BRD-K70511574	FLT3, KDR, KIT, FLT4, FLT1, PDGFRA, PDGFRB, RET, CSF1R, FGFR1	PLK inhibitor
montelukast	trt_cp	BRD-A31312900	CYSLTR1, ALOX5, CYP2C8	Leukotriene receptor antagonist

staurosporine	trt_cp	BRD-K17953061	CDK2, GSK3B, CAMK2B, CDK1, CDK5, CHEK1, CHRM1, CHRM2, CHRM4, CSK, DAPK1, GPR35, IKBKB, ITK, LCK, LRRK2, MAP2K4, MAP2K6, MAPKAPK2, PAK2, PDPK1, PHKG2, PIK3CG, PIM1, PKN1, PRKACB, PRKCI, PRKCQ, RPS6KA1, STK3, SYK, TNIK, ZAP70	PKC inhibitor
prostratin	trt_cp	BRD-K91145395	PRKCA, PRKCB, PRKCD, PRKCE, PRKCG, PRKCH, PRKCQ	PKC activator
ataluren	trt_cp	BRD-K94830329	CFTR, DMD, F8, F9	CFTR channel agonist, Dystrophin stimulant
ADCY3	trt_oe	ccsbBroad304_00024		
CD40	trt_oe	ccsbBroad304_00259		
F11	trt_oe	ccsbBroad304_00532		
GNAI2	trt_oe	ccsbBroad304_06292		
GNGT2	trt_oe	ccsbBroad304_00661		
HLA-DMB	trt_oe	ccsbBroad304_06370		
IL7R	trt_oe	ccsbBroad304_00853		
ITGB2	trt_oe	ccsbBroad304_06464		
NCF2	trt_oe	ccsbBroad304_06619		
PLCG2	trt_oe	ccsbBroad304_06736		
PPP3CC	trt_oe	ccsbBroad304_06761		
PRKCB	trt_oe	ccsbBroad304_01282		
PRKCE	trt_oe	ccsbBroad304_01283		
PTPN6	trt_oe	ccsbBroad304_01342		
STAT5A	trt_oe	ccsbBroad304_07008		
TLR8	trt_oe	ccsbBroad304_08275		
TNFSF13	trt_oe	ccsbBroad304_07292		
BTK	trt_oe	ccsbBroad304_00180		
FGR	trt_oe	ccsbBroad304_00564		
EGR3	trt_oe	ccsbBroad304_00488		
ADCY3	trt_sh.cgs	CGS001-109		
CFD	trt_sh.cgs	CGS001-1675		
EGR3	trt_sh.cgs	CGS001-1960		
F11	trt_sh.cgs	CGS001-2160		
FLT3	trt_sh.cgs	CGS001-2322		
GNAI2	trt_sh.cgs	CGS001-2771		
GNGT2	trt_sh.cgs	CGS001-2793		

GRK5	trt_sh.cgs	CGS001-2869		
HLA-DMB	trt_sh.cgs	CGS001-3109		
IL7R	trt_sh.cgs	CGS001-3575		
IL11	trt_sh.cgs	CGS001-3589		
ITGB2	trt_sh.cgs	CGS001-3689		
MYD88	trt_sh.cgs	CGS001-4615		
NCF2	trt_sh.cgs	CGS001-4688		
TLR8	trt_sh.cgs	CGS001-51311		
PLCG2	trt_sh.cgs	CGS001-5336		
PPP3CC	trt_sh.cgs	CGS001-5533		
PRKCB	trt_sh.cgs	CGS001-5579		
PRKCE	trt_sh.cgs	CGS001-5581		
PTPN6	trt_sh.cgs	CGS001-5777		
STAT5A	trt_sh.cgs	CGS001-6776		
BTK	trt_sh.cgs	CGS001-695		
THBD	trt_sh.cgs	CGS001-7056		
TLR4	trt_sh.cgs	CGS001-7099		
TNFSF13	trt_sh.cgs	CGS001-8741		
CD40	trt_sh.cgs	CGS001-958		
AT-9283	trt_cp	BRD-K24576554	AURKA, AURKB, ABL1, BCR, FLT3, JAK2, JAK3, RPS6KA6, STK17A	JAK inhibitor, Aurora kinase inhibitor, ABL inhibitor, BCR-ABL kinase inhibitor, FLT3 inhibitor, Mitotic inhibitor, Protein kinase inhibitor
ENMD-2076	trt_cp	BRD-K68488863	AURKA, FLT3, KDR, PDGFRA, SRC, CSF1R, EPHA1, FGFR1, FGFR2, FGFR3, FLT4, KIT, PTK2	FLT3 inhibitor, VEGFR inhibitor, Aurora kinase inhibitor

Supplementary Table S9. Connectivity map analysis for GSE120622 LUSC.

Name	Type	Perturbagen Id	Target	MOA
BW-B70C	trt_cp	BRD-A55946879	ALOX5	Lipoxygenase inhibitor
colforsin	trt_cp	BRD-A55416093	ADCY2, ADCY5, GNAS	Adenylyl cyclase activator, Adenylate cyclase stimulant
diclofenac	trt_cp	BRD-K08252256	PTGS1, PTGS2, AKR1C3, ALOX5, ASIC1, ASIC3, CYP2C8, KCNQ2, KCNQ3, PLA2G2A, PPARG, SCN4A	Cyclooxygenase inhibitor
diethylcarbamazine	trt_cp	BRD-K45542189	ALOX5, PTGS1	Lipoxygenase inhibitor
ezetimibe	trt_cp	BRD-A41519720	NPC1L1, ANPEP, SOAT1	Niemann-Pick C1-like 1 protein antagonist, Cholesterol inhibitor
flutamide	trt_cp	BRD-K28307902	AR, AHR, CYP2C19	Androgen receptor antagonist
menadione	trt_cp	BRD-K78126613	AOX1, BGLAP, F10, F2, F7, F9, GGCX, NQO1, NQO2, PKM, PROC, PROS1, PROZ, VKORC1, VKORC1L1	Mitochondrial DNA polymerase inhibitor, Phosphatase inhibitor
mexiletine	trt_cp	BRD-A64092382	SCN5A, AHR, CYP2B6, CYP2E1, KCNK2, KCNK3, SCN4A	Sodium channel blocker
nimodipine	trt_cp	BRD-A58048407	CACNA1C, NR3C2, AHR, CACNA1D, CACNA1F, CACNA1S, CACNB1, CACNB2, CACNB3, CACNB4, CFTR	Calcium channel blocker

mepacrine	trt_cp	BRD-A45889380	TP53, AKT1, MTOR, NFKB1, PLA2G1B, PLA2G2A, PLA2G2D, PLA2G4A, PLA2G6, PLCL1	Cytokine production inhibitor, NFkB pathway inhibitor, TP53 activator
rhamnetin	trt_cp	BRD-K37206356	ALOX5, MAPK8	HDAC inhibitor
thalidomide	trt_cp	BRD-A93255169	TNF, CRBN, CYP1A2, CYP2B6, CYP2C19, CYP3A5, FGFR2, NFKB1, ORM1, ORM2, PTGS2	TNF production inhibitor
ZM-39923	trt_cp	BRD-K40624912	JAK1, JAK3	JAK inhibitor
AG-490	trt_cp	BRD-K12357156	JAK2, JAK3, EGFR, STAT3	EGFR inhibitor, ErbB2 inhibitor, JAK inhibitor
arctigenin	trt_cp	BRD-K53523901	ADIPOR1, AHR, CHUK, MAP2K1	MEK inhibitor
ubenimex	trt_cp	BRD-K59574735	LTA4H, ANPEP, RNPEP	Leukotriene inhibitor
CDC	trt_cp	BRD-K10870738	ALOX12, ALOX5	Lipoxygenase inhibitor
caffeic-acid	trt_cp	BRD-K84709232	ALOX5, MIF, RELA, TNF	Lipoxygenase inhibitor, HIV integrase inhibitor, NFkB pathway inhibitor, Nitric oxide production inhibitor, PPAR receptor modulator, TNF production inhibitor, Tumor necrosis factor production inhibitor
Cyclo-[Arg-Gly-Asp-D-Phe-Val]	trt_cp	BRD-K95992530	ITGAV, ITGB3	integrin antagonist
dextromethorphan	trt_cp	BRD-K33211335	SIGMAR1, CHRNA2, CHRNA3, CHRNA4, CHRNA7, CHRNB2, CHRNB4, CYBA, CYBB, CYP3A5, GRIN1, GRIN3A, NCF1, NCF2, NCF4, OPRD1, OPRK1, OPRM1, PGRMC1, RAC1, RAC2, SLC6A2, SLC6A4	Glutamate receptor antagonist, Sigma receptor agonist
GR-144053	trt_cp	BRD-K12120659	ITGB3, ITGA2B	Integrin antagonist
GTP-14564	trt_cp	BRD-K16664969	FLT3, CSF1R, KIT, PDGFRB	FLT3 inhibitor, Tyrosine kinase inhibitor
indole	trt_cp	BRD-K01815685	AHR, IDO1	aryl hydrocarbon receptor agonist
ITE	trt_cp	BRD-K60298136	AHR	Aryl hydrocarbon receptor agonist
JAK3-inhibitor-I	trt_cp	BRD-K72541103	JAK3	JAK inhibitor
JAK3-Inhibitor-II	trt_cp	BRD-K52850071	EGFR, ALK, JAK1, JAK2, JAK3	JAK inhibitor
JAK3-inhibitor-V	trt_cp	BRD-K95676198	JAK3	JAK inhibitor
JAK3-inhibitor-VI	trt_cp	BRD-K04546108	JAK3	JAK inhibitor
LFM-A13	trt_cp	BRD-A30655177	BTK	BTK inhibitor
lovastatin	trt_cp	BRD-K09416995	HMGCR, CYP3A5, HDAC2, ITGAL, NR1I2	HMGCR inhibitor

leflunomide	trt_cp	BRD-K78692225	DHODH, AHR, CYP2C19, JAK3, PTK2B, STAT6	Dihydroorotate dehydrogenase inhibitor, PDGFR receptor inhibitor
meclofenamic-acid	trt_cp	BRD-K50398167	PTGS1, PTGS2, ALOX5, CNR1, KCNQ2, KCNQ3	Cyclooxygenase inhibitor, Prostanoid receptor antagonist
PD-169316	trt_cp	BRD-K77133231	ALOX5	p38 MAPK inhibitor
REV-5901	trt_cp	BRD-A68281735	ALOX5	Leukotriene receptor antagonist, Lipoxygenase inhibitor
SB-202190	trt_cp	BRD-K54330070	MAPK14, AKT1, ALOX5, CHEK1, GSK3B, LCK, MAPK1, MAPK11, MAPK12, MAPK8, PRKCA, ROCK1, RPS6KB1, SGK1	p38 MAPK inhibitor
SKF-86002	trt_cp	BRD-K96809896	ALOX5, MAPK14	p38 MAPK inhibitor
SQ-22536	trt_cp	BRD-A56987319	ADCY1, ADCY5	Adenylyl cyclase inhibitor
tenidap	trt_cp	BRD-A87479750	ALOX5, KCNJ4, PTGS1	Cyclooxygenase inhibitor
zileuton	trt_cp	BRD-A56359832	ALOX5	Leukotriene inhibitor, Lipoxygenase inhibitor
maraviroc	trt_cp	BRD-A04352665	CCR5, CYP3A5	CC chemokine receptor antagonist
TG-101348	trt_cp	BRD-K12502280	JAK2, FLT3, BRD4, JAK1, JAK3, RET, TYK2	FLT3 inhibitor, JAK inhibitor
atorvastatin	trt_cp	BRD-U88459701	HMGCR, DPP4, AHR, CYP3A5, FASLG	HMGCR inhibitor
forskolin	trt_cp	BRD-A70449690	ADCY2, ADCY5, GNAS	Adenylyl cyclase activator
phorbol-12-myristate-13-acetate	trt_cp	BRD-A15079084	CD4, KCNT2, PRKCA, TRPV4	PKC activator
givinostat	trt_cp	BRD-K13810148	HDAC2, HDAC1, HDAC3, HDAC4, HDAC5, HDAC6, HDAC7, HDAC8, HDAC9, IL1B, IL1R2, IL6R, TNF	HDAC inhibitor
HG-6-64-01	trt_cp	BRD-U37049823	ABL1, BRAF, CSF1R, EGFR, FGFR1, FLT3, KIT, MAPK11, PDGFRB, RET	RAF inhibitor
HU-211	trt_cp	BRD-A15010982	CNR1, CNR2, GLRA1, GLRA2, GLRA3, GPR55, GRIN1, NFKB1	Glutamate receptor antagonist
CAY-10470	trt_cp	BRD-K07403598	NFKB1, TNF	NFkB pathway inhibitor
mesalazine	trt_cp	BRD-K28849549	PTGS1, PPARG, PTGS2, ALOX5, CHUK, CTNNB1, IKBKB, MPO, NAT1	Cyclooxygenase inhibitor, Lipoxygenase inhibitor, Prostanoid receptor antagonist
sirolimus	trt_cp	BRD-K89626439	MTOR, FKBP1A, CCR5, FGF2	MTOR inhibitor

dasatinib	trt_cp	BRD-K49328571	ABL1, FYN, LCK, SRC, KIT, YES1, BCR, EPHA2, LYN, PDGFRB, ABL2, BTK, DDR1, DDR2, PDGFRA, STAT5B	BCR-ABL kinase inhibitor, Ephrin inhibitor, KIT inhibitor, PDGFR receptor inhibitor, SRC inhibitor, Tyrosine kinase inhibitor
imatinib	trt_cp	BRD-K92723993	ABL1, KIT, PDGFRA, BCR, CSF1R, PDGFRB, ABCG2, CYP2C19, CYP2C8, CYP3A5, DDR1, NTRK1, RET	BCR-ABL kinase inhibitor, KIT inhibitor, PDGFR receptor inhibitor
lenalidomide	trt_cp	BRD-K05926469	CRBN, TNF, CDH5, PTGS2, TNFSF11	Antineoplastic
tandutinib	trt_cp	BRD-K89162000	FLT3, KIT, PDGFRA, PDGFRB, CSF1R, PDGFD	FLT3 inhibitor, KIT inhibitor, PDGFR receptor inhibitor
sitagliptin	trt_cp	BRD-K19416115	DPP4, CYP2C8, FASLG, HMGCR, SLC22A8	Dipeptidyl peptidase inhibitor
tofacitinib	trt_cp	BRD-K31283835	JAK3, JAK1, JAK2, CYP2C19, TYK2	JAK inhibitor
balsalazide	trt_cp	BRD-K41410256	PTGS1, PTGS2, ALOX5, PPARG	Cyclooxygenase inhibitor
honokiol	trt_cp	BRD-K98493452	ALOX5, PTGS1, PTGS2	AKT inhibitor
sulfasalazine	trt_cp	BRD-K10670311	PTGS1, PTGS2, ACAT1, ALOX5, CHUK, IKBKB, PLA2G1B, PPARG, SLC46A1, SLC7A11, TBXAS1	Antirheumatic, NFkB pathway inhibitor
WZ-4-145	trt_cp	BRD-U25771771	CSF1R, DDR1, EGFR, PDGFRA, TIE1	EGFR inhibitor
QL-X-138	trt_cp	BRD-U33728988	BTK, JAK3, MKNK2, MTOR, PRKDC	MTOR inhibitor
QL-XII-47	trt_cp	BRD-U86922168	BMX, BTK	BTK inhibitor, Cytoplasmic tyrosine protein kinase BMX inhibitor
GSK-3-inhibitor-IX	trt_cp	BRD-K04923131	ALOX5, GSK3A, GSK3B	Glycogen synthase kinase inhibitor, Lipoxygenase inhibitor
ruxolitinib	trt_cp	BRD-K53972329	JAK1, JAK2, TYK2, JAK3	JAK inhibitor
SB-203580	trt_cp	BRD-K99291625	MAPK14, MAPK1, MAPK11, AKT1, ALOX5, CHEK1, CYP2D6, CYP3A4, GAK, GSK3B, LCK, MAPK10, MAPK12, MAPK8, MAPK9, PRKCA, RAF1, RIPK2, ROCK1, RPS6KB1, SGK1, SRC, TNF	p38 MAPK inhibitor
terreic-acid	trt_cp	BRD-A64228451	BTK	BTK inhibitor
3,3'-diindolylmethane	trt_cp	BRD-K37846922	AHR, AR, CYP1A2, GUSB, IDO1	CHK inhibitor, Cytochrome P450 activator, Indoleamine 2,3-dioxygenase inhibitor

hyperforin	trt_cp	BRD-A80775386	NR1I2, ALOX5, PTGS1, TRPC6	Cyclooxygenase inhibitor, Dopamine uptake inhibitor, Interleukin receptor antagonist, Lipoxygenase inhibitor, Serotonin reuptake inhibitor
KIN001-127	trt_cp	BRD-A29901043	ITK	ITK inhibitor
KIN001-055	trt_cp	BRD-K68407802	EGFR, JAK3	EGFR inhibitor, JAK inhibitor, Leukotriene inhibitor, Mediator release inhibitor
quizartinib	trt_cp	BRD-K93918653	FLT3, CSF1R, KIT, RET, PDGFRA, PDGFRB	FLT3 inhibitor
linifanib	trt_cp	BRD-K99749624	CSF1R, KDR, PDGFRB, FLT1, FLT3, FLT4, CSF1, KIT, PDGFRA, RET, TEK	PDGFR receptor inhibitor, VEGFR inhibitor
cediranib	trt_cp	BRD-K86930074	KDR, FLT1, FLT4, KIT, PDGFRB, CSF1R, FLT3, PDGFRA	KIT inhibitor, VEGFR inhibitor
dovitinib	trt_cp	BRD-K85402309	EGFR, FGFR3, PDGFRB, CSF1R, FGFR1, FGFR2, FLT1, FLT3, FLT4, INSR, KDR, KIT, PDGFRA	EGFR inhibitor, FLT3 inhibitor, FGFR inhibitor, PDGFR receptor inhibitor, VEGFR inhibitor
pazopanib	trt_cp	BRD-K74514084	KDR, KIT, FLT1, FLT4, PDGFRB, PDGFRA, BRAF, CSF1R, CYP2B6, CYP2C8, CYP2E1, DDR2, FGF1, FGFR1, FGFR3, ITK, SH2B3	KIT inhibitor, PDGFR receptor inhibitor, VEGFR inhibitor
sunitinib	trt_cp	BRD-M64432851	FLT3, KDR, KIT, FLT4, FLT1, PDGFRA, PDGFRB, RET, CSF1R, FGFR1	FLT3 inhibitor, KIT inhibitor, PDGFR receptor inhibitor, RET tyrosine kinase inhibitor, VEGFR inhibitor
cinacalcet	trt_cp	BRD-K73838513	CASR	Calcium channel activator
sunitinib	trt_cp	BRD-K70511574	FLT3, KDR, KIT, FLT4, FLT1, PDGFRA, PDGFRB, RET, CSF1R, FGFR1	PLK inhibitor
vicriviroc	trt_cp	BRD-M72442222	CCR5	CC chemokine receptor antagonist
montelukast	trt_cp	BRD-A31312900	CYSLTR1, ALOX5, CYP2C8	Leukotriene receptor antagonist
aspirin	trt_cp	BRD-K11433652	PTGS1, PTGS2, TP53, AKR1C1, ASIC3, EDNRA, HSPA5, IKBKB, NFKB1, NFKB2, NFKBIA, PRKAA1, PRKAA2, PRKAB1, PRKAB2, PRKAG1, PRKAG2, PRKAG3, RPS6KA3	Cyclooxygenase inhibitor

staurosporine	trt_cp	BRD-K17953061	CDK2, GSK3B, CAMK2B, CDK1, CDK5, CHEK1, CHRM1, CHRM2, CHRM4, CSK, DAPK1, GPR35, IKBKB, ITK, LCK, LRRK2, MAP2K4, MAP2K6, MAPKAPK2, PAK2, PDPK1, PHKG2, PIK3CG, PIM1, PKN1, PRKACB, PRKCI, PRKCQ, RPS6KA1, STK3, SYK, TNIK, ZAP70	PKC inhibitor
vidarabine	trt_cp	BRD-K72093121	ADCY5	Antiviral
CD19	trt_oe	ccsbBroad304_00250		
CD40	trt_oe	ccsbBroad304_00259		
CXCL1	trt_oe	ccsbBroad304_00695		
F11	trt_oe	ccsbBroad304_00532		
FAS	trt_oe	ccsbBroad304_00087		
GNGT2	trt_oe	ccsbBroad304_00661		
IFIH1	trt_oe	ccsbBroad304_08839		
IL15	trt_oe	ccsbBroad304_00863		
IL1R2	trt_oe	ccsbBroad304_01833		
IL21R	trt_oe	ccsbBroad304_03148		
IL2RB	trt_oe	ccsbBroad304_06438		
PSME1	trt_oe	ccsbBroad304_01326		
STAT5A	trt_oe	ccsbBroad304_07008		
BTK	trt_oe	ccsbBroad304_00180		
ANPEP	trt_oe	ccsbBroad304_05817		
BCL10	trt_oe	ccsbBroad304_02048		
CTLA4	trt_oe	ccsbBroad304_00392		
FASLG	trt_oe	ccsbBroad304_00088		
GNG8	trt_oe	ccsbBroad304_04619		
MAPKAPK2	trt_oe	ccsbBroad304_07381		
NFKB1	trt_oe	ccsbBroad304_06637		
RELB	trt_oe	ccsbBroad304_01388		
ADCY5	trt_sh.cgs	CGS001-111		
CTLA4	trt_sh.cgs	CGS001-1493		
AHR	trt_sh.cgs	CGS001-196		
F5	trt_sh.cgs	CGS001-2153		
F7	trt_sh.cgs	CGS001-2155		
F11	trt_sh.cgs	CGS001-2160		
GPRC6A	trt_sh.cgs	CGS001-222545		
GNGT2	trt_sh.cgs	CGS001-2793		
ANPEP	trt_sh.cgs	CGS001-290		
CXCL1	trt_sh.cgs	CGS001-2919		
HLA-DMA	trt_sh.cgs	CGS001-3108		
FAS	trt_sh.cgs	CGS001-355		
FASLG	trt_sh.cgs	CGS001-356		

IL2RB	trt_sh.cgs	CGS001-3560		
IL5	trt_sh.cgs	CGS001-3567		
IL15	trt_sh.cgs	CGS001-3600		
IRF1	trt_sh.cgs	CGS001-3659		
ITGB3	trt_sh.cgs	CGS001-3690		
JAK3	trt_sh.cgs	CGS001-3718		
NFATC2	trt_sh.cgs	CGS001-4773		
NFKB1	trt_sh.cgs	CGS001-4790		
NFKB2	trt_sh.cgs	CGS001-4791		
PSME1	trt_sh.cgs	CGS001-5720		
RELB	trt_sh.cgs	CGS001-5971		
IFIH1	trt_sh.cgs	CGS001-64135		
SOS1	trt_sh.cgs	CGS001-6654		
STAT5A	trt_sh.cgs	CGS001-6776		
BTK	trt_sh.cgs	CGS001-695		
IL1R2	trt_sh.cgs	CGS001-7850		
BCL10	trt_sh.cgs	CGS001-8915		
MAPKAPK2	trt_sh.cgs	CGS001-9261		
CD19	trt_sh.cgs	CGS001-930		
GNG8	trt_sh.cgs	CGS001-94235		
CD40	trt_sh.cgs	CGS001-958		
AT-9283	trt_cp	BRD-K24576554	AURKA, AURKB, ABL1, BCR, FLT3, JAK2, JAK3, RPS6KA6, STK17A	JAK inhibitor, Aurora kinase inhibitor, ABL inhibitor, BCR-ABL kinase inhibitor, FLT3 inhibitor, Mitotic inhibitor, Protein kinase inhibitor
ENMD-2076	trt_cp	BRD-K68488863	AURKA, FLT3, KDR, PDGFRA, SRC, CSF1R, EPHA1, FGFR1, FGFR2, FGFR3, FLT4, KIT, PTK2	FLT3 inhibitor, VEGFR inhibitor, Aurora kinase inhibitor

Supplementary Table S10. Connectivity map analysis of GSE111907 LUAD.

Name	Type	Perturbagen Id	Target	MOA
colforsin	trt_cp	BRD-A55416093	ADCY2, ADCY5, GNAS	Adenylyl cyclase activator, Adenylate cyclase stimulant
dorsomorphin	trt_cp	BRD-K54233340	ACVR1, BMPR1A, BMPR1B, EPHA2, FKBP1A, FLT1, FLT3, KDR, LCK, MKNK1, PRKAA1, RPS6KA1, SRC	AMPK inhibitor
ibuprofen	trt_cp	BRD-A17655518	PTGS2, PTGS1, ASIC1, BCL2, CFTR, CYP2C8, FABP2, PLAT, PPARG, SLC5A8, THBD	Cyclooxygenase inhibitor, NFkB pathway inhibitor
indoprofen	trt_cp	BRD-A44090213	PTGS1, PTGS2, CXCR1, CXCR2	Cyclooxygenase inhibitor, Prostanoid receptor antagonist
LY-294002	trt_cp	BRD-K27305650	MTOR, PIK3CD, PIK3CG, PIK3CA, PIK3CB, PLK1, PRKDC, AKT1, CHEK1, GSK3B, LCK, MAPK1, MAPK11, MAPK12, MAPK14, MAPK8, PDE2A, PRKCA, ROCK1, RPS6KB1, SGK1	MTOR inhibitor, PI3K inhibitor, DNA dependent protein kinase inhibitor, Phosphodiesterase inhibitor, PLK inhibitor
menadione	trt_cp	BRD-K78126613	AOX1, BGLAP, F10, F2, F7, F9, GGCX, NQO1, NQO2, PKM, PROC, PROS1, PROZ, VKORC1, VKORC1L1	Mitochondrial DNA polymerase inhibitor, Phosphatase inhibitor
paclitaxel	trt_cp	BRD-A28746609	TUBB, NR1I2, ABCB1, BCL2, CYP2C8, MAP2, MAP4, MAPT, TLR4, TUBB1	Tubulin inhibitor
mepacrine	trt_cp	BRD-A45889380	TP53, AKT1, MTOR, NFKB1, PLA2G1B, PLA2G2A,	Cytokine production inhibitor, NFkB pathway inhibitor, TP53 activator

			PLA2G2D, PLA2G4A, PLA2G6, PLCL1	
bisindolylmaleimide-ix	trt_cp	BRD-K06543683	SIRT1, AKT1, GSK3B, LCK, LRRK2, MAPK1, MAPK11, MAPK12, MAPK14, MAPK8, PRKCA, ROCK1, RPS6KB1, SIRT2	CDK inhibitor, PKC inhibitor
TCS-359	trt_cp	BRD-K81376179	FLT3	FLT3 inhibitor
triciribine	trt_cp	BRD-K80431395	AKT1, AKT2, AKT3	AKT inhibitor
manumycin-a	trt_cp	BRD-K78599730	FNTA, IKBKB	Farnesyltransferase inhibitor, NFkB pathway inhibitor
rutin	trt_cp	BRD-K20482099	AKR1C3, AKR1B1, F10	Antioxidant, Capillary stabilizing agent, Nitric oxide scavenger
tyrphostin-AG-1296	trt_cp	BRD-K76064317	FLT3	FLT3 inhibitor
BH3I-1	trt_cp	BRD-A38913120	BCL2	BCL inhibitor
BML-257	trt_cp	BRD-K32584078	AKT1	AKT inhibitor, HCV inhibitor
BCL2-inhibitor	trt_cp	BRD-K39111395	BCL2	BCL inhibitor
deguelin	trt_cp	BRD-K61401890	AKT1, PTGS2	NADH-ubiquinone oxidoreductase (Complex I) inhibitor
GTP-14564	trt_cp	BRD-K16664969	FLT3, CSF1R, KIT, PDGFRB	FLT3 inhibitor, Tyrosine kinase inhibitor
IKK-16	trt_cp	BRD-K14618467	IKBKB	IKK inhibitor
IKK-2-inhibitor-V	trt_cp	BRD-K74305673	IKBKB	IKK inhibitor, NFkB pathway inhibitor
KN-62	trt_cp	BRD-A81177136	AKT1, CAMK2A, CHEK1, LCK, MAPK1, MAPK11, MAPK12, MAPK14, MAPK8, P2RX7, PRKCA, ROCK1, RPS6KB1, SGK1	Calcium-calmodulin dependent protein kinase inhibitor, Purinergic receptor antagonist
oxindole-I	trt_cp	BRD-K51816706	AKT1, KDR, PDPK1, RET	VEGFR inhibitor
PD-98059	trt_cp	BRD-K62810658	MAP2K1, AKT1, CHEK1, GSK3B, LCK, MAP2K2, MAPK1, MAPK11, MAPK12, MAPK14, MAPK3, MAPK8, PRKCA, RAF1, ROCK1, RPS6KB1, SGK1	MEK inhibitor, MAP kinase inhibitor

D-64406	trt_cp	BRD-K27665173	FLT3, PDGFRA, PDGFRB	PDGFR receptor inhibitor
midostaurin	trt_cp	BRD-K13646352	FLT3, KIT, CCNB1, FLT1, KDR, PDGFRB, PRKCA, PRKCG, VEGFA	FLT3 inhibitor, KIT inhibitor, PKC inhibitor
pseudoephedrine	trt_cp	BRD-K84175871	ADRA1A, ADRA2A, ADRB1, ADRB2, ATF1, ATF2, ATF3, ATF4, ATF5, ATF6, ATF7, CXCL8, FOS, HRH1, IL2, JDP2, JUN, NFATC1, SLC6A2, SLC6A3, SLC6A4, TNF	Adrenergic receptor agonist
parthenolide	trt_cp	BRD-K98548675	ADIPOR2, IKBKB, RELA	NFkB pathway inhibitor, Adiponectin receptor agonist
ibuprofen	trt_cp	BRD-K14965640	PTGS2, PTGS1, ASIC1, BCL2, CFTR, CYP2C8, FABP2, PLAT, PPARG, SLC5A8, THBD	Cyclooxygenase inhibitor
SB-202190	trt_cp	BRD-K54330070	MAPK14, AKT1, ALOX5, CHEK1, GSK3B, LCK, MAPK1, MAPK11, MAPK12, MAPK8, PRKCA, ROCK1, RPS6KB1, SGK1	p38 MAPK inhibitor
SB-225002	trt_cp	BRD-K61323504	CXCR2	CC chemokine receptor antagonist
IKK-2-inhibitor	trt_cp	BRD-K26373640	IKBKB	IKK inhibitor, SYK inhibitor
SQ-22536	trt_cp	BRD-A56987319	ADCY1, ADCY5	Adenylyl cyclase inhibitor
semaxanib	trt_cp	BRD-K63504947	KDR, FLT1, KIT, PDGFRB, FGFR1, FLT3, MET, PDGFRA, RET	VEGFR inhibitor
4,5,6,7-tetrabromobenzotriazole	trt_cp	BRD-K97118047	CSNK2A1, AKT1, CHEK1, CSNK2A2, CSNK2B, GSK3B, LCK, MAP2K1, MAPK1, MAPK11, MAPK12, MAPK14, MAPK8, PRKCA, ROCK1, RPS6KB1, SGK1	Casein kinase inhibitor
tranilast	trt_cp	BRD-K19533706	HPGDS, HRH1, IDO1, IFNG, IL10, IL2, IL4, SLC22A12, TGFB1, TNF, TRPV2	Angiogenesis inhibitor

U-0126	trt_cp	BRD-K18787491	AKT1, CHEK1, GSK3B, JAK2, LCK, MAP2K1, MAP2K2, MAP2K7, MAPK1, MAPK11, MAPK12, MAPK14, MAPK8, PRKCA, RAF1, ROCK1, RPS6KB1, SGK1	MEK inhibitor
gossypol	trt_cp	BRD-K19295594	BCL2, BCL2L1, MCL1, BCL2L2, CTGF, EGF	BCL inhibitor, MCL1 inhibitor
ABT-737	trt_cp	BRD-K56301217	BCL2, BCL2L1, BCL2L2	BCL inhibitor
navitoclax	trt_cp	BRD-K82746043	BCL2, BCL2L1, BCL2L2	BCL inhibitor
AKT-inhibitor-IV	trt_cp	BRD-K28296557	AKT1	AKT inhibitor
AKT-inhibitor-1-2	trt_cp	BRD-K04887706	AKT1, AKT2, AKT3	AKT inhibitor
BMS-536924	trt_cp	BRD-K34581968	IGF1R, AKT1, CCNE1, CDK2, CYP3A4, ERBB2, INSR, KDR, LCK, MAPK1, MET, PDGFRA, PDGFRB	IGF-1 inhibitor
BMS-754807	trt_cp	BRD-K13049116	IGF1R, AKT1	IGF-1 inhibitor
MK-2206	trt_cp	BRD-K68065987	AKT1, AKT2, AKT3	AKT inhibitor
fostamatinib	trt_cp	BRD-K20285085	SYK, FLT3, RET	SYK inhibitor
TG-101348	trt_cp	BRD-K12502280	JAK2, FLT3, BRD4, JAK1, JAK3, RET, TYK2	FLT3 inhibitor, JAK inhibitor
TPCA-1	trt_cp	BRD-K51575138	IKBKB	IKK inhibitor
TW-37	trt_cp	BRD-K28360340	BCL2, BCL2L1, MCL1	BCL inhibitor
auranofin	trt_cp	BRD-A79465854	IKBKB, PRDX5, TRPA1, TXNRD1, TXNRD2	NFkB pathway inhibitor
forskolin	trt_cp	BRD-A70449690	ADCY2, ADCY5, GNAS	Adenylyl cyclase activator
myriocin	trt_cp	BRD-A76279427	AKT1, SPTLC1, SPTLC2, SPTLC3	Serine palmitoyltransferase inhibitor
HG-6-64-01	trt_cp	BRD-U37049823	ABL1, BRAF, CSF1R, EGFR, FGFR1, FLT3, KIT, MAPK11, PDGFRB, RET	RAF inhibitor
mesalazine	trt_cp	BRD-K28849549	PTGS1, PPARG, PTGS2, ALOX5, CHUK, CTNNB1, IKBKB, MPO, NAT1	Cyclooxygenase inhibitor, Lipoxygenase inhibitor, Prostanoid receptor antagonist

alvocidib	trt_cp	BRD-K87909389	CDK2, CDK4, CDK1, CDK6, CDK7, CDK9, CDK5, CDK8, EGFR, PYGM, BCL2, BIRC5, CCNT1, MCL1, XIAP	CDK inhibitor
tozasertib	trt_cp	BRD-K59369769	AURKA, AURKB, ABL1, AURKC, BCR, FLT3, JAK2, DDR2, LCK	Aurora kinase inhibitor, BCR-ABL kinase inhibitor, FLT3 inhibitor, JAK inhibitor
parthenolide	trt_cp	BRD-K28120222	ADIPOR2, IKBKB, RELA	NFkB pathway inhibitor
sorafenib	trt_cp	BRD-K49810818	RET, BRAF, FLT3, KDR, RAF1, FLT1, FLT4, KIT, DDR2, FGFR1, PDGFRB, CYP2B6, CYP2C8, CYP3A5, PDGFB, SLCO1B3	FLT3 inhibitor, KIT inhibitor, PDGFR receptor inhibitor, RAF inhibitor, VEGFR inhibitor, RET tyrosine kinase inhibitor
tandutinib	trt_cp	BRD-K89162000	FLT3, KIT, PDGFRA, PDGFRB, CSF1R, PDGFD	FLT3 inhibitor, KIT inhibitor, PDGFR receptor inhibitor
obatoclax	trt_cp	BRD-K15600710	BCL2, BCL2L1, MCL1	BCL inhibitor
HA-14-1	trt_cp	BRD-A13807286	BCL2	BCL inhibitor
docetaxel	trt_cp	BRD-K63265447	TUBB, BCL2, MAP2, MAP4, MAPT, NR1I2, TUBB1	Tubulin inhibitor
sulfasalazine	trt_cp	BRD-K10670311	PTGS1, PTGS2, ACAT1, ALOX5, CHUK, IKBKB, PLA2G1B, PPARG, SLC46A1, SLC7A11, TBXAS1	Antirheumatic, NFkB pathway inhibitor
A-443644	trt_cp	BRD-K38615104	AKT1, AKT2, AKT3, CDC42BPB, GSK3B, PHKG1, PKIA, PRKACA	AKT inhibitor
withaferin-a	trt_cp	BRD-A52193669	ACHE, BCHE, IKBKB	IKK inhibitor
guggulsterone	trt_cp	BRD-A59808129	NR1H4, PGR, AR, ESR1, IKBKB, NR1I2, NR3C1, NR3C2	Cholesterol inhibitor, Estrogen receptor agonist, FXR antagonist, IKK inhibitor, Pregnane X receptor agonist, Progesterone receptor agonist
tyrphostin-AG-1295	trt_cp	BRD-K57926513	FLT3, KDR, PDGFRA, PDGFRB	PDGFR receptor inhibitor

SB-203580	trt_cp	BRD-K99291625	MAPK14, MAPK1, MAPK11, AKT1, ALOX5, CHEK1, CYP2D6, CYP3A4, GAK, GSK3B, LCK, MAPK10, MAPK12, MAPK8, MAPK9, PRKCA, RAF1, RIPK2, ROCK1, RPS6KB1, SGK1, SRC, TNF	p38 MAPK inhibitor
GR-235	trt_cp	BRD-K26674531	ESR1, IKBKB, NR1H4, NR1I2, PGR	Estrogen receptor agonist, FXR antagonist, Progesterone receptor agonist
enzastaurin	trt_cp	BRD-K79404599	PRKCB, AKT1, GSK3B, PRKCA, PRKCD, PRKCG	PKC inhibitor
BMS-345541	trt_cp	BRD-K13566078	IKBKB, CHUK	IKK inhibitor
BX-912	trt_cp	BRD-K49669041	PDPK1, AKT2, CDK2, CHEK1, GSK3B, KDR, PDK1	Pyruvate dehydrogenase kinase inhibitor
quizartinib	trt_cp	BRD-K93918653	FLT3, CSF1R, KIT, RET, PDGFRA, PDGFRB	FLT3 inhibitor
lestaurtinib	trt_cp	BRD-K23192422	FLT3, NTRK1, JAK2, NTRK2, NTRK3	FLT3 inhibitor, Growth factor receptor inhibitor, JAK inhibitor
linifanib	trt_cp	BRD-K99749624	CSF1R, KDR, PDGFRB, FLT1, FLT3, FLT4, CSF1, KIT, PDGFRA, RET, TEK	PDGFR receptor inhibitor, VEGFR inhibitor
cediranib	trt_cp	BRD-K86930074	KDR, FLT1, FLT4, KIT, PDGFRB, CSF1R, FLT3, PDGFRA	KIT inhibitor, VEGFR inhibitor
dovitinib	trt_cp	BRD-K85402309	EGFR, FGFR3, PDGFRB, CSF1R, FGFR1, FGFR2, FLT1, FLT3, FLT4, INSR, KDR, KIT, PDGFRA	EGFR inhibitor, FLT3 inhibitor, FGFR inhibitor, PDGFR receptor inhibitor, VEGFR inhibitor
canertinib	trt_cp	BRD-K50168500	EGFR, ERBB2, ERBB4, AKT1	EGFR inhibitor
sunitinib	trt_cp	BRD-M64432851	FLT3, KDR, KIT, FLT4, FLT1, PDGFRA, PDGFRB, RET, CSF1R, FGFR1	FLT3 inhibitor, KIT inhibitor, PDGFR receptor inhibitor, RET tyrosine kinase inhibitor, VEGFR inhibitor
cinacalcet	trt_cp	BRD-K73838513	CASR	Calcium channel activator
sunitinib	trt_cp	BRD-K70511574	FLT3, KDR, KIT, FLT4, FLT1, PDGFRA, PDGFRB, RET, CSF1R, FGFR1	PLK inhibitor
rivaroxaban	trt_cp	BRD-K37130656	F10, CYP2J2, CYP3A5	Coagulation inhibitor

cyclophosphamide	trt_cp	BRD-A09722536	BCL2, CYP2B6, CYP2C19, CYP3A5, LGALS1	DNA alkylating agent
4-hydroxy-2-nonenal	trt_cp	BRD-A15914070	IKBKB	Cytotoxic lipid peroxidation product
aspirin	trt_cp	BRD-K11433652	PTGS1, PTGS2, TP53, AKR1C1, ASIC3, EDNRA, HSPA5, IKBKB, NFKB1, NFKB2, NFKBIA, PRKAA1, PRKAA2, PRKAB1, PRKAB2, PRKAG1, PRKAG2, PRKAG3, RPS6KA3	Cyclooxygenase inhibitor
staurosporine	trt_cp	BRD-K17953061	CDK2, GSK3B, CAMK2B, CDK1, CDK5, CHEK1, CHRM1, CHRM2, CHRM4, CSK, DAPK1, GPR35, IKBKB, ITK, LCK, LRRK2, MAP2K4, MAP2K6, MAPKAPK2, PAK2, PDPK1, PHKG2, PIK3CG, PIM1, PKN1, PRKACB, PRKCI, PRKCQ, RPS6KA1, STK3, SYK, TNIK, ZAP70	PKC inhibitor
edaravone	trt_cp	BRD-K35458079	BCL2	Nootropic agent
vidarabine	trt_cp	BRD-K72093121	ADCY5	Antiviral
AKT1	trt_oe	ccsbBroad304_00046		
AKT2	trt_oe	ccsbBroad304_00047		
CD19	trt_oe	ccsbBroad304_00250		
CXCR2	trt_oe	ccsbBroad304_00856		
F10	trt_oe	ccsbBroad304_06188		
GNAI1	trt_oe	ccsbBroad304_06291		
IFNGR1	trt_oe	ccsbBroad304_00834		
IKBKB	trt_oe	ccsbBroad304_00841		
IL2	trt_oe	ccsbBroad304_00846		
ADCY5	trt_sh.cgs	CGS001-111		
CALML6	trt_sh.cgs	CGS001-163688		
AKT1	trt_sh.cgs	CGS001-207		
AKT2	trt_sh.cgs	CGS001-208		
F5	trt_sh.cgs	CGS001-2153		
F10	trt_sh.cgs	CGS001-2159		
GPRC6A	trt_sh.cgs	CGS001-222545		
FLT3	trt_sh.cgs	CGS001-2322		
GNAI1	trt_sh.cgs	CGS001-2770		
GRK5	trt_sh.cgs	CGS001-2869		
IFNGR1	trt_sh.cgs	CGS001-3459		
IKBKB	trt_sh.cgs	CGS001-3551		

IL2	trt_sh.cgs	CGS001-3558		
CXCR2	trt_sh.cgs	CGS001-3579		
BCL2	trt_sh.cgs	CGS001-596		
CD19	trt_sh.cgs	CGS001-930		
AT-9283	trt_cp	BRD-K24576554	AURKA, AURKB, ABL1, BCR, FLT3, JAK2, JAK3, RPS6KA6, STK17A	JAK inhibitor, Aurora kinase inhibitor, ABL inhibitor, BCR-ABL kinase inhibitor, FLT3 inhibitor, Mitotic inhibitor, Protein kinase inhibitor
ENMD-2076	trt_cp	BRD-K68488863	AURKA, FLT3, KDR, PDGFRA, SRC, CSF1R, EPHA1, FGFR1, FGFR2, FGFR3, FLT4, KIT, PTK2	FLT3 inhibitor, VEGFR inhibitor, Aurora kinase inhibitor
Name	Type	Perturbagen Id	Target	MOA
BMY-45778	trt_cp	BRD-K84895041	PTGIR	IP1 prostacyclin receptor agonist
bisindolylmaleimide	trt_cp	BRD-K49448285	CCND1, CDK4, LRRK2, PDPK1, PIM1, PRKCA, PRKCB, PRKCI, PRKCZ	CDK inhibitor
caffeine	trt_cp	BRD-K02404261	ADORA1, ADORA2A, ADORA2B, ATM, ITPR1, RYR1, ADORA3, ATR, ITPR2, ITPR3, PDE10A, PDE11A, PDE1A, PDE1B, PDE1C, PDE2A, PDE3A, PDE3B, PDE4A, PDE4B, PDE4C, PDE4D, PDE5A, PDE6A, PDE6B, PDE6C, PDE7A, PDE7B, PDE8A, PDE8B, PDE9A, PIK3CA, PIK3CB, PIK3CD, PRKDC, RYR2, RYR3	Adenosine receptor antagonist, Diuretic, Phosphodiesterase inhibitor
celecoxib	trt_cp	BRD-K02637541	PTGS2, CA12, CASP3, CYP2C19, PDPK1	Cyclooxygenase inhibitor
colforsin	trt_cp	BRD-A55416093	ADCY2, ADCY5, GNAS	Adenylyl cyclase activator, Adenylate cyclase stimulant
dorsomorphin	trt_cp	BRD-K54233340	ACVR1, BMPR1A, BMPR1B, EPHA2, FKBP1A, FLT1, FLT3, KDR, LCK, MKNK1, PRKAA1, RPS6KA1, SRC	AMPK inhibitor
indoprofen	trt_cp	BRD-A44090213	PTGS1, PTGS2, CXCR1, CXCR2	Cyclooxygenase inhibitor, Prostanoid receptor antagonist
kawain	trt_cp	BRD-K09497549	MTOR	Calcium channel modulator, MTOR

				inhibitor, Sodium channel blocker
LY-294002	trt_cp	BRD-K27305650	MTOR, PIK3CD, PIK3CG, PIK3CA, PIK3CB, PLK1, PRKDC, AKT1, CHEK1, GSK3B, LCK, MAPK1, MAPK11, MAPK12, MAPK14, MAPK8, PDE2A, PRKCA, ROCK1, RPS6KB1, SGK1	MTOR inhibitor, PI3K inhibitor, DNA dependent protein kinase inhibitor, Phosphodiesterase inhibitor, PLK inhibitor
LY-83583	trt_cp	BRD-K62792802	GUCY1A2	Guanylyl cyclase inhibitor
menadione	trt_cp	BRD-K78126613	AOX1, BGLAP, F10, F2, F7, F9, GGCX, NQO1, NQO2, PKM, PROC, PROS1, PROZ, VKORC1, VKORC1L1	Mitochondrial DNA polymerase inhibitor, Phosphatase inhibitor
mepacrine	trt_cp	BRD-A45889380	TP53, AKT1, MTOR, NFKB1, PLA2G1B, PLA2G2A, PLA2G2D, PLA2G4A, PLA2G6, PLCL1	Cytokine production inhibitor, NFkB pathway inhibitor, TP53 activator
TCS-359	trt_cp	BRD-K81376179	FLT3	FLT3 inhibitor
triciribine	trt_cp	BRD-K80431395	AKT1, AKT2, AKT3	AKT inhibitor
tamoxifen	trt_cp	BRD-K04210847	ESR1, ESR2, CYP3A5, EBP, GPER1, PRKCA, PRKCB, PRKCD, PRKCE, PRKCG, PRKCI, PRKCQ, PRKCZ	Estrogen receptor antagonist, Selective estrogen receptor modulator (SERM)
trequinsin	trt_cp	BRD-K84663978	PDE4A, PDE4B, PDE4C, PDE4D, PDE5A, PTGIR	Phosphodiesterase inhibitor
wortmannin	trt_cp	BRD-A11678676	PIK3CA, PIK3CG, PLK1, ATM, ATR, MTOR, PI4KA, PI4KB, PIK3CD, PIK3R1, PLK3, PRKDC	PI3K inhibitor
manumycin-a	trt_cp	BRD-K78599730	FNTA, IKBKB	Farnesyltransferase inhibitor, NFkB pathway inhibitor
rutin	trt_cp	BRD-K20482099	AKR1C3, AKR1B1, F10	Antioxidant, Capillary stabilizing agent, Nitric oxide scavenger
tyrphostin-AG-1296	trt_cp	BRD-K76064317	FLT3	FLT3 inhibitor
prostaglandin-e1	trt_cp	BRD-K52459643	PTGER1, PTGER2, CATSPER1, CATSPER2, CATSPER3, CATSPER4, PTGDR, PTGER4, PTGIR	Prostanoid receptor agonist

carbacyclin	trt_cp	BRD-K27499107	PPARD, PTGDR, PTGER1, PTGER2, PTGER3, PTGER4, PTGFR, PTGIR, TBXA2R	IP receptor activator, PPAR receptor agonist
CGP-53353	trt_cp	BRD-K32292990	EGFR, PRKCB	EGFR inhibitor, PKC inhibitor
dextromethorphan	trt_cp	BRD-K33211335	SIGMAR1, CHRNA2, CHRNA3, CHRNA4, CHRNA7, CHRNB2, CHRNB4, CYBA, CYBB, CYP3A5, GRIN1, GRIN3A, NCF1, NCF2, NCF4, OPRD1, OPRK1, OPRM1, PGRMC1, RAC1, RAC2, SLC6A2, SLC6A4	Glutamate receptor antagonist, Sigma receptor agonist
GTP-14564	trt_cp	BRD-K16664969	FLT3, CSF1R, KIT, PDGFRB	FLT3 inhibitor, Tyrosine kinase inhibitor
hispidin	trt_cp	BRD-K07325606	PREP, PRKCB, PTGS2, XDH	PKC inhibitor
IKK-16	trt_cp	BRD-K14618467	IKBKB	IKK inhibitor
IKK-2-inhibitor-V	trt_cp	BRD-K74305673	IKBKB	IKK inhibitor, NFkB pathway inhibitor
kavain	trt_cp	BRD-A75455249	MTOR	Calcium channel modulator, Sodium channel blocker
LY-303511	trt_cp	BRD-K22385716	CSNK2A1, CSNK2A2, CSNK2B, MTOR	Casein kinase inhibitor, MTOR inhibitor, PI3K inhibitor
MEK1-2-inhibitor	trt_cp	BRD-K12244279	MAP2K1, MAP2K2	MEK inhibitor
ODQ	trt_cp	BRD-K26015241	GUCY1A2, GUCY1A3, GUCY1B3	Guanylyl cyclase inhibitor
oxindole-I	trt_cp	BRD-K51816706	AKT1, KDR, PDPK1, RET	VEGFR inhibitor
PD-198306	trt_cp	BRD-K88677950	MAP2K1, MAP2K2, MAPK1, MAPK3	MAP kinase inhibitor, MEK inhibitor
PD-98059	trt_cp	BRD-K62810658	MAP2K1, AKT1, CHEK1, GSK3B, LCK, MAP2K2, MAPK1, MAPK11, MAPK12, MAPK14, MAPK3, MAPK8, PRKCA, RAF1, ROCK1, RPS6KB1, SGK1	MEK inhibitor, MAP kinase inhibitor
D-64406	trt_cp	BRD-K27665173	FLT3, PDGFRA, PDGFRB	PDGFR receptor inhibitor
PIT	trt_cp	BRD-K43978949	P2RY1	Purinergic receptor antagonist
midostaurin	trt_cp	BRD-K13646352	FLT3, KIT, CCNB1, FLT1, KDR, PDGFRB, PRKCA, PRKCG, VEGFA	FLT3 inhibitor, KIT inhibitor, PKC inhibitor

pseudoephedrine	trt_cp	BRD-K84175871	ADRA1A, ADRA2A, ADRB1, ADRB2, ATF1, ATF2, ATF3, ATF4, ATF5, ATF6, ATF7, CXCL8, FOS, HRH1, IL2, JDP2, JUN, NFATC1, SLC6A2, SLC6A3, SLC6A4, TNF	Adrenergic receptor agonist
parthenolide	trt_cp	BRD-K98548675	ADIPOR2, IKBKB, RELA	NFkB pathway inhibitor, Adiponectin receptor agonist
quercetin	trt_cp	BRD-K97399794	PIK3CG, AKR1B1, ATP5A1, ATP5B, ATP5C1, CYP2C8, EGFR, GAA, HCK, HIBCH, MAOA, PIM1, PTPN1, SCN5A, SIRT1, STK17B, UGT3A1, XDH	Polar auxin transport inhibitor
SB-225002	trt_cp	BRD-K61323504	CXCR2	CC chemokine receptor antagonist
IKK-2-inhibitor	trt_cp	BRD-K26373640	IKBKB	IKK inhibitor, SYK inhibitor
SA-792728	trt_cp	BRD-K20755323	SPHK1, VCP	Sphingosine kinase inhibitor
SQ-22536	trt_cp	BRD-A56987319	ADCY1, ADCY5	Adenylyl cyclase inhibitor
semaxanib	trt_cp	BRD-K63504947	KDR, FLT1, KIT, PDGFRB, FGFR1, FLT3, MET, PDGFRA, RET	VEGFR inhibitor
tranilast	trt_cp	BRD-K19533706	HPGDS, HRH1, IDO1, IFNG, IL10, IL2, IL4, SLC22A12, TGFB1, TNF, TRPV2	Angiogenesis inhibitor
U-0126	trt_cp	BRD-K18787491	AKT1, CHEK1, GSK3B, JAK2, LCK, MAP2K1, MAP2K2, MAP2K7, MAPK1, MAPK11, MAPK12, MAPK14, MAPK8, PRKCA, RAF1, ROCK1, RPS6KB1, SGK1	MEK inhibitor
U-46619	trt_cp	BRD-K18757346	PTGDR, PTGDR2, PTGER1, PTGER2, PTGER3, PTGER4, PTGFR, PTGIR, TBXA2R	Thromboxane receptor agonist
YC-1	trt_cp	BRD-K60476892	HIF1A, GUCY1A2, GUCY1A3, GUCY1B3	Guanylyl cyclase activator
dactolisib	trt_cp	BRD-K12184916	MTOR, PIK3CA, PIK3CG, PIK3CD, ATR, PIK3CB	MTOR inhibitor, PI3K inhibitor, Protein kinase inhibitor
selumetinib	trt_cp	BRD-K57080016	MAP2K1, MAP2K2	MEK inhibitor
AS-605240	trt_cp	BRD-K41895714	MAOB, PIK3CA, PIK3CB, PIK3CD, PIK3CG	PI3K inhibitor

AZD-8055	trt_cp	BRD-K69932463	MTOR	MTOR inhibitor
AKT-inhibitor-1-2	trt_cp	BRD-K04887706	AKT1, AKT2, AKT3	AKT inhibitor
BX-795	trt_cp	BRD-K47983010	PDPK1, CDK2, CHEK1, GSK3B, IKBKE, KDR, PDK1, TBK1	IKK inhibitor
KU-0060648	trt_cp	BRD-K09499853	PIK3CA, PIK3CB, PIK3CD, PIK3CG, PRKDC	DNA dependent protein kinase inhibitor, PI3K inhibitor
KU-0063794	trt_cp	BRD-K67566344	MTOR	MTOR inhibitor
MK-2206	trt_cp	BRD-K68065987	AKT1, AKT2, AKT3	AKT inhibitor
PI-103	trt_cp	BRD-K67868012	PIK3CA, PIK3CG, MTOR, PIK3CB, PIK3CD, PRKDC	MTOR inhibitor, PI3K inhibitor
PKCbeta-inhibitor	trt_cp	BRD-K89687904	PRKCB	PKC inhibitor
fostamatinib	trt_cp	BRD-K20285085	SYK, FLT3, RET	SYK inhibitor
TG-101348	trt_cp	BRD-K12502280	JAK2, FLT3, BRD4, JAK1, JAK3, RET, TYK2	FLT3 inhibitor, JAK inhibitor
TPCA-1	trt_cp	BRD-K51575138	IKBKB	IKK inhibitor
auranofin	trt_cp	BRD-A79465854	IKBKB, PRDX5, TRPA1, TXNRD1, TXNRD2	NFkB pathway inhibitor
forskolin	trt_cp	BRD-A70449690	ADCY2, ADCY5, GNAS	Adenylyl cyclase activator
temsirolimus	trt_cp	BRD-A62025033	MTOR, PTEN	MTOR inhibitor
myricetin	trt_cp	BRD-K43149758	PIK3CG, AR, CYP3A4	Androgen receptor agonist, Cytochrome P450 inhibitor
HG-6-64-01	trt_cp	BRD-U37049823	ABL1, BRAF, CSF1R, EGFR, FGFR1, FLT3, KIT, MAPK11, PDGFRB, RET	RAF inhibitor
treprostinil	trt_cp	BRD-A67438293	PTGIR, CYP2C8, P2RY12, PPARD, PTGDR, PTGER1, PTGER2, PTGER3, PTGER4	Prostacyclin analog
iloprost	trt_cp	BRD-A45664787	PTGIR, PTGER1, PTGER2, PDE4A, PDE4B, PDE4C, PDE4D, PLAT, PTGDR, PTGER3, PTGER4, PTGFR, TBXA2R	Platelet aggregation inhibitor, Prostanoid receptor agonist
mesalazine	trt_cp	BRD-K28849549	PTGS1, PPARG, PTGS2, ALOX5, CHUK, CTNNB1, IKBKB, MPO, NAT1	Cyclooxygenase inhibitor, Lipoxygenase inhibitor, Prostanoid receptor antagonist

sirolimus	trt_cp	BRD-K89626439	MTOR, FKBP1A, CCR5, FGF2	MTOR inhibitor
tozasertib	trt_cp	BRD-K59369769	AURKA, AURKB, ABL1, AURKC, BCR, FLT3, JAK2, DDR2, LCK	Aurora kinase inhibitor, BCR-ABL kinase inhibitor, FLT3 inhibitor, JAK inhibitor
parthenolide	trt_cp	BRD-K28120222	ADIPOR2, IKBKB, RELA	NFkB pathway inhibitor
sorafenib	trt_cp	BRD-K49810818	RET, BRAF, FLT3, KDR, RAF1, FLT1, FLT4, KIT, DDR2, FGFR1, PDGFRB, CYP2B6, CYP2C8, CYP3A5, PDGFB, SLCO1B3	FLT3 inhibitor, KIT inhibitor, PDGFR receptor inhibitor, RAF inhibitor, VEGFR inhibitor, RET tyrosine kinase inhibitor
PD-184352	trt_cp	BRD-K05104363	MAP2K1, MAP2K2, MAP3K1, MAP3K2	MEK inhibitor
deforolimus	trt_cp	BRD-K29733039	MTOR	MTOR inhibitor
PD-0325901	trt_cp	BRD-K49865102	MAP2K1, MAP2K2	MEK inhibitor, MAP kinase inhibitor, Protein kinase inhibitor
tandutinib	trt_cp	BRD-K89162000	FLT3, KIT, PDGFRA, PDGFRB, CSF1R, PDGFD	FLT3 inhibitor, KIT inhibitor, PDGFR receptor inhibitor
ZSTK-474	trt_cp	BRD-K63068307	PIK3CG, PIK3CA, PIK3CB, PIK3CD	PI3K inhibitor
TG100-115	trt_cp	BRD-K64785675	PIK3CG, PIK3CA, PIK3CB, PIK3CD	-666
sulfasalazine	trt_cp	BRD-K10670311	PTGS1, PTGS2, ACAT1, ALOX5, CHUK, IKBKB, PLA2G1B, PPARG, SLC46A1, SLC7A11, TBXAS1	Antirheumatic, NFkB pathway inhibitor
KIN001-244	trt_cp	BRD-K09186807	PDPK1	Phosphoinositide dependent kinase inhibitor
A-443644	trt_cp	BRD-K38615104	AKT1, AKT2, AKT3, CDC42BPB, GSK3B, PHKG1, PKIA, PRKACA	AKT inhibitor
torin-1	trt_cp	BRD-K40175214	MTOR, PIK3CA	MTOR inhibitor, PI3K inhibitor
GDC-0941	trt_cp	BRD-K52911425	PIK3CG, PIK3CA, PIK3CB, PIK3CD	PI3K inhibitor
torin-2	trt_cp	BRD-K68174511	MTOR	MTOR inhibitor
QL-X-138	trt_cp	BRD-U33728988	BTK, JAK3, MKNK2, MTOR, PRKDC	MTOR inhibitor
AS-604850	trt_cp	BRD-K63915849	PIK3CG, PIK3CA	PI3K inhibitor

withaferin-a	trt_cp	BRD-A52193669	ACHE, BCHE, IKBKB	IKK inhibitor
guggulsterone	trt_cp	BRD-A59808129	NR1H4, PGR, AR, ESR1, IKBKB, NR1I2, NR3C1, NR3C2	Cholesterol inhibitor, Estrogen receptor agonist, FXR antagonist, IKK inhibitor, Pregnane X receptor agonist, Progesterone receptor agonist
OSI-027	trt_cp	BRD-K94294671	MTOR	MTOR inhibitor
tyrphostin-AG-1295	trt_cp	BRD-K57926513	FLT3, KDR, PDGFRA, PDGFRB	PDGFR receptor inhibitor
GR-235	trt_cp	BRD-K26674531	ESR1, IKBKB, NR1H4, NR1I2, PGR	Estrogen receptor agonist, FXR antagonist, Progesterone receptor agonist
WYE-125132	trt_cp	BRD-A45498368	MTOR, PIK3CA	MTOR inhibitor
enzastaurin	trt_cp	BRD-K79404599	PRKCB, AKT1, GSK3B, PRKCA, PRKCD, PRKCG	PKC inhibitor
AZD-6482	trt_cp	BRD-K58772419	PIK3CD, PIK3CG, PIK3CA, PIK3CB	PI3K inhibitor
BMS-345541	trt_cp	BRD-K13566078	IKBKB, CHUK	IKK inhibitor
BX-912	trt_cp	BRD-K49669041	PDPK1, AKT2, CDK2, CHEK1, GSK3B, KDR, PDK1	Pyruvate dehydrogenase kinase inhibitor
quizartinib	trt_cp	BRD-K93918653	FLT3, CSF1R, KIT, RET, PDGFRA, PDGFRB	FLT3 inhibitor
lestaurtinib	trt_cp	BRD-K23192422	FLT3, NTRK1, JAK2, NTRK2, NTRK3	FLT3 inhibitor, Growth factor receptor inhibitor, JAK inhibitor
linifanib	trt_cp	BRD-K99749624	CSF1R, KDR, PDGFRB, FLT1, FLT3, FLT4, CSF1, KIT, PDGFRA, RET, TEK	PDGFR receptor inhibitor, VEGFR inhibitor
bosutinib	trt_cp	BRD-K99964838	ABL1, SRC, BCR, CAMK2G, LYN, CAMK1D, CDK2, FRK, FYN, HCK, MAP2K1, MAP2K2, MAP3K2, MAP4K5, STK10, STK24, STK4, TNK2, TXK	ABL inhibitor, BCR-ABL kinase inhibitor, SRC inhibitor
cediranib	trt_cp	BRD-K86930074	KDR, FLT1, FLT4, KIT, PDGFRB, CSF1R, FLT3, PDGFRA	KIT inhibitor, VEGFR inhibitor
dovitinib	trt_cp	BRD-K85402309	EGFR, FGFR3, PDGFRB, CSF1R, FGFR1, FGFR2, FLT1, FLT3, FLT4, INSR, KDR, KIT, PDGFRA	EGFR inhibitor, FLT3 inhibitor, FGFR inhibitor, PDGFR receptor inhibitor, VEGFR inhibitor

sunitinib	trt_cp	BRD-M64432851	FLT3, KDR, KIT, FLT4, FLT1, PDGFRA, PDGFRB, RET, CSF1R, FGFR1	FLT3 inhibitor, KIT inhibitor, PDGFR receptor inhibitor, RET tyrosine kinase inhibitor, VEGFR inhibitor
XL-147	trt_cp	BRD-K95901403	PIK3CA, PIK3CD, PIK3CG	PI3K inhibitor
everolimus	trt_cp	BRD-K13514097	MTOR, CYP3A5, FKBP1A	MTOR inhibitor
TGX-221	trt_cp	BRD-A41692738	PIK3CB, PIK3CD	PI3K inhibitor
PIK-75	trt_cp	BRD-M16762496	PIK3CA, PIK3CB, PIK3CD, PIK3CG, PRKDC	DNA protein kinase inhibitor, PI3K inhibitor
WYE-354	trt_cp	BRD-K77008974	MTOR	MTOR inhibitor
AS-703026	trt_cp	BRD-K89014967	MAP2K1, MAP2K2	MEK inhibitor
sunitinib	trt_cp	BRD-K70511574	FLT3, KDR, KIT, FLT4, FLT1, PDGFRA, PDGFRB, RET, CSF1R, FGFR1	PLK inhibitor
rivaroxaban	trt_cp	BRD-K37130656	F10, CYP2J2, CYP3A5	Coagulation inhibitor
4-hydroxy-2-nonenal	trt_cp	BRD-A15914070	IKBKB	Cytotoxic lipid peroxidation product
GSK-1059615	trt_cp	BRD-K06750613	PIK3CA, PIK3CG	PI3K inhibitor
aspirin	trt_cp	BRD-K11433652	PTGS1, PTGS2, TP53, AKR1C1, ASIC3, EDNRA, HSPA5, IKBKB, NFKB1, NFKB2, NFKBIA, PRKAA1, PRKAA2, PRKAB1, PRKAB2, PRKAG1, PRKAG2, PRKAG3, RPS6KA3	Cyclooxygenase inhibitor
staurosporine	trt_cp	BRD-K17953061	CDK2, GSK3B, CAMK2B, CDK1, CDK5, CHEK1, CHRM1, CHRM2, CHRM4, CSK, DAPK1, GPR35, IKBKB, ITK, LCK, LRRK2, MAP2K4, MAP2K6, MAPKAPK2, PAK2, PDPK1, PHKG2, PIK3CG, PIM1, PKN1, PRKACB, PRKCI, PRKCQ, RPS6KA1, STK3, SYK, TNIK, ZAP70	PKC inhibitor
prostratin	trt_cp	BRD-K91145395	PRKCA, PRKCB, PRKCD, PRKCE, PRKCG, PRKCH, PRKCQ	PKC activator
vidarabine	trt_cp	BRD-K72093121	ADCY5	Antiviral

AKT2	trt_oe	ccsbBroad304_00047
CXCR2	trt_oe	ccsbBroad304_00856
F10	trt_oe	ccsbBroad304_06188
GNG4	trt_oe	ccsbBroad304_00657
IKBKG	trt_oe	ccsbBroad304_01945
IL13	trt_oe	ccsbBroad304_00860
LSP1	trt_oe	ccsbBroad304_06538
PDPK1	trt_oe	ccsbBroad304_01168
PIK3CB	trt_oe	ccsbBroad304_01202
PIK3CG	trt_oe	ccsbBroad304_06727
PRKCB	trt_oe	ccsbBroad304_01282
PRKCZ	trt_oe	ccsbBroad304_01284
EPO	trt_oe	ccsbBroad304_00512
HRAS	trt_oe	ccsbBroad304_00784
IKBKB	trt_oe	ccsbBroad304_00841
IL2	trt_oe	ccsbBroad304_00846
ADCY5	trt_sh.cgs	CGS001-111
EPO	trt_sh.cgs	CGS001-2056
AKT2	trt_sh.cgs	CGS001-208
F5	trt_sh.cgs	CGS001-2153
F10	trt_sh.cgs	CGS001-2159
F12	trt_sh.cgs	CGS001-2161
GPRC6A	trt_sh.cgs	CGS001-222545
FLT3	trt_sh.cgs	CGS001-2322
MTOR	trt_sh.cgs	CGS001-2475
GNG4	trt_sh.cgs	CGS001-2786
GRK5	trt_sh.cgs	CGS001-2869
HRAS	trt_sh.cgs	CGS001-3265
IKBKB	trt_sh.cgs	CGS001-3551
IL2	trt_sh.cgs	CGS001-3558
CXCR2	trt_sh.cgs	CGS001-3579
IL13	trt_sh.cgs	CGS001-3596
LSP1	trt_sh.cgs	CGS001-4046
MAF	trt_sh.cgs	CGS001-4094
PDPK1	trt_sh.cgs	CGS001-5170
PIK3CB	trt_sh.cgs	CGS001-5291
PIK3CG	trt_sh.cgs	CGS001-5294
POLR2K	trt_sh.cgs	CGS001-5440
PRKCB	trt_sh.cgs	CGS001-5579
PRKCZ	trt_sh.cgs	CGS001-5590
MAP2K2	trt_sh.cgs	CGS001-5605
PXN	trt_sh.cgs	CGS001-5829
IKBKG	trt_sh.cgs	CGS001-8517
CDC42	trt_sh.cgs	CGS001-998

AT-9283	trt_cp	BRD-K24576554	AURKA, AURKB, ABL1, BCR, FLT3, JAK2, JAK3, RPS6KA6, STK17A	JAK inhibitor, Aurora kinase inhibitor, ABL inhibitor, BCR-ABL kinase inhibitor, FLT3 inhibitor, Mitotic inhibitor, Protein kinase inhibitor
ENMD-2076	trt_cp	BRD-K68488863	AURKA, FLT3, KDR, PDGFRA, SRC, CSF1R, EPHA1, FGFR1, FGFR2, FGFR3, FLT4, KIT, PTK2	FLT3 inhibitor, VEGFR inhibitor, Aurora kinase inhibitor
PF-543	trt_cp	BRD-K79877282	SPHK1	Sphingosine kinase inhibitor

Supplementary Table S11. Connectivity map analysis for GSE111907 LUSC.

Name	Type	Perturbagen Id	Target	MOA
bisindolylmaleimide	trt_cp	BRD-K49448285	CCND1, CDK4, LRRK2, PDPK1, PIM1, PRKCA, PRKCB, PRKCI, PRKCZ	CDK inhibitor
colforsin	trt_cp	BRD-A55416093	ADCY2, ADCY5, GNAS	Adenylyl cyclase activator, Adenylate cyclase stimulant
digoxin	trt_cp	BRD-A94756469	ATP1A1, ABCB1, RORC, SLCO1B3	ATPase inhibitor
dorsomorphin	trt_cp	BRD-K54233340	ACVR1, BMPR1A, BMPR1B, EPHA2, FKBP1A, FLT1, FLT3, KDR, LCK, MKNK1, PRKAA1, RPS6KA1, SRC	AMPK inhibitor
nicotine	trt_cp	BRD-K05395900	CHRNA10, CHRNA9, AOX1, CHAT, CHRNA2, CHRNA3, CHRNA4, CHRNA5, CHRNA6, CHRNA7, CHRNB2, CHRNB3, CHRNB4, CYP19A1, CYP2B6, TBXAS1, TRPA1	Acetylcholine receptor agonist
pepstatin	trt_cp	BRD-K13571841	CTSB, CTSD, CTSL, REN	Aspartic protease inhibitor
RS-102895	trt_cp	BRD-K83063356	CCR2	CCR antagonist
TCS-359	trt_cp	BRD-K81376179	FLT3	FLT3 inhibitor
tamoxifen	trt_cp	BRD-K04210847	ESR1, ESR2, CYP3A5, EBP, GPER1, PRKCA, PRKCB, PRKCD, PRKCE, PRKCG, PRKCI, PRKCQ, PRKCZ	Estrogen receptor antagonist, Selective estrogen receptor modulator (SERM)
YM-90709	trt_cp	BRD-K06712146	CSF2RB, IL5RA	IL5 inhibitor
ZM-39923	trt_cp	BRD-K40624912	JAK1, JAK3	JAK inhibitor
AG-490	trt_cp	BRD-K12357156	JAK2, JAK3, EGFR, STAT3	EGFR inhibitor, ErbB2 inhibitor, JAK inhibito
tyrphostin-AG-1296	trt_cp	BRD-K76064317	FLT3	FLT3 inhibitor
CGP-53353	trt_cp	BRD-K32292990	EGFR, PRKCB	EGFR inhibitor, PKC inhibitor

dextromethorphan	trt_cp	BRD-K33211335	SIGMAR1, CHRNA2, CHRNA3, CHRNA4, CHRNA7, CHRNB2, CHRNB4, CYBA, CYBB, CYP3A5, GRIN1, GRIN3A, NCF1, NCF2, NCF4, OPRD1, OPRK1, OPRM1, PGRMC1, RAC1, RAC2, SLC6A2, SLC6A4	Glutamate receptor antagonist, Sigma recepto agonist
gamma-linolenic-acid	trt_cp	BRD-K18059238	TBXAS1	Cyclooxygenase inhibitor, Prostanoid recepto agonist
GTP-14564	trt_cp	BRD-K16664969	FLT3, CSF1R, KIT, PDGFRB	FLT3 inhibitor, Tyrosine kinase inhibitor
hispidin	trt_cp	BRD-K07325606	PREP, PRKCB, PTGS2, XDH	PKC inhibitor
JAK3-inhibitor-I	trt_cp	BRD-K72541103	JAK3	JAK inhibitor
JAK3-Inhibitor-II	trt_cp	BRD-K52850071	EGFR, ALK, JAK1, JAK2, JAK3	JAK inhibitor
JAK3-inhibitor-V	trt_cp	BRD-K95676198	JAK3	JAK inhibitor
JAK3-inhibitor-VI	trt_cp	BRD-K04546108	JAK3	JAK inhibitor
leflunomide	trt_cp	BRD-K78692225	DHODH, AHR, CYP2C19, JAK3, PTK2B, STAT6	Dihydroorotate dehydrogenase inhibitor, PDGFR receptor inhibitor
MDL-28170	trt_cp	BRD-K43245338	CAPN1, CTSB, CTSL	Calpain inhibitor
ozagrel	trt_cp	BRD-K19525698	TBXAS1	Thromboxane synthase inhibitor
D-64406	trt_cp	BRD-K27665173	FLT3, PDGFRA, PDGFRB	PDGFR receptor inhibitor
midostaurin	trt_cp	BRD-K13646352	FLT3, KIT, CCNB1, FLT1, KDR, PDGFRB, PRKCA, PRKCG, VEGFA	FLT3 inhibitor, KIT inhibitor, PKC inhibitor
pseudoephedrine	trt_cp	BRD-K84175871	ADRA1A, ADRA2A, ADRB1, ADRB2, ATF1, ATF2, ATF3, ATF4, ATF5, ATF6, ATF7, CXCL8, FOS, HRH1, IL2, JDP2, JUN, NFATC1, SLC6A2, SLC6A3, SLC6A4, TNF	Adrenergic receptor agonist
picotamide	trt_cp	BRD-K67277431	TBXA2R, TBXAS1	Thromboxane receptor antagonist, Thromboxane synthase inhibitor
RS-504393	trt_cp	BRD-K87510569	CCR2, CCL2	CC chemokine receptor antagonist
tretinoin	trt_cp	BRD-K06926592	RARG, RORB, ALDH1A1, ALDH1A2, GPRC5A, NR0B1, NR2C2, PPARD, RARA, RARB, RARRES1, RORC, RXRB, RXRG	Retinoid receptor agonist, Retinoid receptor ligand

semaxanib	trt_cp	BRD-K63504947	KDR, FLT1, KIT, PDGFRB, FGFR1, FLT3, MET, PDGFRA, RET	VEGFR inhibitor
T-0901317	trt_cp	BRD-K23383398	NR1H3, NR1H2, ABCA1, NCOA1, NCOA2, NR1I2, RORA, RORC, RXRB	LXR agonist, ABC transporter expression enhancer, ROR inverse agonist
tranilast	trt_cp	BRD-K19533706	HPGDS, HRH1, IDO1, IFNG, IL10, IL2, IL4, SLC22A12, TGFB1, TNF, TRPV2	Angiogenesis inhibitor
NF-449	trt_cp	BRD-K36324071	P2RX1	Purinergic receptor antagonist
PKCbeta-inhibitor	trt_cp	BRD-K89687904	PRKCB	PKC inhibitor
quinine	trt_cp	BRD-U94846492	KCNN4, ABCB1, CYP2D6, GP9, KCNB2, SLC29A4	Hemozoin biocrystallization inhibitor
fostamatinib	trt_cp	BRD-K20285085	SYK, FLT3, RET	SYK inhibitor
SID-26681509	trt_cp	BRD-K08417745	CTSL	Cathepsin inhibitor
TG-101348	trt_cp	BRD-K12502280	JAK2, FLT3, BRD4, JAK1, JAK3, RET, TYK2	FLT3 inhibitor, JAK inhibitor
atorvastatin	trt_cp	BRD-U88459701	HMGCR, DPP4, AHR, CYP3A5, FASLG	HMGCR inhibitor
forskolin	trt_cp	BRD-A70449690	ADCY2, ADCY5, GNAS	Adenylyl cyclase activator
HG-6-64-01	trt_cp	BRD-U37049823	ABL1, BRAF, CSF1R, EGFR, FGFR1, FLT3, KIT, MAPK11, PDGFRB, RET	RAF inhibitor
tozasertib	trt_cp	BRD-K59369769	AURKA, AURKB, ABL1, AURKC, BCR, FLT3, JAK2, DDR2, LCK	Aurora kinase inhibitor, BCR-ABL kinase inhibitor, FLT3 inhibitor, JAK inhibitor
sorafenib	trt_cp	BRD-K49810818	RET, BRAF, FLT3, KDR, RAF1, FLT1, FLT4, KIT, DDR2, FGFR1, PDGFRB, CYP2B6, CYP2C8, CYP3A5, PDGFB, SLCO1B3	FLT3 inhibitor, KIT inhibitor, PDGFR recepto inhibitor, RAF inhibitor, VEGFR inhibitor, RET tyrosine kinase inhibitor
tandutinib	trt_cp	BRD-K89162000	FLT3, KIT, PDGFRA, PDGFRB, CSF1R, PDGFD	FLT3 inhibitor, KIT inhibitor, PDGFR recepto inhibitor
sitagliptin	trt_cp	BRD-K19416115	DPP4, CYP2C8, FASLG, HMGCR, SLC22A8	Dipeptidyl peptidase inhibitor
tofacitinib	trt_cp	BRD-K31283835	JAK3, JAK1, JAK2, CYP2C19, TYK2	JAK inhibitor
sulfasalazine	trt_cp	BRD-K10670311	PTGS1, PTGS2, ACAT1, ALOX5, CHUK, IKBKB, PLA2G1B, PPARG, SLC46A1, SLC7A11, TBXAS1	Antirheumatic, NFkB pathway inhibitor
QL-X-138	trt_cp	BRD-U33728988	BTK, JAK3, MKNK2, MTOR, PRKDC	MTOR inhibitor

tyrphostin-AG-1295	trt_cp	BRD-K57926513	FLT3, KDR, PDGFRA, PDGFRB	PDGFR receptor inhibitor
ruxolitinib	trt_cp	BRD-K53972329	JAK1, JAK2, TYK2, JAK3	JAK inhibitor
isotretinoin	trt_cp	BRD-K76723084	CYP2B6, CYP2C19, CYP2C8, CYP3A5, CYP3A7, NR2C2, PPARD, RARA, RARB, RARG, RORB, RORC	Retinoid receptor agonist
enzastaurin	trt_cp	BRD-K79404599	PRKCB, AKT1, GSK3B, PRKCA, PRKCD, PRKCG	PKC inhibitor
KIN001-055	trt_cp	BRD-K68407802	EGFR, JAK3	EGFR inhibitor, JAK inhibitor, Leukotriene inhibitor, Mediator release inhibitor
quizartinib	trt_cp	BRD-K93918653	FLT3, CSF1R, KIT, RET, PDGFRA, PDGFRB	FLT3 inhibitor
lestaurtinib	trt_cp	BRD-K23192422	FLT3, NTRK1, JAK2, NTRK2, NTRK3	FLT3 inhibitor, Growth factor receptor inhibitor, JAK inhibitor
linifanib	trt_cp	BRD-K99749624	CSF1R, KDR, PDGFRB, FLT1, FLT3, FLT4, CSF1, KIT, PDGFRA, RET, TEK	PDGFR receptor inhibitor, VEGFR inhibitor
bosutinib	trt_cp	BRD-K99964838	ABL1, SRC, BCR, CAMK2G, LYN, CAMK1D, CDK2, FRK, FYN, HCK, MAP2K1, MAP2K2, MAP3K2, MAP4K5, STK10, STK24, STK4, TNK2, TXK	ABL inhibitor, BCR-ABL kinase inhibitor, SRC inhibitor
cediranib	trt_cp	BRD-K86930074	KDR, FLT1, FLT4, KIT, PDGFRB, CSF1R, FLT3, PDGFRA	KIT inhibitor, VEGFR inhibitor
dovitinib	trt_cp	BRD-K85402309	EGFR, FGFR3, PDGFRB, CSF1R, FGFR1, FGFR2, FLT1, FLT3, FLT4, INSR, KDR, KIT, PDGFRA	EGFR inhibitor, FLT3 inhibitor, FGFR inhibitor, PDGFR receptor inhibitor, VEGFR inhibitor
sunitinib	trt_cp	BRD-M64432851	FLT3, KDR, KIT, FLT4, FLT1, PDGFRA, PDGFRB, RET, CSF1R, FGFR1	FLT3 inhibitor, KIT inhibitor, PDGFR recepto inhibitor, RET tyrosine kinase inhibitor, VEGFR inhibitor
sunitinib	trt_cp	BRD-K70511574	FLT3, KDR, KIT, FLT4, FLT1, PDGFRA, PDGFRB, RET, CSF1R, FGFR1	PLK inhibitor
prostratin	trt_cp	BRD-K91145395	PRKCA, PRKCB, PRKCD, PRKCE, PRKCG, PRKCH, PRKCQ	PKC activator
CD14	trt_oe	ccsbBroad304_00249		
CD19	trt_oe	ccsbBroad304_00250		
LSP1	trt_oe	ccsbBroad304_06538		
PRKCB	trt_oe	ccsbBroad304_01282		
RORC	trt_oe	ccsbBroad304_01411		
C2	trt_oe	ccsbBroad304_00184		
EGR3	trt_oe	ccsbBroad304_00488		

FASLG	trt_oe	ccsbBroad304_00088		
FGB	trt_oe	ccsbBroad304_00554		
IL2	trt_oe	ccsbBroad304_00846		
CFD	trt_sh.cgs	CGS001-1675		
EGR3	trt_sh.cgs	CGS001-1960		
FGB	trt_sh.cgs	CGS001-2244		
FLT3	trt_sh.cgs	CGS001-2322		
IL2	trt_sh.cgs	CGS001-3558		
FASLG	trt_sh.cgs	CGS001-356		
IL5	trt_sh.cgs	CGS001-3567		
INPP5D	trt_sh.cgs	CGS001-3635		
JAK3	trt_sh.cgs	CGS001-3718		
LSP1	trt_sh.cgs	CGS001-4046		
PRKCB	trt_sh.cgs	CGS001-5579		
RORC	trt_sh.cgs	CGS001-6097		
C2	trt_sh.cgs	CGS001-717		
CD14	trt_sh.cgs	CGS001-929		
CD19	trt_sh.cgs	CGS001-930		
AT-9283	trt_cp	BRD-K24576554	AURKA, AURKB, ABL1, BCR, FLT3, JAK2, JAK3, RPS6KA6, STK17A	JAK inhibitor, Aurora kinase inhibitor, ABL inhibitor, BCR-ABL kinase inhibitor, FLT3 inhibitor, Mitotic inhibitor, Protein kinase inhibitor
ENMD-2076	trt_cp	BRD-K68488863	AURKA, FLT3, KDR, PDGFRA, SRC, CSF1R, EPHA1, FGFR1, FGFR2, FGFR3, FLT4, KIT, PTK2	FLT3 inhibitor, VEGFR inhibitor, Aurora kinase inhibitor

Supplementary Figure S1. GSE87340 dataset gene set enrichment for pathway analysis for normal tissue and LUAD

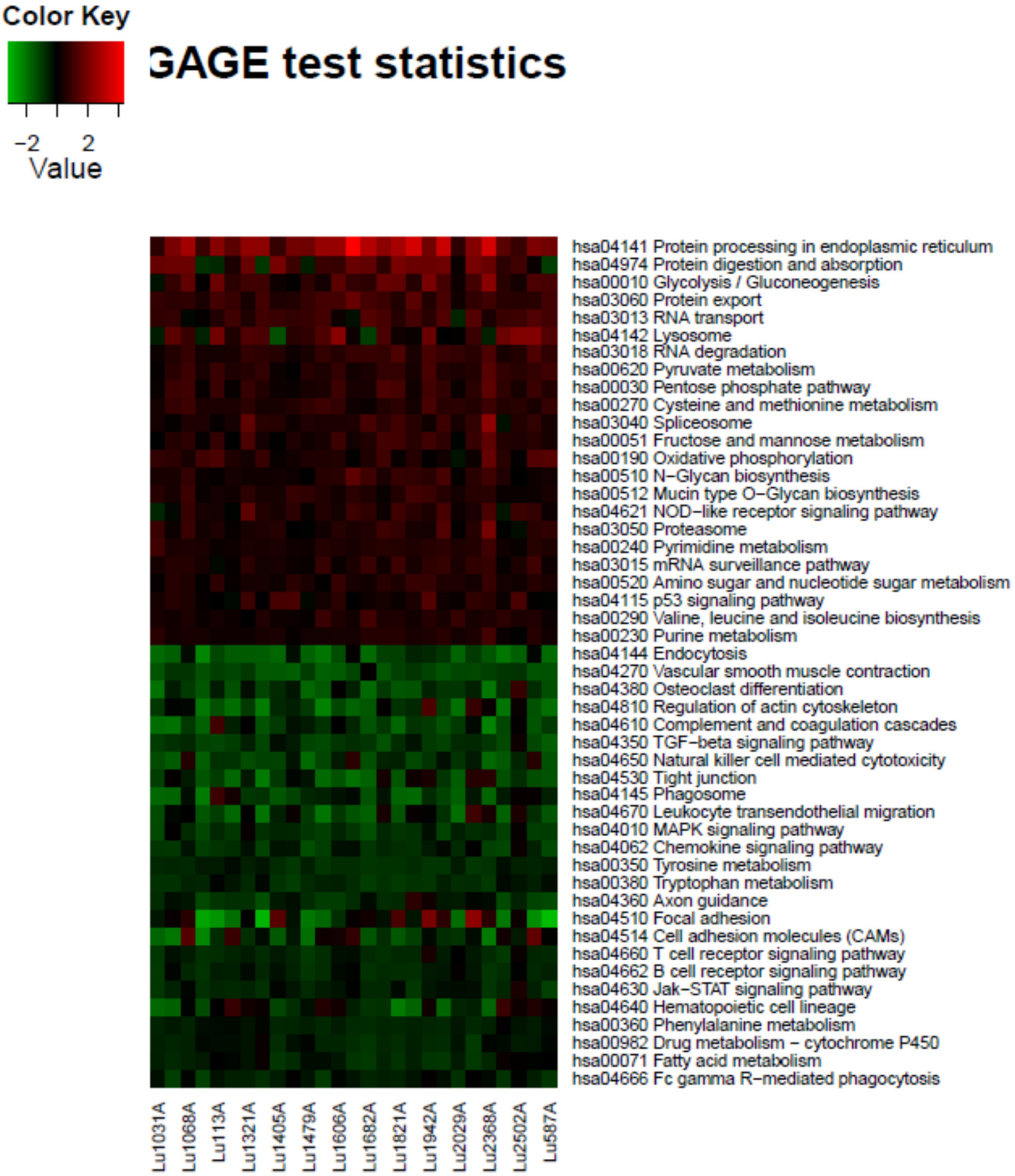

Supplementary Figure S2. GSE120622 dataset gene set enrichment for pathway analysis for normal tissue compared to LUAD

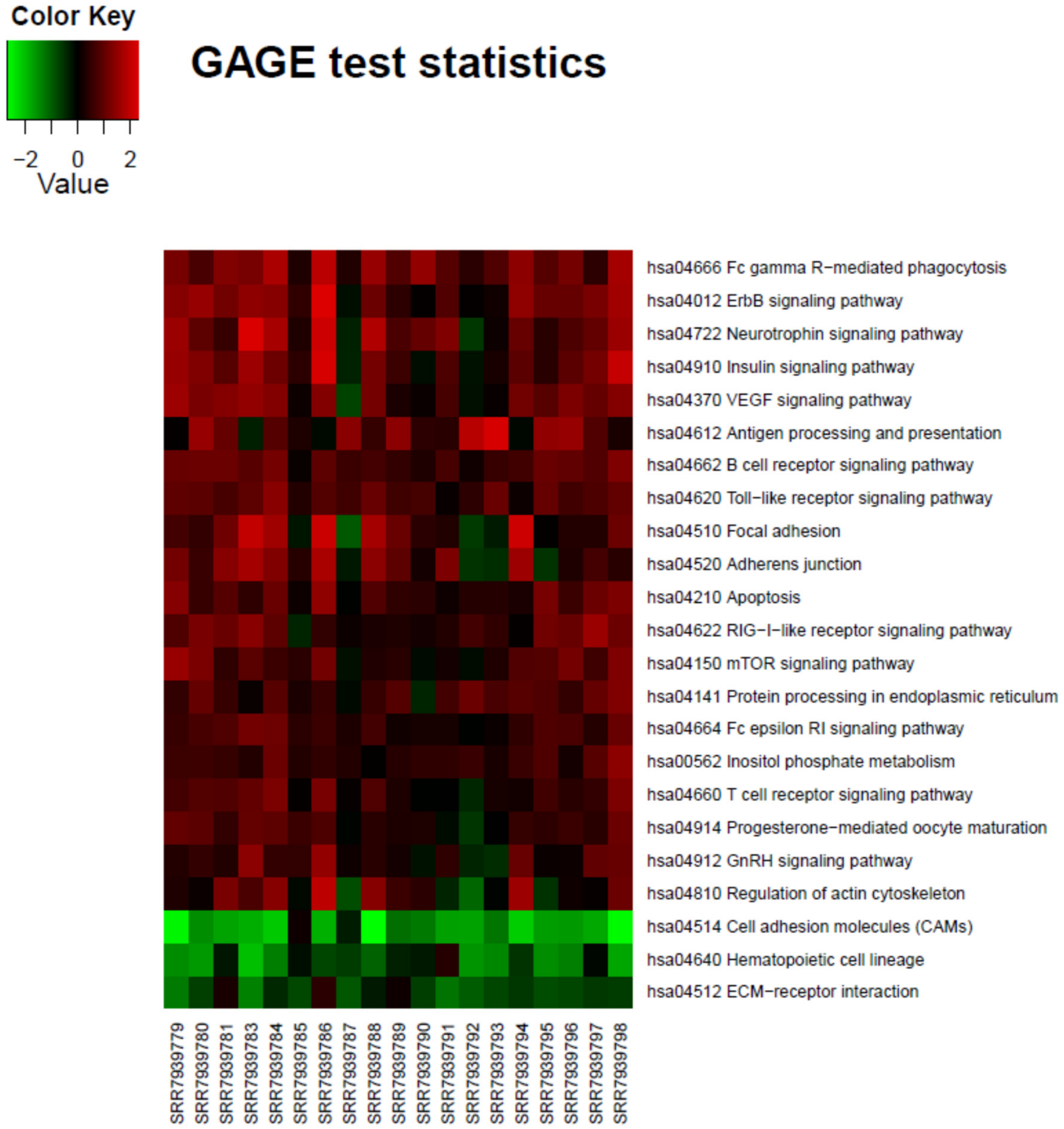

Supplementary Figure S3. gse120622 dataset gene set enrichment for pathway analysis for normal tissue compared to LUSC

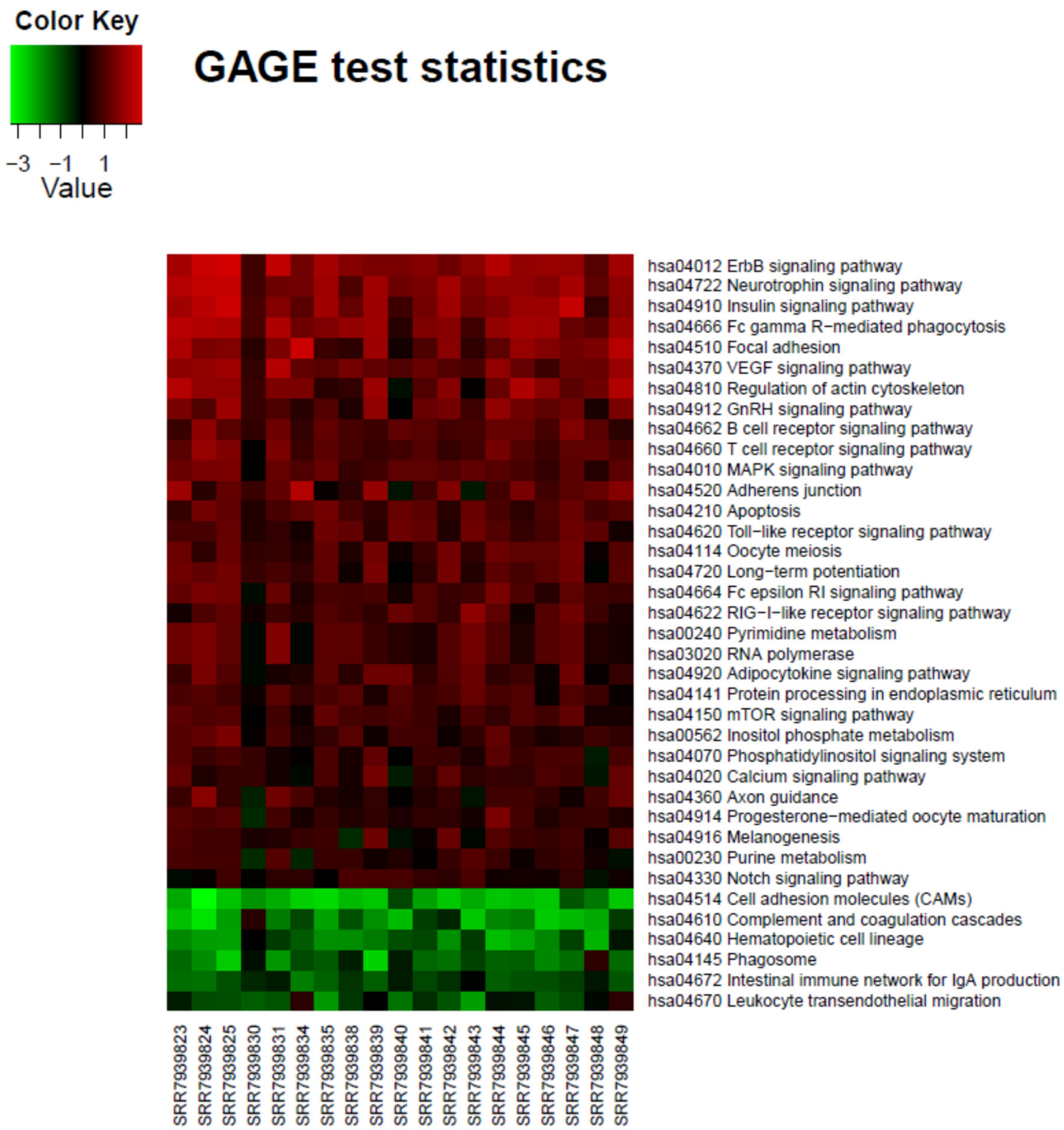

Supplementary Figure S4. GSE111907-1 dataset gene set enrichment for pathway analysis for normal tissue compared to LUAD

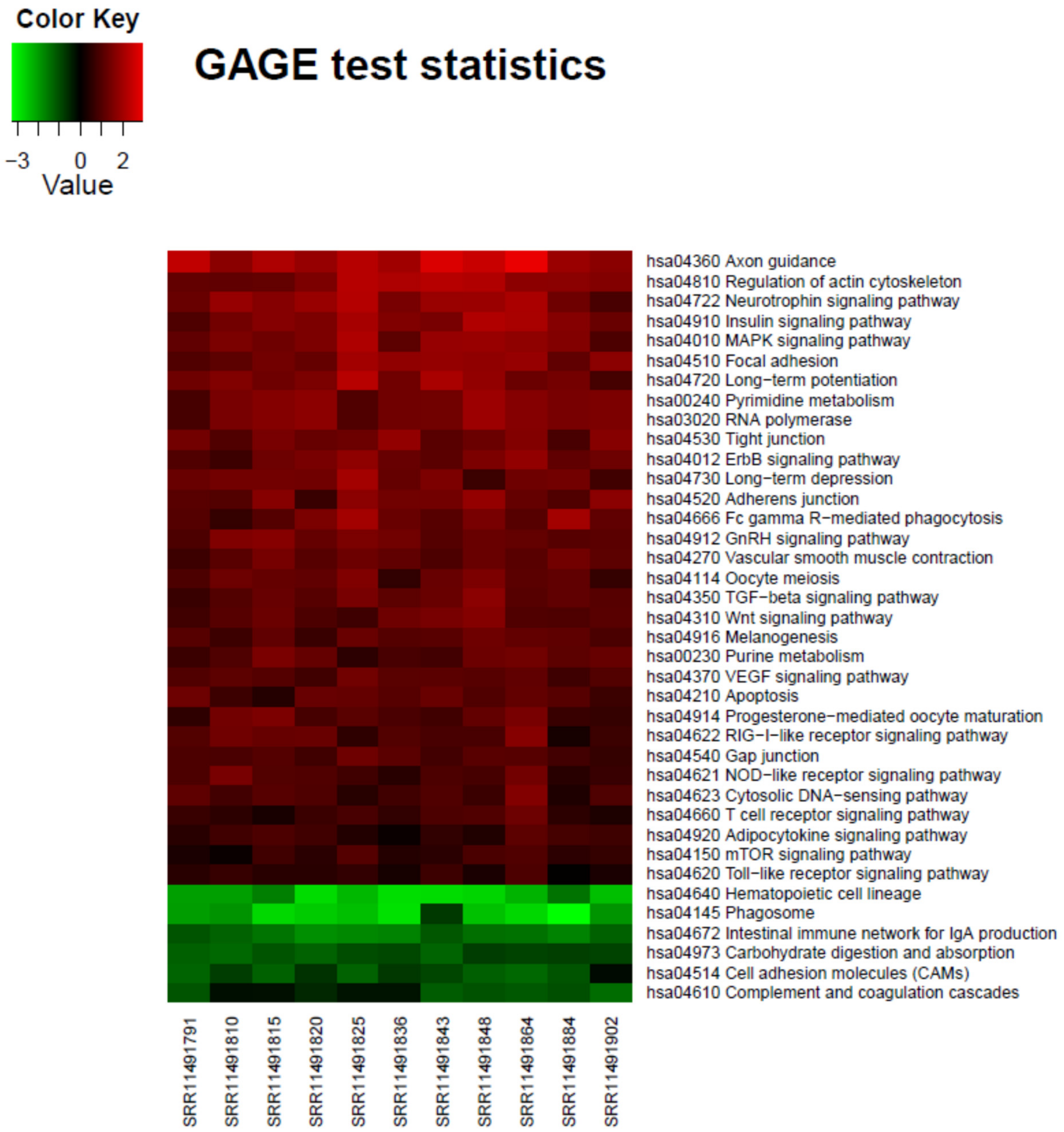

Supplementary Figure S5. GSE111907-2 dataset gene set enrichment for pathway analysis for normal tissue compared to LUSC

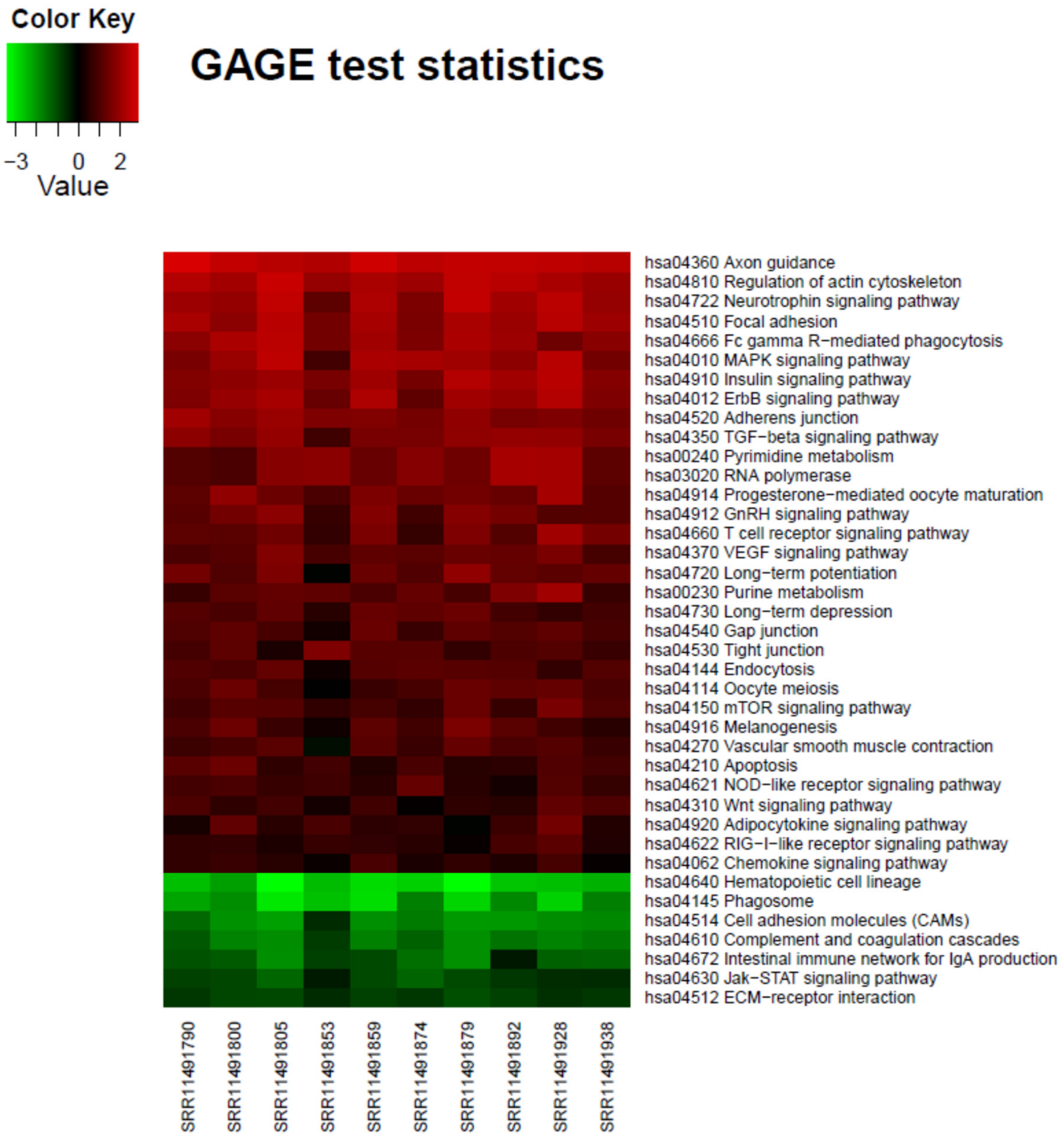

Supplementary Figure S6. Constructed network of GSE1119707-1 sorted fibroblast and endothelial cell from LUAD

GSE111907-1 sorted fibroblast

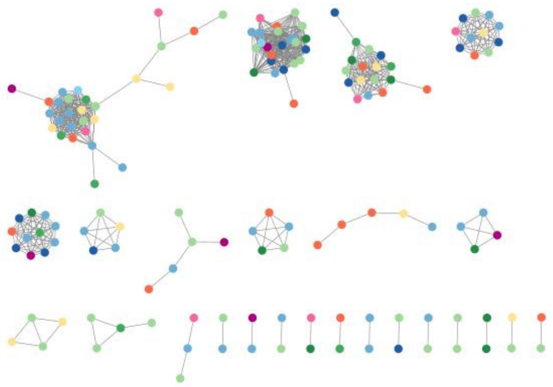

GSE111907-1 sorted endothelial cell

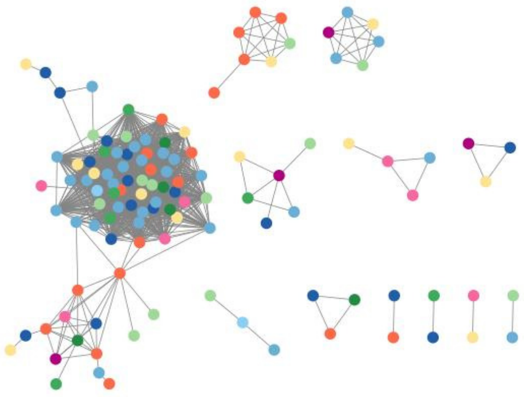

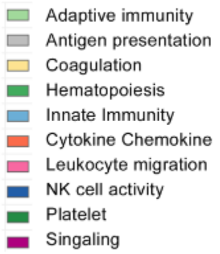

Supplementary Figure S7. Constructed network of GSE1119707-2 sorted fibroblast and endothelial cell from LUSC

GSE111907-2 sorted fibroblast

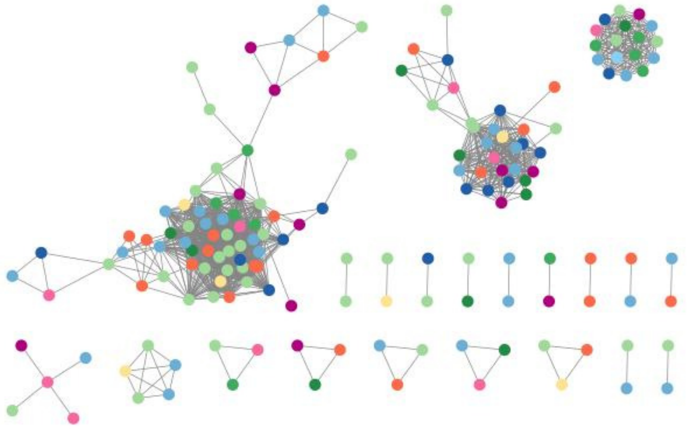

GSE111907-2 sorted endothelial cell

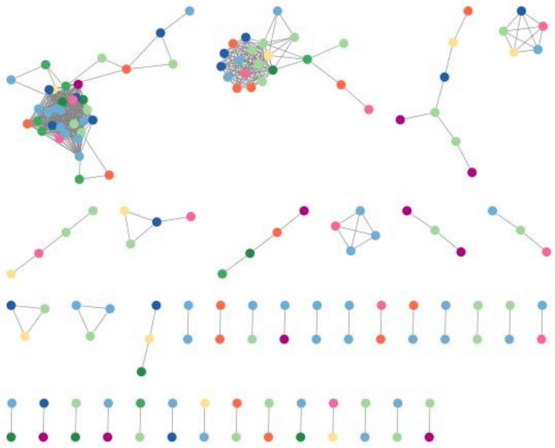

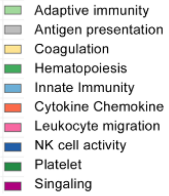

Supplementary Figure S8. Proportion of annotated gene function within the network GSE87340 and GSE1290622. Normal (blue), LUAD (red) and LUSC (green) are plotted

GSE87340

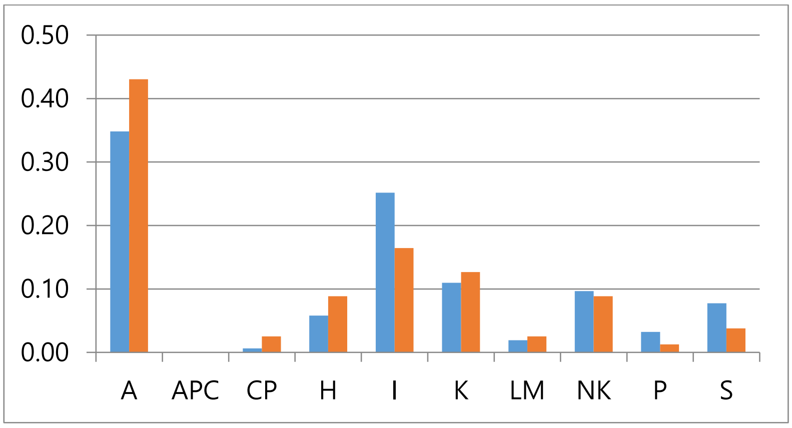

GSE120622

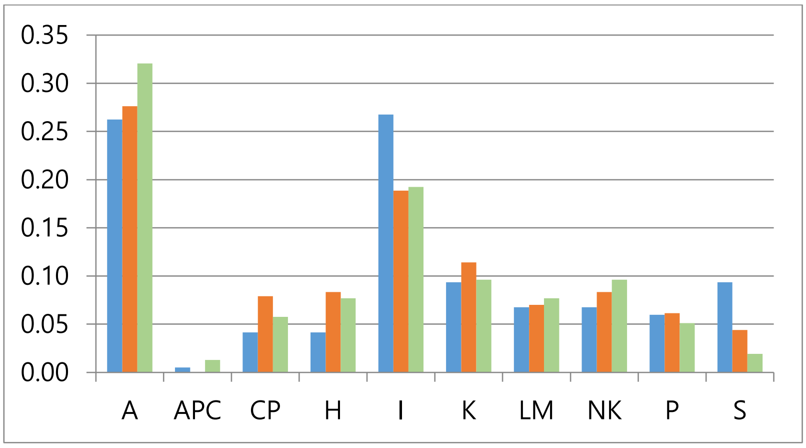

LUAD, adenocarcinoma non-small-cell lung cancer; LUSC, squamous non-small-cell lung carcinoma; A, adaptive immunity; APC, antigen presentation; CP, coagulation; H, hematopoiesis; I, innate immunity; K, cytokine and chemokine; LM, leukocyte migration; NK, NK cell; P, platelet; S, signaling

Supplementary Figure S9. Proportion of annotated gene function within the network GSE111907-1 and GSE111907-2. Normal (blue), LUAD (red) and LUSC (green) are plotted

GSE111907-1

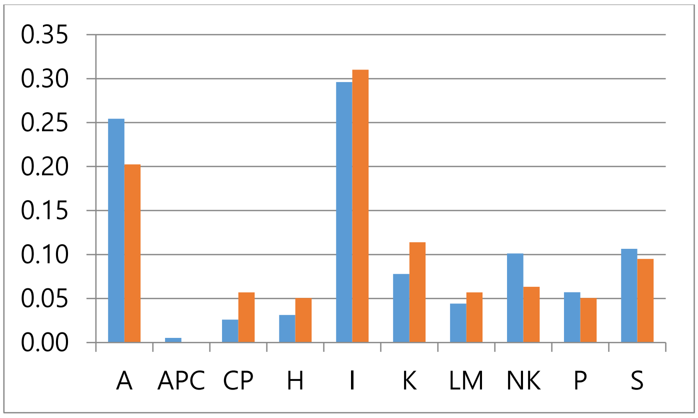

GSE111907-2

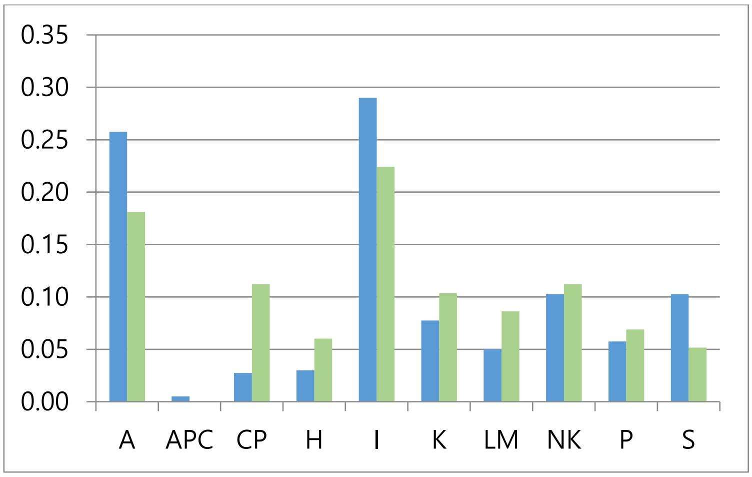

LUAD, adenocarcinoma non-small-cell lung cancer; LUSC, squamous non-small-cell lung carcinoma; A, adaptive immunity; APC, antigen presentation; CP, coagulation; H, hematopoiesis; I, innate immunity; K, cytokine and chemokine; LM, leukocyte migration; NK, NK cell; P, platelet; S, signaling

Supplementary Figure S10. Connectivity map analysis for network gene expression signatrures.

GSE120622 LUAD

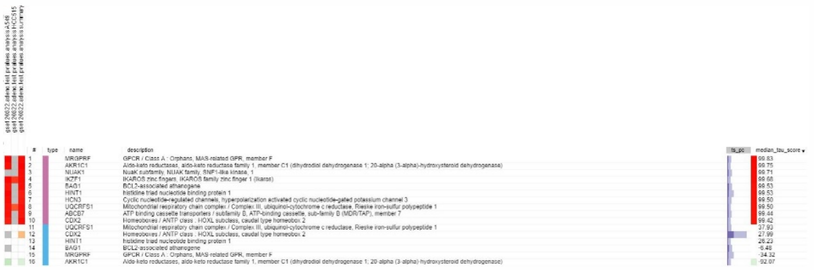

GSE120622 LUSC

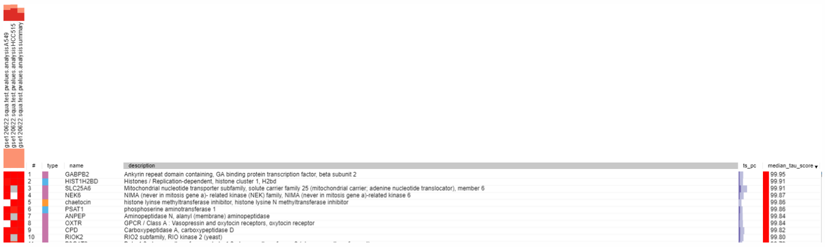

GSE111907-1 LUAD

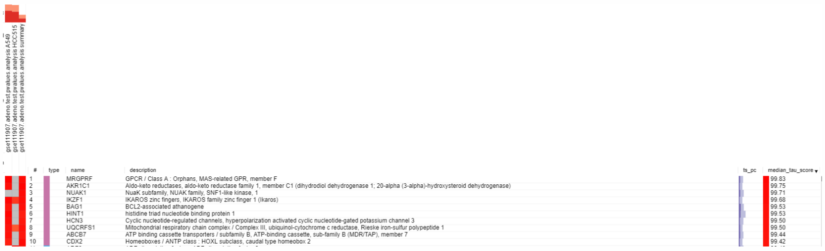

GSE111907-2 LUSC

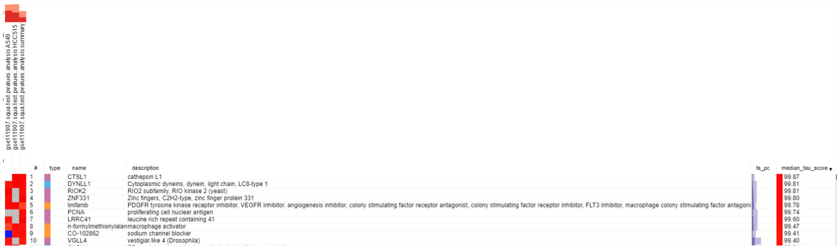

Supplement: Supplementary file 1 [file biomedicines-12-00628-s001.zip › biomedicines-2862177-supplementary.pdf]
